# B‐type natriuretic peptide‐guided treatment for heart failure

**DOI:** 10.1002/14651858.CD008966.pub2

**Published:** 2016-12-22

**Authors:** Julie McLellan, Carl J Heneghan, Rafael Perera, Alison M Clements, Paul P Glasziou, Karen E Kearley, Nicola Pidduck, Nia W Roberts, Sally Tyndel, F Lucy Wright, Clare Bankhead

**Affiliations:** University of OxfordNuffield Department of Primary Care Health SciencesOxfordUK; Bond UniversityCentre for Research in Evidence‐Based Practice (CREBP)University DriveGold CoastQueenslandAustralia4229; University of OxfordBodleian Health Care LibrariesKnowledge Centre, ORC Research Building, Old Road CampusOxfordOxfordshireUKOX3 7DQ; University of OxfordCancer Epidemiology Unit, Nuffield Department of Population HealthRichard doll BldgOld Road Campus, Roosevelt DriverOxfordUKOX3 7LF

## Abstract

**Background:**

Heart failure is a condition in which the heart does not pump enough blood to meet all the needs of the body. Symptoms of heart failure include breathlessness, fatigue and fluid retention. Outcomes for patients with heart failure are highly variable; however on average, these patients have a poor prognosis. Prognosis can be improved with early diagnosis and appropriate use of medical treatment, use of devices and transplantation. Patients with heart failure are high users of healthcare resources, not only due to drug and device treatments, but due to high costs of hospitalisation care. B‐type natriuretic peptide levels are already used as biomarkers for diagnosis and prognosis of heart failure, but could offer to clinicians a possible tool to guide drug treatment. This could optimise drug management in heart failure patients whilst allaying concerns over potential side effects due to drug intolerance.

**Objectives:**

To assess whether treatment guided by serial BNP or NT‐proBNP (collectively referred to as NP) monitoring improves outcomes compared with treatment guided by clinical assessment alone.

**Search methods:**

Searches were conducted up to 15 March 2016 in the Cochrane Central Register of Controlled Trials (CENTRAL) in the Cochrane Library; MEDLINE (OVID), Embase (OVID), the Database of Abstracts of Reviews of Effects (DARE) and the NHS Economic Evaluation Database in the Cochrane Library. Searches were also conducted in the Science Citation Index Expanded, the Conference Proceedings Citation Index on Web of Science (Thomson Reuters), World Health Organization International Clinical Trials Registry and ClinicalTrials.gov. We applied no date or language restrictions.

**Selection criteria:**

We included randomised controlled trials of NP‐guided treatment of heart failure versus treatment guided by clinical assessment alone with no restriction on follow‐up. Adults treated for heart failure, in both in‐hospital and out‐of‐hospital settings, and trials reporting a clinical outcome were included.

**Data collection and analysis:**

Two review authors independently selected studies for inclusion, extracted data and evaluated risk of bias. Risk ratios (RR) were calculated for dichotomous data, and pooled mean differences (MD) (with 95% confidence intervals (CI)) were calculated for continuous data. We contacted trial authors to obtain missing data. Using the Grading of Recommendations Assessment, Development and Evaluation (GRADE) approach, we assessed the quality of the evidence and GRADE profiler (GRADEPRO) was used to import data from Review Manager to create a 'Summary of findings' table.

**Main results:**

We included 18 randomised controlled trials with 3660 participants (range of mean age: 57 to 80 years) comparing NP‐guided treatment with clinical assessment alone. The evidence for all‐cause mortality using NP‐guided treatment showed uncertainty (RR 0.87, 95% CI 0.76 to 1.01; patients = 3169; studies = 15; low quality of the evidence), and for heart failure mortality (RR 0.84, 95% CI 0.54 to 1.30; patients = 853; studies = 6; low quality of evidence).

The evidence suggested heart failure admission was reduced by NP‐guided treatment (38% versus 26%, RR 0.70, 95% CI 0.61 to 0.80; patients = 1928; studies = 10; low quality of evidence), but the evidence showed uncertainty for all‐cause admission (57% versus 53%, RR 0.93, 95% CI 0.84 to 1.03; patients = 1142; studies = 6; low quality of evidence).

Six studies reported on adverse events, however the results could not be pooled (patients = 1144; low quality of evidence). Only four studies provided cost of treatment results, three of these studies reported a lower cost for NP‐guided treatment, whilst one reported a higher cost (results were not pooled; patients = 931, low quality of evidence). The evidence showed uncertainty for quality of life data (MD ‐0.03, 95% CI ‐1.18 to 1.13; patients = 1812; studies = 8; very low quality of evidence).

We completed a 'Risk of bias' assessment for all studies. The impact of risk of bias from lack of blinding of outcome assessment and high attrition levels was examined by restricting analyses to only low 'Risk of bias' studies.

**Authors' conclusions:**

In patients with heart failure low‐quality evidence showed a reduction in heart failure admission with NP‐guided treatment while low‐quality evidence showed uncertainty in the effect of NP‐guided treatment for all‐cause mortality, heart failure mortality, and all‐cause admission. Uncertainty in the effect was further shown by very low‐quality evidence for patient's quality of life. The evidence for adverse events and cost of treatment was low quality and we were unable to pool results.

## Plain language summary

**B‐type natriuretic peptide‐guided treatment for heart failure patients**

**Review question**

We aimed to discover whether using B‐type natriuretic‐guided treatment or a health plan alone is more effective for managing patients with heart failure.

**Background**

Heart failure is a complex condition that occurs when the heart does not pump blood effectively enough to meet the needs of the body. It is caused by a range of diseases that impair the structure and function of the heart and may result in breathlessness, fatigue and fluid retention. People with heart failure are frequently users of general practice and hospitals, particularly as inpatients. Furthermore, they have reduced life expectancy, although medicines and other treatments can improve the chance of survival.

B‐type natriuretic peptide (NP) is a substance produced in the heart. The measurement of NP can be used to indicate the condition of the heart. For some time, NP has been used for diagnosing heart failure and predicting what is likely to happen. We wanted to discover if NP may also offer a way to manage and make the best use of medicines.

**Study selection and characteristics**

We carried out a review of all studies and the evidence is current to 15 March 2016. We found 18 studies of NP‐guided treatment in which 3660 patients with heart failure took part. Patients were between 62 to 80 years old at the start of the studies. The duration of each study ranged from one to 54 months.

Eight out of the 18 studies were part or fully funded by pharmaceutical companies, one was funded by a national research body, five were partially funded either by national research grants, lotteries, hospital funds and/or pharmaceutical companies and four studies did not report the funding source.

**Key results**

The evidence was unclear as to whether number of deaths from any cause varied between patients with heart failure using NP‐guided treatment compared with those using a health plan alone. Nor was it clear as to whether there were less deaths when the results were separated into patients older or younger than 75 years old (age results only included three studies). Furthermore, we found that the evidence was unclear whether the number of deaths from heart failure alone varied between the NP‐guided treatment or health plan alone groups.

We found that hospital admission due to heart failure may be reduced in the patients using NP‐guided treatment compared with a health plan alone. Based on these results we would expect that out of 1000 patients with heart failure who are guided by a health plan alone, 377 would experience an admission to hospital due to heart failure. Whereas, between 230 and 301 patients would experience an admission to hospital due to heart failure if they received NP‐guided treatment. However, the evidence was unclear as to whether the numbers of hospital admission from any cause were affected.

There was limited information about either harms to patients, or the cost of the treatment. It was not possible to combine the results from these studies for these outcomes. However, four of the six studies commented that they found no difference in harms or less difference in harms between the patients using NP‐guided treatment compared with a health plan alone, the other two studies did not comment. Four studies reported results on costs, three of these reported there may be lower costs in the NP‐guided treatment groups compared with health plan groups. Lower costs appeared to be due to less cost for hospital stays. However, one study reported that NP‐guided treatment was unlikely to be cost‐effective.

The evidence was unclear as to if a benefit was shown in the replies to quality‐of‐life surveys when comparing between NP‐guided treatment and health plan only groups.

**Quality of evidence**

Overall evidence for death from all causes, from heart failure alone and for hospital admission was of low quality. For harm to patients and cost outcomes the quality of evidence was low, whilst evidence for patients' quality of life surveys was very low. For all outcomes there was little evidence due to the way the studies were conducted. In addition, for harm to patients and cost of treatment there were differences in the type of information available.

## Summary of findings

**Summary of findings for the main comparison:** Does treatment guided by serial BNP or NT‐proBNP monitoring improve outcomes compared to treatment guided by clinical assessment alone?

**Does treatment guided by serial BNP or NT‐proBNP monitoring improve outcomes compared to treatment guided by clinical assessment alone?**						
**Patient or population:** patients with heart failure **Settings:** in‐hospital and out‐of‐hospital **Intervention:** serial BNP or NT‐proBNP‐guided treatment **Comparison:** no BNP or NT‐proBNP‐guided treament^1^						
**Outcomes**	Illustrative comparative risks* (95% CI)		**Relative effect (95% CI)**	**No of Participants (studies)**	**Quality of the evidence (GRADE)**	**Comments**
	**Assumed risk**	**Corresponding risk**				
	** No BNP or NT‐proBNP‐guided treatment**	** Serial BNP or NT‐proBNP‐guided treatment**				
**All‐cause mortality** Follow‐up: 3 to 54 months	**218 per 1000**	**190 per 1000** (166 to 220)	**RR 0.87** (0.76 to 1.01)	3169 (15 studies)	⊕⊕⊝⊝ **low^2^**^,3^	16 studies reported on all‐cause mortality (n = 3292), but only 15 studies are included in the meta‐analysis (n = 3169). For one study data could not be extracted or obtained in a format useable in the review. Funnel plot analysis suggests possible lack of small studies (beneficial control effect). Insufficient to justify downgrading the quality of evidence.
**Heart failure mortality** Follow‐up: 6 ‐ 24 months	**91 per 1000**	**76 per 1000** (49 to 118)	**RR 0.84** (0.54 to 1.30)	853 (6 studies)	⊕⊕⊝⊝ **low^3,4^**	
**Heart failure admissions** Follow‐up: 12 ‐ 54 months	**377 per 1000^2^**	**264 per 1000** (230 to 301)	**RR 0.70** (0.61 to 0.80)	1928 (10 studies)	⊕⊕⊝⊝ **low^4,5^**	
**All‐cause admissions** Follow‐up: 3 ‐ 54 months	**573 per 1000^2^**	**533 per 1000** (481 to 590)	**RR 0.93** (0.84 to 1.03)	1142 (6 studies)	⊕⊕⊝⊝ **low^3,4^**	
**Adverse events** Follow‐up: 9 ‐ 24 months	See comment	See comment	Not estimable	1144 (6 studies)	⊕⊕⊝⊝ **low^4,6^**	3/6 studies commented on the difference between the intervention and control groups: no significant difference in one and two favoured the intervention group
**Cost** Follow‐up: 12 ‐ 18 months	See comment	See comment	Not estimable	1051 (4 studies)	⊕⊕⊝⊝ **low^4,7^**	3/4 studies suggested reduced cost in the intervention groups. One study suggested NP‐guided treatment was unlikely to be cost‐effective.
**Quality of life** Scale from: 0 to 105. Follow‐up: 3 ‐ 54 months	The mean quality of life ranged across control groups from **23 ‐ 34.5 scores**	The mean quality of life in the intervention groups was **0.03 lower** (1.18 lower to 1.13 higher)		1812 (8 studies)	⊕⊝⊝⊝ **very low^4,8,9^**	Lower score indicates better quality of life
*The basis for the **assumed risk** (e.g. the median control group risk across studies) is provided in footnotes. The **corresponding risk** (and its 95% confidence interval) is based on the assumed risk in the comparison group and the **relative effect** of the intervention (and its 95% CI). **CI:** Confidence interval; **RR:** Risk ratio;						
GRADE Working Group grades of evidence **High quality:** Further research is very unlikely to change our confidence in the estimate of effect. **Moderate quality:** Further research is likely to have an important impact on our confidence in the estimate of effect and may change the estimate. **Low quality:** Further research is very likely to have an important impact on our confidence in the estimate of effect and is likely to change the estimate. **Very low quality:** We are very uncertain about the estimate.						

^1^ The comparisons (controls) fell into two groups: same as the intervention without BNP or NT‐proBNP measures or usual care ^2^ Allocation concealment was unclear in half of the studies. In two thirds of studies one or both of participants and personnel were not blinded to allocated interventions ^3^ For all studies (bar one study for all‐cause mortality outcome) the point estimates and confidence intervals include the line of no effect. For all studies (bar two for all‐cause admissions outcome) the point estimates and confidence intervals cross the threshold of appreciable benefit or harm. ^4^ 66% or more of included studies did not blind participants and/or personnel ^5^ Heterogeneity substantial (I^2^: 60%, P value: 0.004) ^6^ Results for adverse events were not consistently reported since data were either first event or multiple events per individual. ^7^ The outcome measure differed for each study ^8^ Heterogenity substantial (I^2^: 75%, P value: 0.0002) ^9^ 95% confidence intervals are greater than 0.5 in either direction

## Background

### Description of the condition

Heart failure is a condition in which the heart does not pump enough blood to meet all the needs of the body. It is caused by dysfunction of the heart due to muscle damage (systolic or diastolic dysfunction), valvular dysfunction, arrhythmias or other rare causes (NICE 2014). Clinically, it is a syndrome in which patients have typical symptoms (e.g. breathlessness, ankle swelling, and fatigue) and signs (e.g. elevated jugular venous pressure, pulmonary crackles, and displaced apex beat).The diagnosis can be difficult as many of the symptoms of heart failure are non‐discriminating so the demonstration of an underlying cardiac cause is central to the diagnosis. Identification of the underlying cardiac problem is also crucial for therapeutic reasons, as the precise pathology determines the specific treatment used (e.g. valve surgery for valvular disease, specific pharmacological therapy for left ventricular systolic dysfunction, etc.) (McMurray 2012).

Heart failure due to left ventricular systolic dysfunction (LVSD) is caused by impaired left ventricular contraction, and is usually characterised by a reduced left ventricular ejection fraction (LVEF). Heart failure with preserved ejection fraction (HFPEF) is usually associated with impaired left ventricular relaxation, rather than left ventricular contraction, and is characterised by a normal or preserved left ventricular ejection fraction (NICE 2010).

Approximately 1% to 2% of the adult population in developed countries has heart failure, with the prevalence rising to ≥10% among persons 70 years of age or older (McMurray 2012). The prevalence is expected to rise in future as a result of an ageing population, improved survival of people with ischaemic heart disease and more effective treatments for heart failure (Owan 2006).

Heart failure has a poor prognosis: 30% to 40% of patients diagnosed with heart failure die within a year – but thereafter the mortality is less than 10% per year. There is evidence of a trend of improved prognosis in the past 10 years. The six‐month mortality rate decreased from 26% in 1995 to 14% in 2005. Within the NHS, heart failure accounts for a total of 1 million inpatient bed‑days – 2% of all NHS inpatient bed‐days – and 5% of all emergency medical admissions to hospital. Hospital admissions because of heart failure are projected to rise by 50% over the next 25 years, largely as a result of the ageing population. This is despite a progressive decline of the age‐adjusted hospitalisation rate at 1% to 1.5% per annum since 1992/1993 (NICE 2010).

### Description of the intervention

All patients with chronic heart failure require monitoring, which should include a detailed clinical assessment and a review of medication, including the need for titration and optimisation in line with guidelines and to pick up possible side effects. The pharmacological treatment options for patients with LVSD (New York Heart Association (NYHA) functional class II–IV) include diuretics, angiotensin‐converting enzyme (ACE) inhibitors (angiotensin receptor blockers if ACE inhibitors are not tolerated), beta‐blockers and mineralocorticoid receptor antagonists (MRA).

The frequency of monitoring depends on the clinical status and stability of the patient. The monitoring interval should be short (days to two weeks) if the clinical condition or medication has changed, but is required at least six‐monthly for stable patients with proven heart failure.

The intervention requires monitoring of B‐type natriuretic peptide concentrations to guide treatment of heart failure with the aim of enhancing the management of individual patients. B‐type natriuretic peptide, along with NT‐proBNP, is a natriuretic peptide secreted when the heart stretches. B‐type natriuretic peptide has a shorter half life of 20 minutes compared to the one to two hours for NT‐proBNP, and both can be increased in patients with systolic or diastolic dysfunction (Atisha 2004). Both biomarkers have demonstrated diagnostic and prognostic utility in heart failure (Clerico 2007; Doust 2005; McMurray 2012NICE 2014). Monitoring NP concentration provides feedback to the physician about intravascular volume status, which can be used in combination with the patient's clinical condition to facilitate treatment decisions.

### How the intervention might work

BNP and NT‐proBNP (collectively referred to as NP) are biomarkers for heart failure which have been demonstrated to have diagnostic and prognostic utility (Clerico 2007; Doust 2005, McMurray 2012, NICE 2014). The precursor, preproBNP is cleaved to proBNP within the cardiomyocyte and stored in secretory granules; proBNP is cleaved to NT proBNP and BNP upon secretion into the bloodstream in response to an increase in intracardiac volume (Chen 2010; Ichiki 2013). Monitoring NP concentrations provides feedback to the physician about intravascular volume status, which can be used in combination with the patient's clinical condition to facilitate treatment decisions.

### Why it is important to do this review

To date, five out of seven systematic reviews with meta‐analyses have demonstrated that NP‐guided treatment reduces all‐cause mortality in patients with congestive heart failure compared with usual clinical care (Felker 2009; Li 2013; Li 2014; Porapakkham 2010; Savarese 2013), especially in patients younger than 75 years of age (Porapakkham 2010). In 2014, Troughton et al (Troughton 2014) published an individual patient meta‐analysis and Xin et al (Xin 2015) published a meta‐analysis which contradicted this finding for all‐cause mortality in all patients. Uncertainty remains as to whether the monitoring of NP may lead to more harm than benefit compared with usual care. No other review has examined heart failure mortality. Fewer reviews have examined whether NP‐guided treatment increases or reduces heart failure admissions ( Li 2013;Li 2014; Savarese 2013, Troughton 2014; Xin 2015) or all‐cause hospital admissions (Porapakkham 2010; Savarese 2013; Troughton 2014; Xin 2015) .

Two reviews have examined adverse events (Li 2014; Xin 2015) and no review has examined the cost of treatment. Only Xin 2015 has examined quality of life data.

Monitoring with NP is recommended by NICE only for some patients by a specialist after hospital admission or when up‐titration of medication is problematic (NICE 2010). It is not recommended by the European Society of Cardiology (ESC) guideline (McMurray 2012) due to uncertainty about whether it is a more effective approach than simply optimising treatment (combinations and doses of drugs, devices) according to guidelines.

In this review, we examined the seven outcomes described above and in addition included heart failure mortality, which has not been examined previously. In addition, we aimed to evaluate whether factors such as age, gender, severity of symptoms or stage of heart failure, and context of care (community or hospital) predicted whether a patient will benefit from NP monitoring, furthermore whether monitoring leads to a greater change in NP. However, only one of these pre‐specified subgroup analyses was possible due to lack of data or inconsistency in reporting for these factors. Four further subgroup analyses were considered post‐hoc: baseline LVEF, duration of follow‐up, type of control, and type of biomarker.

## Objectives

Our objectives are:

1. to assess whether treatment guided* by serial BNP or NT‐proBNP (collectively referred to as NP) monitoring improves outcomes compared with treatment guided by clinical assessment alone;
2. to assess the extent to which improved outcomes are explained by up‐titration of medication and/or reductions in BNP levels; and
3. to determine which groups of patients benefit most from monitoring in terms of their age, gender, severity of symptoms or stage of heart failure (with the use of the NYHA classification), and baseline NP.

*Treatment guided within this review refers to lifestyle and medication changes for the management of heart failure (i.e. no device therapy or transplantation).

## Methods

### Criteria for considering studies for this review

#### Types of studies

All randomised controlled trials of BNP‐ or NT‐proBNP‐guided (collectively NP‐guided) treatment of heart failure, in both in‐hospital and out‐of‐hospital settings, reporting a clinical outcome. No restriction on length of follow‐up.

#### Types of participants

All patients 18 years and older who are being treated for heart failure.

#### Types of interventions

Comparison of treatment guided by NP levels versus treatment guided by clinical assessment alone.

#### Types of outcome measures

##### Primary outcomes

The primary outcome was all‐cause mortality. 

##### Secondary outcomes

The secondary outcomes were as follows:

1. heart failure mortality;
2. heart failure admission;
3. all‐cause admission;
4. adverse events;
5. cost; and
6. quality of life.

### Search methods for identification of studies

#### Electronic searches

We searched the following databases on 15 March 2016:

1. Cochrane Central Register of Controlled Trials (CENTRAL) in the Cochrane Library (2016, Issue 2),
2. MEDLINE (OVID, 1946 to 15 March 2016),
3. Embase (OVID, 1974 to 14 March 2016),
4. Database of Abstracts of Reviews of Effects (DARE) in the Cochrane Library (2015, Issue 2),
5. NHS Economic Evaluation Database (NHSEED) in the Cochrane Library (2015, Issue 2), and
6. Science Citation Index Expanded and the Conference Proceedings Citation Index on Web of Science (Thomson Reuters, 1945 to 15 March 2016).

Search filters limiting searches to randomised controlled trials were applied to MEDLINE and Embase (Lefebvre 2011). See Appendix 1 for the detailed search strategies. We applied no date or language restrictions.

#### Searching other resources

We contacted authors of relevant studies, performed citation searches and reviewed references of all full text papers retrieved. We also contacted experts in the field when relevant. We identified any ongoing trials that were registered with the World Health Organization International Clinical Trials Registry Platform (http://apps.who.int/trialsearch/) and ClinicalTrials.gov (http://clinicaltrials.gov) on 15 March 2016.

### Data collection and analysis

#### Selection of studies

We screened the title and abstract of articles obtained from the search results (LW/JM/NP/CB) for studies that met the inclusion criteria as well as any articles in which there was uncertainty. For each article, two review authors (LW/JM/NP/CB) independently reviewed the studies for final inclusion/exclusion. In cases where it was still unclear, we contacted the study authors for clarification. We resolved disagreements by consensus or third‐party adjudication (CH/RP).

#### Data extraction and management

We used data abstraction forms specifically designed for this review to abstract data on participants, interventions, and outcomes. For each study two review authors (LW/JM/NP) extracted trial results independently. We resolved differences between authors' results by discussion and, when necessary, in consultation with a third review author (CH/RP). Where data were insufficiently reported in the published paper, we wrote to the original authors for clarification and further information.

#### Assessment of risk of bias in included studies

Three review authors (LW/JM/NP) independently assessed methodological information, two for each study. The specific components assessed included allocation concealment, random sequence generation, blinding of participants, personnel, and outcome assessment, incomplete outcome data, selective reporting and source of funding. We reported our judgement for each component using Cochrane's tool for 'Risk of bias' assessment (Higgins 2011).

#### Unit of analysis issues

No included studies had nonstandard designs such as cross‐over or cluster‐randomised. If a study compared more than one type of control group then the intervention group data were split equally between the control groups for both outcome events and sample size.

For continuous outcomes, if the study provided data as medians and interquartile ranges then medians were assumed to equate to the mean and the interquartile ranges were converted to standard deviations by dividing the difference between the two values divided by 1.35 (approximate relationship between the two assuming a normal distribution). The mean difference and standard deviation were calculated assuming a correlation of 0.5 (Higgins 2011).

#### Dealing with missing data

Where data were insufficiently reported in the published paper, we wrote to the original authors for clarification and further information. We analysed only the available data and discussed the impact of the missing data on our findings.

#### Assessment of heterogeneity

Where we pooled data, we used the I^2^ statistic to quantify the level of statistical heterogeneity (Higgins 2011) .

#### Assessment of reporting biases

We assessed publication bias by the use of funnel plots where there were sufficient studies, and reasons for asymmetry were considered if it was noted. We addressed other potential reporting biases in the Discussion.

#### Data synthesis

Where appropriate, we pooled data from all the studies using the analysis software in Review Manager (RevMan) version 5.3. For dichotomous outcomes, we combined data using a fixed‐effect model with the Mantzel‐Haenzel method to determine a summary estimate of the risk ratio (RR) with 95% confidence intervals (CI). For continuous outcomes, we used a fixed‐effect model with the inverse variance method to produce a mean difference (MD) with 95% CI for the summary estimate. Where substantial heterogeneity (I^2^ ≥ 50%) was present, we considered potential explanations and where applicable used a random‐effects model to test the robustness of the findings and also considered not combining the results and presenting a descriptive analysis.

#### Subgroup analysis and investigation of heterogeneity

We considered subgroup analyses for the following:

1. age;
2. severity of heart failure (New York Heart Association (NYHA) classification);
3. baseline NP;
4. target NP;
5. achieved NP decrease (as a percentage of baseline);
6. patients treated in the community compared with those treated in secondary care;
7. gender.

Post hoc subgroup analyses were subsequently considered for:

1. baseline left ventricular ejection fraction;
2. duration of follow‐up (≤ one year, one to two years, > two years);
3. control type;
4. biomarker (BNP, NT‐proBNP).

#### Sensitivity analysis

We incorporated the results of the 'Risk of bias' assessment into our interpretation of the results by performing sensitivity analyses in which we excluded studies with the highest level of or unclear bias and included low risk of bias studies only.

## Results

### Description of studies

#### Results of the search

The search identified 3394 references. Once duplicates were removed, the titles and abstracts of the remaining 3379 references were screened using our inclusion /exclusion criteria and 3044 removed as not relevant to the review. Full texts were examined for the remaining 335 references and from these 18 studies were included in this review (see Figure 1). Full details of all the studies are given in the Characteristics of included studies, Table 2, Table 3, Characteristics of excluded studies, and Characteristics of ongoing studies. Each study is identified by the name of the first author and year of publication of the main results paper (Study ID). Additional references are listed together with this main publication under the study ID.

**1:**
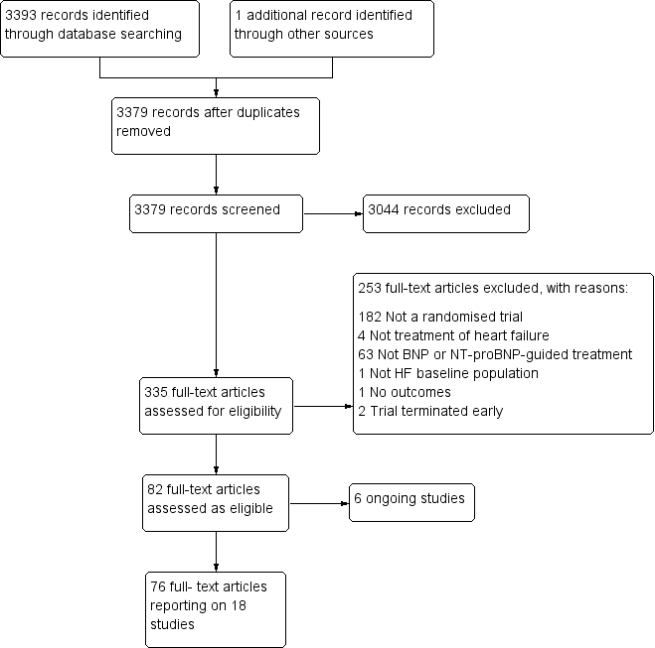
Study flow diagram.

**1:** Subgroup data: Setting, NYHA, LVEF (considered post‐hoc)

Study	Participants treated in community or secondary care	Baseline NYHA classification (stages I ‐ IV)				Baseline left ventricular ejection fraction (LVEF, %)		
		Study inclusion criteria	Intervention group	Control group	Comment in text	Study inclusion criteria	Intervention group (mean, SD unless stated)	Control group (mean, SD unless stated)
Anguita 2010	Hospital	Stage ≥ III	Stage III 73%, IV 27%	Stage III 63%, IV 37%		Not inclusion criterion	44 (18)	46 (18)
Beck‐da‐Silva 2005	Hospital (outpatient)	Stages II ‐ III	2.6 ± 0.7 (mean, SD)	2.4 ± 0.6 (mean, SD)		<40%	23.8 ± 8.8	20.9 ± 9.2
Berger 2010	Hospital & community	Stages III ‐ IV	Not stated	Not stated		<40%	NS	NS
Eurlings 2010	Hospital	Not inclusion criterion	Stage I = 11.5%, II = 64.9%, III = 23.6%	stage I = 9.9%, II = 70.8%, III = 19.3%		Not inclusion criterion	34.9 ± 13.7	36.7 ± 14.8
Januzzi 2011	Hospital	Stages II ‐ IV	Stage II or III = 85.5%	Stage II or III = 84.2%		≤ 40%	28 ± 8.7	25.9 ± 8.3
Jourdain 2007	Hospital (outpatient)	Stages II ‐ III	2.29 ±0.6 (mean, SD)	2.21 ± 0.62 (mean, SD)		<45%	29.9 ± 7.7	31.8 ± 8.4
Karlstrom 2011	Hospital	Stages II ‐ IV	Stage II = 32%, III = 52%, IV = 15%	Stage II = 27%, III = 59%, IV = 14%		<40%	<30% = 57%	<30% = 58%
Krupicka 2010	Hospital	Stages III ‐ IV	2.1 (0.3) (mean, SD)	2.1 (0.3) (mean, SD)		≤ 45%	36.1% (7.2)	32.3% (9.6)
Lainchbury 2010	Hospital & community	Not inclusion criterion	NT‐proBNP group: stage I 12%, II 68%, III 18%, IV 2%	Clinically‐guided group: Stage I 7%, II 66%, III 25%, IV 2%; Usual care: stage I 7%, II 67%, III 25%, IV 1%		Not inclusion criterion though deliberated included patients with preserved LVEF	40 ±15	CG = 39 ± 15, UC = 37 ± 15
Li 2015	Hospital	Stages III ‐ IV	NS	NS		Not inclusion criterion	30 ± 8.1	28 ± 7.9
Maeder 2013	Hospital (outpatient)	Stages ≤ II	49 (83) ≥ III (median, IQR)	53 (83) ≥ III (median, IQR)	'symptoms improved similarly' (at 6 months)	> 45%	56 ± 6	56 ± 7
Persson 2010	Community	Stage II ‐ IV	Stage II 62%, III 38%	Stage II 61%, III 39%	'Improvements in NYHA class and dyspnoea symptoms were seen in both allocation groups, but with no significant differences between the groups'	<50%	31 (9)	33 (7)
Pfisterer 2009	Hospital (outpatient)	Stages ≤ II	186 ≥ III (n)	185 ≥ III (n)		≤ 45%	29.8 (7.7)	29.7 (7.9)
Schou 2013	Hospital	Not inclusion criterion	Stage I ‐ II 86 %	Stage I ‐ II 85 %		<45%	30 (14‐45) median (range)	30 (15‐45) median (range)
Shah 2011	Hospital	Stage III ‐ IV	Authors have no data for baseline NYHA	Authors have no data for baseline NYHA		<35%	20 (15‐25) median (range)	20 (15‐25) median (range)
Shochat 2012	Hospital	Not stated	2.53 (mean)	2.34 (mean)		Not inclusion criterion	23 (6)	23 (7)
Skvortsov 2015	Hospital (outpatient)	Stage III ‐ IV	Stage III 23%, IV 76%	Stage III 26%, IV 74%	At hospital admission	<40%	29.2 (6.1)	29.4 (6.1)
Troughton 2000	Hospital	Stages II ‐ IV	Stage II 72%, overall 2.3 (mean)	Stage II 67%, overall 2.3 (mean)		<40%	28	26

**2:** Subgroup data: Biomarker target, baseline and change from baseline measurements

Study	Target BNP/NT‐proBNP (pg/mL, unless stated)	Baseline BNP or NT‐proBNP measurement (units in pg/mL and given as mean (SD), unless stated)					BNP/NT‐proBNP drop (as % of baseline) (units in pg/mL and given as mean (SD), unless stated)
		Biomarker	Study inclusion criteria	Intervention group	Control group	Comment in text	
Anguita 2010	100	BNP	No inclusion threshold	57 (77)	65 (97)		No percentage drop reported. BNP at 18 months follow‐up: BNP‐guided group 14 (20); control group 111 (71)
Beck‐da‐Silva 2005	No target set/stated	BNP	No inclusion threshold	502.3 (411.3)	701.6 (409.9)		No percentage drop reported. BNP at follow‐up: control arm 626.8 (325.8); BNP arm 477.8 (406.9)
Berger 2010	< 2200 NT = proBNP (reported in IPD analysis by Troughton 2014)	NT‐proBNP	No inclusion threshold	2216 (355‐9649) mean (95% CI)	Multidisplinary care 2469 (355 ‐18487; Usual care 2359 (355 ‐15603) mean (95% CI)		No percentage drop reported. NT ‐proBNP change from baseline to FU graphically shown in Berger 2010 (Figure 2). Decrease in NT‐proBNP more apparent in NT‐proBNP‐guided group than multidisplinary group. No decrease in usual care group
Eurlings 2010	Set individually for each participant as the lowest level at discharge or at 2 weeks follow‐up	NT‐proBNP	NT‐proBNP levels at admission: minimum 1,700 pg/ml. Additionally NT‐proBNP levels during hospitalisation, defined as a decrease of more than 10%, with a drop in NT‐proBNP levels of at least 850 pg/ml, from admission to discharge.	2961 (1383 ‐ 5144) median (IQR)	2936 (1291‐5525) median (IQR)	Outcome data available by subgroup baseline BNP (above or below discharge NT‐proBNP 2950 pg/ml)	No percentage drop reported. Median (IQR) at 12 months follow‐up: NT‐proBNP‐guided group ‐432 (‐1392 to 297); Clincially‐guided group ‐572 (‐1329 to 434).
Januzzi 2011	≤ 1000	NT‐proBNP	No inclusion threshold	2344 (median)	1946 (median)		No percentage drop reported. Median NT‐proBNP at follow‐up: Standard care group 1844 (P = 0.61 follow‐up vs baseline); NT‐proBNP‐guided group 1125 (P = 0.01 vs baseline)
Jourdain 2007	< 100	BNP	No inclusion threshold	352 (260) mean (SD)	Not measured		No percentage drop reported. BNP‐guided group only shown graphically in Jourdain 2007 (figure 5): mean BNP level drops over time and % of patients achieving target increases.
Karlstrom 2011	<150 ng/L in patients under 75; <300 ng/L in patients over 75 yrs	BNP	No inclusion threshold	808.2 (676.1) ng/L, mean (SD)	898.9 (915.3 ng/L, mean (SD)		No percentage drop reported. BNP at follow‐up: control group 457 (603), BNP‐guided group 403 (468)
Krupicka 2010	<100	BNP	No inclusion threshold	704 (228‐2852) median (range)	633 (276‐3756) median (range)		No percentage drop reported. In the BNP group 90% of patients manage to reduce BNP to <400 pg/mL; of this 90%, 2/3 of patients to achieve <100 pg/mL. Email from author "We do not have BNP values of the Clinical group at the end of follow‐up. Median BNP value after 6 months in BNP group was 235pg/ml. (At hospital discharge 704pg/ml; after 1 month 328.5pg/ml; after 3 months 253pg/ml)."
Lainchbury 2010	< 150 µmol/L	NT‐proBNP	No inclusion threshold	2012 (516‐10233) median (IQR)	Clinically‐guided group: 1996 (425‐6588); Usual care: 2012 (425‐10571) median (IQR)		No percentage drop reported. No follow‐up data. Comment in text 'Plasma NT‐proBNP levels fell similarly within 6 months of randomisation in both the NT‐proBNP and CG groups (by 20% and 23%, respectively; P 0.001)'.
Li 2015	50% of basal level or < 300	BNP	No inclusion threshold	1167.8 (219.9) mean (SD)	1145.8 (224.9) mean (SD)		No percentage drop reported. Change in BNP level shown in Figure 2 (Li 2015). 'BNP value decreased dramatically over the duration of medication, but there was no difference between the two groups.'
Maeder 2013	< 400 in patients younger than 75 years; < 800 in patients aged 75 years or older	NT‐proBNP	N‐terminal BNP level of 400 pg/mL or higher in patients younger than 75 years and a level of 800 pg/mL or higher in patients aged 75 years or older	2210 (1514‐4081) ng/L, median (IQR)	2191 (1478‐4890) ng/L, median (IQR)		Maeder 2013 reports: 'NT‐proBNP was reduced similarly in patients allocated to NT‐proBNP‐guided or symptom‐guided management. The proportion of patients with NT‐proBNP below the target was low throughout the study period and did not significantly differ between groups (Figure 2C) although it tended to be lower in the NT‐proBNP‐guided group.
Persson 2010	At least a 50% reduction from baseline NT‐proBNP	NT‐proBNP	Elevated NT‐proBNP levels (males > 800 ng/L, females > 1000 ng/L)	2661 (2.1) ng/L, geometric mean(coefficient of variation, %)	2429 (2.1) ng/L, geometric mean(coefficient of variation, %)		No percentage drop reported. Geometric Mean (SD) at follow‐up: NT‐proBNP‐guided group ‐ 301 ng/L to 2360 ng/L; control group ‐362 ng/L to 2067 ng/L. Comment in text 'similar modest decrease ( 10%) in NT‐proBNP from baseline to end‐of study was observed in both groups……NT‐proBNP levels were reduced by .50% in 24 (19%) and 27 (22%), of patients with and without NT‐proBNP‐guided treatment, respectively'.
Pfisterer 2009	< 400 in patients younger than 75 years; < 800 in patients aged 75 years or older	NT‐proBNP	N‐terminal BNP level of 400 pg/mL or higher in patients younger than 75 years and a level of 800 pg/mL or higher in patients aged 75 years or older	3998 (2075‐7220) median (IQR)	4657 (2455‐7520) median (IQR)		No percentage drop reported. No follow‐up data. Pfisterer 2009 (figure 3b) graphically shows data for NT‐proBNP changes over 6 months (by age). Comment in text 'There were no significant differences between the 2 treatment groups by by N‐terminal BNP level (P=.06 vs P=.30).'
Schou 2013	No target set/stated	NT‐proBNP	NT‐proBNP ≥ 1000 pg/mL after up‐titration (i.e. at the randomisation visit)	1884 (1033‐10435) average statistic not stated)	2042 (1023‐9668) average statistic not stated		No percentage drop reported. Change in NT‐proBNP during follow‐up: NT‐proBNP‐guided group ‐129 (‐722 to 674) median (IQR); Clinically managed group ‐26 (‐681 to 751) median (IQR). Comment in text: 'Patients in whom NT‐proBNP increased ≤ 30% during the follow up period had a higher frequency of admission (69% vs. 47%, P = 0.002), a higher number of admission days (median) (14 days vs. 5 days, P= 0.003), a higher number of admissions (median) (2 vs. 1, P = 0.009), a lower quality of life (mean difference) (6 points, P = 0.032), and a poorer functional class (37% vs. 18% in functional class III–IV, P = 0.001).'
Shah 2011	Discharge BNP	BNP	No inclusion threshold	453 (221‐1135) median (IQR)	440 (189 ‐981) median (IQR)		No percentage drop reported. Median (IQR) BNP at follow‐up: BNP‐guided group 412.5 (111,894); control (congestion score) group 471 (235.5, 1180)
Shochat 2012	No target set/stated	NT‐proBNP	Email from author confirmed 'NT‐ProBNP > 2000 at day of randomisation'	5868 (2532)	5820 (2434)		No percentage drop reported.
Skvortsov 2015	<1000 pg/mL or at least 50% reduction from baseline NT‐proBNP at discharge	NT‐proBNP	> 1400 pg/mL at hospital admission	3750 (2224‐ 6613) median (IQR)	2783.0 (2021.5‐ 4827.5) median (IQR)	At hospital discharge	At 6 months: NT‐proBNP‐guided group: 53% (Median drop (QR): 1585.5 (976.6, 2742.5)) Control group: 10.2% (median (IQR): 2189.0 (1954.0, 3688.5))
Troughton 2000	200 µmol/L	NT‐proBNP	No inclusion threshold	217 µmol/L, mean	251 µmol/l, mean		No percentage drop reported. At 6 months follow‐up: Nt‐proBNP‐guided group decreased by 79 pmol/L, mean; clinically‐guided group decreased by 3 pmol/L, mean (P = 0.16)

**4:**
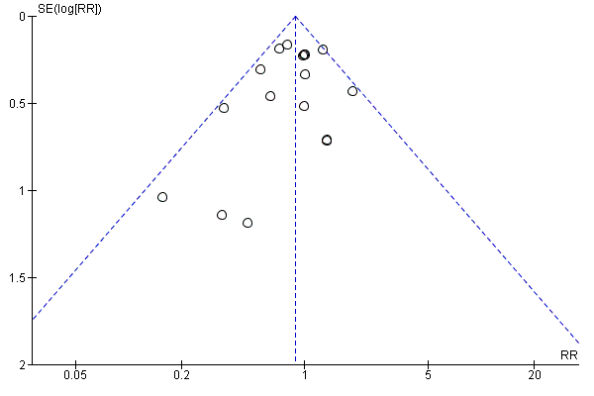
Funnel plot of comparison: NP‐guided versus no NP‐guided treatment for all‐cause mortality.

#### Included studies

The Characteristics of included studies, Table 2 and Table 3 provide details of each of the 18 included studies.

The earliest study was published in 2000 (Troughton 2000) and the latest in 2015 (Skvortsov 2015). For two of the studies, data were only available through conference abstracts and direct contact with the authors (Krupicka 2010; Shochat 2012).

Ten of the studies were completed in Europe (two in Sweden/Norway (Karlstrom 2011; Persson 2010), two in Switzerland/Germany (Maeder 2013; Pfisterer 2009), one in Austria (Berger 2010), France (Jourdain 2007), the Netherlands (Eurlings 2010), Spain (Anguita 2010), Denmark (Schou 2013). and the Czech Republic (Krupicka 2010)); three studies were completed in North America (two in the USA (Januzzi 2011; Shah 2011) and one in Canada (Beck‐da‐Silva 2005)); two were completed in New Zealand (Lainchbury 2010; Troughton 2000), one in Israel (Shochat 2012), one in Russia (Skvortsov 2015), and one in China (Li 2015).

Two of the 18 studies (Berger 2010; Lainchbury 2010) had three comparison arms comparing NP‐guided treatment both to clinical assessment and to usual care. For usual care there were no scheduled visits and the participants were managed in primary care. Studies recruited 3660 participants ranging from 41 to 499 participants per study. The average age of participants in all the studies ranged from 62 to 80 years old. Studies followed up participants from baseline to between one and 54 months.

Seven studies (Anguita 2010; Beck‐da‐Silva 2005; Jourdain 2007; Karlstrom 2011; Krupicka 2010; Li 2015; Shah 2011) used BNP as the biomarker; the remainder used NT‐proBNP. Only seven studies (Eurlings 2010; Maeder 2013; Persson 2010; Pfisterer 2009; Schou 2013; Shochat 2012; Skvortsov 2015) stated an NP level as an inclusion criterion. All studies set a NP target except for Beck‐da‐Silva 2005; Schou 2013 and Shochat 2012 who stated a change in NP level (See Table 3).

Two studies (Beck‐da‐Silva 2005; Li 2015), compared the effect of NP‐guided treatment with clinical assessment exclusively for the up‐titration of beta‐blockers. Beck‐da‐Silva 2005 changed the dose of bisoprolol, but all other drugs remained unchanged, during a three‐month follow‐up period. Li 2015 started and increased the dose of metoprolol succinate over one month; for these patients intravenous cardiotonic, vasodilator or diuretic was applied if signs or symptoms of heart failure were observed.

Beck‐da‐Silva 2005 was the only study to report an algorithm where medication (beta blocker) was decreased for patients whom the BNP measurement was increasing, but the clinical assessment was worse.

All, bar three studies (Eurlings 2010, Lainchbury 2010; Schou 2013), reported inclusion criteria for classifying participants according to the New York Heart Association (NYHA) functional classification. This classifies patients with heart disease into four stages based on limitations on physical activity, symptoms with ordinary physical activity and status at rest. Stage four indicating the highest severity of symptoms. At baseline, most studies grouped participants by NYHA stage and overall, the participants ranged between stages II and IV. Three studies reported baseline NYHA as percentages in each stage: for Eurlings 2010 and Lainchbury 2010, over 60% of participants were in class II and for Schou 2013 over 85% were in stages I to II.

Further classification was determined by percentage left ventricular ejection fraction (LVEF); 12 of the studies stated as an inclusion criterion a maximum level for percentage LVEF which ranged between < 35% to < 50%; five studies did not stipulate any inclusion level (Anguita 2010; Eurlings 2010; Lainchbury 2010; Li 2015; Shochat 2012); and Maeder 2013 was the only study to have participants solely with percentage > 45% LVEF or preserved LVEF. Although six of the studies did not stipulate an inclusion level percentage LVEF, Lainchbury 2010 was the only other study to state participants with preserved LVEF were not excluded. At baseline, Berger 2010 did not report LVEF percentage, Maeder 2013 reported all participants averaged 56% LVEF, Karlstrom 2011 reported 57% of participants were < 30% LVEF, whilst the remaining studies reported overall averages ranging from 20% to 46% LVEF.

Six studies (Felker 2014; Jourdain 2014; Metra 2012; Moe 2007; Saraya 2015; Steinen 2014) are classified as ongoing. Of these, four studies (Felker 2014; Jourdain 2014; Moe 2007; Steinen 2014) are currently recruiting or have just finished recruiting. Metra 2012 finished recruiting in August 2009 and is due to publish shortly. Saraya 2015 has been completed, but currently only published as a conference abstract. All six are listed in the Characteristics of ongoing studies.

#### Excluded studies

Thirty‐five references are included in the Characteristics of excluded studies tables where the title or abstract or both appeared to suggest a relevant study to this review. Of these 68% were excluded as the study was not a randomised control trial. Other reasons included not NP‐guided treatment (20%), trial terminated, not treatment for heart failure, or not a baseline heart failure population.

### Risk of bias in included studies

(See Figure 3 and Figure 4)

**2:**
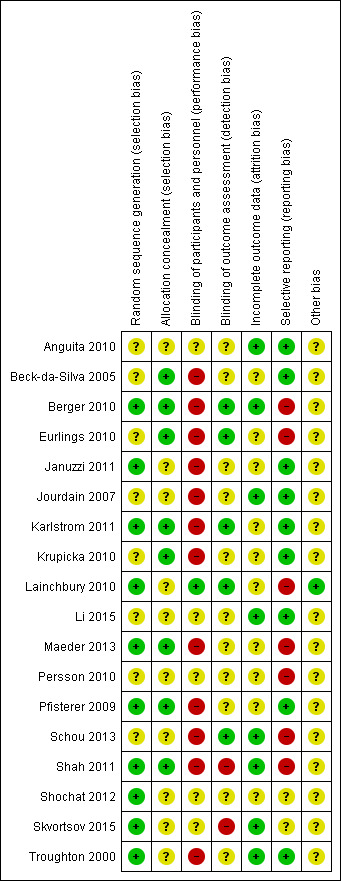
'Risk of bias' summary: review authors' judgements about methodological quality for each included study

**3:**
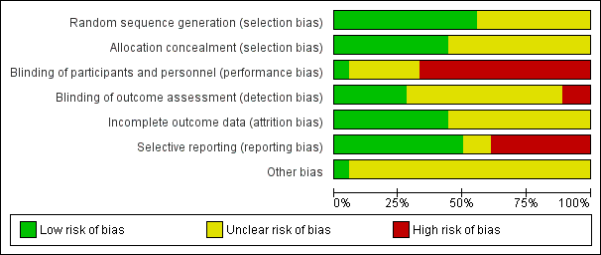
'Risk of bias' graph: review authors' judgements about methodological quality presented as percentages across all included studies.

#### Allocation

All studies clearly stated the study was randomised, but not all studies reported on how randomisation was completed or if allocation concealment was achieved. Five studies confirmed sequence generation and allocation concealment and methods were judged to be at low risk of bias (Berger 2010; Karlstrom 2011; Maeder 2013; Pfisterer 2009; Shah 2011). Januzzi 2011; Lainchbury 2010; Shochat 2012; Skvortsov 2015 and Troughton 2000 were low risk for sequence generation only and Beck‐da‐Silva 2005; Eurlings 2010 and Krupicka 2010 only for allocation concealment. The remaining studies were classified as unclear.

#### Blinding

Blinding of participants and study personnel was only judged to be low risk if both were blinded to the treatment allocation; only one study met this standard (Lainchbury 2010). Five studies did not report or it was unclear whether participants or personnel were blinded to treatment allocation (Anguita 2010; Li 2015; Persson 2010; Shochat 2012; Skvortsov 2015). In all the remaining studies one or more of these groups were not blinded. Blinding of outcome assessments was not achieved or not reported in the majority of studies; only five studies blinded outcome assessment (Berger 2010; Eurlings 2010; Karlstrom 2011; Lainchbury 2010; Schou 2013).

#### Incomplete outcome data

For the primary outcome, all‐cause mortality, eight studies (Anguita 2010; Berger 2010; Jourdain 2007; Li 2015; Schou 2013; Shah 2011; Skvortsov 2015; Troughton 2000) were judged to be low risk with regard to incomplete outcome data, in fact they all had no attrition except for Skvortsov 2015 where the numbers and reasons were fully reported. The remaining studies either did not report attrition, or the studies did confirm attrition with break down by intervention arm, but did not explain how missing data were handled. For those studies reporting dropouts, the overall attrition rates were no more than 23%.

All of the studies, bar four, completed intention‐to‐treat (ITT) analyses; Beck‐da‐Silva 2005 did not complete an ITT analysis, whilst Anguita 2010; Jourdain 2007 and Li 2015 did not report whether this method was used.

#### Selective reporting

Nine out of 18 studies reported on all stated outcomes and were considered low risk for reporting bias. Six studies have not yet reported on some secondary outcomes (Berger 2010 on heart failure mortality and all‐cause admission, Eurlings 2010 on all‐cause admission, Persson 2010 and Maeder 2013 on quality of life, Schou 2013 and Shah 2011 on treatment costs). Lainchbury 2010 partially reported quality of life data. Skvortsov 2015 is currently awaiting further publications. It was not possible to assess reporting bias for Shochat 2012 as data were provided from conference abstracts and direct contact with the author and any pre‐specified outcomes were not stated.

#### Other potential sources of bias

Eight of the studies were part or fully funded by pharmaceutical companies (Berger 2010; Januzzi 2011; Jourdain 2007; Krupicka 2010; Maeder 2013; Persson 2010; Pfisterer 2009; Shochat 2012). Five studies (Eurlings 2010; Karlstrom 2011; Schou 2013; Shah 2011; Troughton 2000) were partially funded by either national research grants, lotteries, hospital funds and/or pharmaceutical companies. Four studies did report funding sources (Anguita 2010, Beck‐da‐Silva 2005; Li 2015; Skvortsov 2015). These studies were judged to be of unclear risk of bias.

One study (Lainchbury 2010) was solely funded from a national research body and therefore considered at low risk of bias from the funding source.

### Effects of interventions

See: Table 1

(See Table 1)

#### All‐cause mortality

(See Analysis 1.1)

Sixteen studies (Anguita 2010; Beck‐da‐Silva 2005; Berger 2010; Eurlings 2010; Jourdain 2007; Karlstrom 2011; Krupicka 2010; Lainchbury 2010; Maeder 2013; Persson 2010; Pfisterer 2009; Schou 2013; Shah 2011; Shochat 2012; Skvortsov 2015; Troughton 2000) with 3292 participants recruited, reported results for all‐cause mortality. Follow‐up ranged from one month to four and a half years. However, data for Maeder 2013 was presented as survival curves and it was not possible to extract or obtain data for this study. Therefore meta‐analysis was only possible for the remaining 15 studies: During the follow‐up period, 265 (18%) participants died in the NP‐guided treatment groups compared to 368 (22%) in the control groups. When the data were pooled for all studies using a fixed‐effect model, the evidence favoured the guided treatment groups, but overall the evidence showed uncertainty (risk ratio (RR) 0.87, 95% confidence interval (CI) 0.76 to 1.01; patients = 3169; studies = 15; low quality of evidence). Heterogeneity was low (I^2^ = 16%).

The two studies that did not report results for all‐cause mortality were Januzzi 2011 and Li 2015.

#### Heart failure mortality

(See Analysis 1.2)

Only six studies (Jourdain 2007; Karlstrom 2011; Krupicka 2010; Li 2015; Skvortsov 2015; Troughton 2000) with 853 participants recruited reported results for heart failure mortality. In the NP‐guided treatment groups, 34 participants died and in the control groups 38 participants died due to heart failure, representing 8% and 9% respectively. Similar to all‐cause mortality, the pooled result, using a fixed‐effect model, favoured the intervention, but overall, the evidence showed uncertainty (RR 0.84, 95% CI 0.54 to 1.30; participants = 853; studies = 6; low quality of evidence). The heterogeneity was low (I^2^ = 21%).

#### Heart failure admission

(See Analysis 1.3)

Ten studies (Anguita 2010; Berger 2010; Januzzi 2011; Jourdain 2007; Karlstrom 2011; Krupicka 2010; Lainchbury 2010; Schou 2013; Skvortsov 2015; Troughton 2000) with 1928 participants reported on heart failure admission. Out of 858 participants, 219 (26%) experienced a heart failure event causing an admission in the NP‐guided treatment groups; this compared to 403 out of 1070 (38%) participants in the control groups. Overall, the pooled evidence for all 10 studies, with a fixed‐effect model, showed an effect favouring NP‐guided treatment (RR 0.70, 95% CI 0.61 to 0.80; participants = 1928; studies = 10; low quality of evidence). Heterogeneity was substantial (I^2^ = 60%). The robustness of this finding was tested by converting to a random‐effects model; the effect remained consistent (RR 0.67, 95% CI 0.53 to 0.84; participants = 1928; studies = 10; low quality of evidence).

#### All‐cause admission

(See Analysis 1.4)

Six studies (Beck‐da‐Silva 2005; Jourdain 2007; Karlstrom 2011; Schou 2013; Shah 2011; Troughton 2000) with 1142 participants recruited reported data for all‐cause admission. During the follow‐up, 304 (53%) participants experienced an event requiring admission in the NP‐guided treatment groups. This compared to 327 (57%) participants in the control groups. The pooled results for all studies, with a fixed‐effect model, favoured the intervention, but overall, the evidence showed uncertainty (RR 0.93, 95% CI 0.84 to 1.03; participants = 1142; studies = 6; low quality of evidence). No heterogeneity was identified (I^2^ = 0%). Lainchbury 2010 commented that no difference was seen between intervention and control groups for all‐cause admission, but the data were not provided.

#### Adverse events

(See Table 4)

**3:** Adverse event data

Study	Adverse events									
	Participants (N)			Missing participants (N)			Number of adverse events (definitions not consistent or not stated; not clear whether first event per participant or every event)			Additional data either from published articles or supplied by author
	Intervention group	Control group	Total	Intervention group	Control group	Total	Intervention group	Control group	Total	
Januzzi 2011	75	76	151	6	6	12	30	23	53	No significant differences between groups. No specific event showed a significant difference between groups Events in intervention group: Abdominal pain (1); acute renal failure (4); anaemia (1); atrial fibrillation (2); cough (2); diarrhoea (2); dizziness (5); fever (1); gastrointestinal bleeding (1); hyper/hypokalaemia (3); hypotension (4); respiratory infection (2); syncope(2) Events in control group: Abdominal pain (1); acute renal failure (3); anaemia (0); atrial fibrillation (5); cough (1); diarrhoea (1); dizziness (4); fever (1); gastrointestinal bleeding (1); hyper/hypokalaemia (1); hypotension (0); respiratory infection (4); syncope(1)
Krupicka 2010	26	26	52	0	0	0	7	0	7	Email from author 17.10.14 confirmed: Hyperkalaemia (n = 2); orthostatic hypotension (n = 2); bradycardia (n = 3)
Maeder 2013	59	64	123	12	12	24	Not reported	Not reported	66	Maeder 2013 reported: "58% of the patients in the NT‐proBNP‐guided and 50% in the symptom‐guided group had at least one SAE (p=0.32). SAE’s related to renal failure (14% versus 2%, p=0.01) were more common in the NT‐proBNP‐guided group, whereas hypotension tended to be less common (0% versus 8%, p=0.06)." No additional information
Persson 2010	126	124	250	8	7	15	42	39	81	No additional information provided
Pfisterer 2009	251	248	499	32	29	61	123	113	236	P = 0.47 Renal impairment: intervention group n = 4, control group n = 5 (P = 0.64) Hypotension: intervention group n = 6, control group n = 3 (P = 0.22) No other type of adverse event described. Adverse events ≥ 75 years old patients: intervention group 10.5% vs control group 5.5% (P = 0.12) Adverse events in < 75 years old patients: intervention group 3.7% vs. control group 4.9% (P = 0.74)
Troughton 2000	33	36	69	0	0	0	13	9	22	P = 0.32 No additional information provided

Six studies (Januzzi 2011; Krupicka 2010; Maeder 2013; Persson 2010; Pfisterer 2009; Troughton 2000) with 1144 participants reported number of adverse events during follow‐up. Maeder 2013 did not report the number of adverse events broken down by intervention group, only as a total for the study. For the remaining five studies, the NP‐guided treatment groups (511 participants) experienced 215 compared to 184 adverse events in the control groups (510 participants). Meta‐analysis was not viable for this outcome since it was possible to have multiple events per individual. Therefore, the results have been tabulated. Quality of evidence was low.

Nevertheless, three studies (Januzzi 2011; Pfisterer 2009; Troughton 2000) commented there was no difference between the NP‐guided treatment and control groups: Januzzi 2011 reported that there was no significant differences between the groups, whilst Pfisterer 2009 and Troughton 2000 reported P values greater than 0.05. Maeder 2013 reported the number of patients experiencing a serious adverse event did not differ between the groups. Two studies (Januzzi 2011; Krupicka 2010) reported a complete breakdown of the nature of the adverse events, whilst Pfisterer 2009 and Maeder 2013 only highlighted two areas (renal impairment and hypotension). For Maeder 2013, adverse events for renal failure were more frequent in the NP‐guided group, where as events were less frequent for hypotension compared to the control group. However, both Januzzi 2011 and Pfisterer 2009 confirmed no difference between the groups based on specific adverse events. Incomplete data meant it was not possible to comment on the most frequent types of adverse events.

#### Cost

Four studies (Berger 2010; Januzzi 2011; Maeder 2013; Pfisterer 2009) presented data on costs, two only as conference abstracts. It was not possible to pool results for these four studies because the outcome measure differed for each study. Pfisterer 2009 reported on total overall costs per intervention arm: $20,949 for the NT‐proBNP‐guided treatment group versus $23,928 in the symptom‐guided group (control). Generally, costs were comparable, the main difference occurred in the residency costs (staying in a nursing home or home for the elderly): $4157 in the NT‐proBNP‐guided treatment group versus $7564 in the symptom‐guided group.

Januzzi 2011 examined the mean costs in the duration of the study. Overall costs for the NT‐proBNP group totaled $35,262 ($451 per day) versus overall costs for the standard of care management (control) group of $42, 629 ($580 per day). Similar to Pfisterer 2009, the lower costs in the NT‐proBNP group was predominantly due to inpatient costs. Januzzi et al concluded that costs were reduced by approximately 20% in the NT‐proBNP‐guided treatment group over the 10‐month follow‐up period.

In Berger 2010 an economic analysis was completed for a subgroup of participants (n = 190) who had complete follow‐up data. This analysis suggested NP‐guided treatment was cost‐effective and cheaper than in the usual care control group (for the multidisciplinary care control group this was cost neutral).

In contrast to the above three studies Maeder 2013 reported NP‐guided therapy as unlikely to be cost‐effective. Overall costs being $38,876 per patient for the NP‐guided group compared to $21,419 per patient in the control group over 18 months.

Quality of evidence was low.

#### Quality of Life

(See Analysis 1.5)

Quality of life data were reported in eight studies ((Beck‐da‐Silva 2005; Eurlings 2010; Karlstrom 2011; Lainchbury 2010; Pfisterer 2009; Schou 2013; Skvortsov 2015; Troughton 2000) with 1812 participants recruited using the Minnesota Living with Heart Failure questionnaire. Lainchbury 2010 is only represented by one data set as data were only reported for the usual care control group. The pooled evidence for all studies, using a fixed‐effect model, marginally favoured NP‐guided groups, but overall, the evidence showed uncertainty (mean difference (MD) ‐0.03, 95% CI ‐1.18 to 1.13; very low quality of evidence). Heterogeneity was judged to be substantial (I^2^ = 75%).

Pfisterer 2009 also reported results for quality of life using the Short Form 12 and Duke Activity Status Index questionnaires; though not included due to incompatibility, both of these showed an improvement in both guided treatment and control groups with no differences in the degree of improvement.

In Karlstrom 2011, changes in quality of life for participants was measured using the Swedish and Norwegian Short Form Health Survey 36; 68% from the NP‐guided group and 74% from the control group completed the survey at both the start and end of the study. For these participants NP‐guided treatment did not improve quality of life compared to clinical assessment alone.

Participants in Persson 2010 completed the Kanas City Cardiomyopathy Questionnaire at baseline and follow‐up. This symptom score tool contains a quality of life element. In Persson 2010, the scores improved in both groups (+3.6 (SEM 1.65) in the NT‐proBNP group and +6.2 (SEM 1.66) in the control group). There was no differences between the groups (P = 0.28).

#### Subgroup analysis

Except for age, it was not possible to explore subgroups within the study populations. Data were reported for severity of heart failure, baseline NT‐proBNP, target NT‐proBNP, achieved NT‐proBNP/BNP drop and gender, but generally only as totals, in varying categories, or as averages, for intervention and control groups (Table 2, Table 3). Post hoc, consideration was given to subgrouping by left ventricular ejection fraction, (LVEF), but this too was not reported in an appropriate form (Table 2). All studies were completed under supervision of the hospital, except for Berger 2010 and Lainchbury 2010 where supervision was jointly in hospital and the community, and therefore subgroup analysis for this factor was not completed.

Subgroup analysis was only possible by age for three studies (Eurlings 2010; Lainchbury 2010; Shochat 2012) and only for the primary outcome of all‐cause mortality (see Analysis 3.1). From the three studies, including Lainchbury 2010 with two control groups, there were 830 participants. For this analysis, the age threshold was set as equal or greater than 75 years old versus under 75 years old, though the data from Eurlings 2010 are reported marginally different as greater than 74 versus equal to or less than 74 years old. When the data from these three studies were pooled, the evidence showed uncertainty for either age subgroup. However, whilst showing uncertainty for either age subgroup the results suggest that for participants equal to or greater than 75 years old, the effect favoured the control groups (RR 1.23, 95% CI 0.96 to 1.57; participants = 410; studies = 3) whilst for participants less than 75, the effect favoured the guided‐treatment groups ((RR 0.73, 95% CI 0.49 to 1.10; participants = 420; studies = 3) (Analysis 3.1).

Lainchbury 2010 further reported data by age for heart failure admission (=/< 75 years: RR 1.13, 95% CI 0.77 to 1.64; participants = 188; < 75 years: RR 0.73, 95% CI 0.45 to 1.17; participants = 177) (Analysis 3.2). The data followed a similar trend to the pooled data for age and all‐cause mortality.

Despite data not being available to pool, three further studies did comment on the age of participants in their results. Januzzi 2011 concluded for their study that 'no interaction between NT‐proBNP‐guided care and age was found (P = 0.11)'. Persson 2010 commented 'levels of NT‐proBNP tended to decrease more in patients younger than 75 years than in patients older than 75 years (change ‐2.4% ≥75 versus ‐20.3% <75 years, P = 0.06). Finally, Pfisterer 2009 reported that in the first six months the BNP levels decreased similarly for both guided treatment and control groups and were similar for participants under 75 and equal to or over 75 years of age. Though Pfisterer 2009 did state that "there was a significant interaction between treatment and age groups, i.e. patients aged ≥ 75 years in the NT‐proBNP group had a smaller relative benefit on NT‐proBNP levels (p = 0.04) and symptoms (p = 0.05) than younger patients". At eighteen months, the interaction between treatment and age was significant for mortality (P = 0.01, Cox regression adjusting for baseline characteristics) indicating that 'NT‐proBNP‐guided treatment differed significantly between younger and older patients'.

Post hoc subgroup analysis was carried out to explore whether data from two studies (Berger 2010; Lainchbury 2010) using usual care differed to all other studies using clinical assessment as the comparator to NP‐guided treatment (Analysis 2.1). This was only possible for two outcomes. For the primary outcome of all‐cause mortality, the evidence showed very little difference for either subgroup (usual care RR 0.79, 95% CI 0.56 to 1.13; participants = 319; studies =2; clinical assessment RR 0.89, 95% CI 0.76 to 1.04; participants = 2850; studies = 15) to each other or compared to the overall pooled result (RR 0.87, 95% CI 0.76 to 1.01; participants = 3169; studies = 15; low quality evidence) (Analysis 1.1). Similarly, for heart failure admission there was very little difference for either subgroup (usual care RR 0.72, 95% CI 0.53 to 0.99; participants = 319, studies = 2; clinical assessment RR 0.70, 95% CI 0.60 to 0.81; participants = 1609, studies = 10) to each other or the overall pooled result (RR 0.70, 95% CI 0.61 to 0.80; participants = 1928; studies = 10; low quality evidence) (Analysis 1.3).

Post‐hoc we explored the effect of duration of the intervention on outcomes. Analysis 6.1 shows that both at ≤ one year (RR 0.46, 95% CI 0.25 to 0.85; participants = 555; studies = 5; P =0.01; I^2^ = 0%) and between one and two years (RR 0.83, 95% CI 0.69 to 0.99; participants = 1842; studies = 8; P =0.04; I^2^ = 0%), there was a potential reduction for all‐cause mortality, but the evidence showed uncertainty at > two years (RR 1.11, 95% CI 0.87 to 1.41; participants = 772; studies = 2; P = 0.41; I^2^ = 0%) and the subgroup test for difference was significant (P =0.02). The effect of duration on heart failure admission shows a similar trend for each subgroup (≤ one year: RR 0.37, 95% CI 0.23 to 0.58; participants = 278; studies = 3, one to two years: RR 0.65, 95% CI 0.54 to 0.79; participants = 878; studies = 5; > two years: RR 0.97, 95% CI 0.77 to 1.23; participants = 772; studies = 2), again the test for subgroup effect was significant (P = 0.0004) Analysis 6.3. For heart failure mortality (Analysis 6.2), all‐cause admission (Analysis 6.4) and quality of life (Analysis 6.5), the subgroups all showed uncertainty similar to the overall pooled result for each outcome.

Post hoc we also explored the assumption that the two biomarkers were sufficiently biologically and clinical similar to evaluate together. We investigated this by separating the pooled data by each biomarker. For all‐cause mortality (Analysis 7.1), heart failure mortality (Analysis 7.2), all‐cause admission (Analysis 7.4) and quality of life (Analysis 7.5), the pooled data for each biomarker showed uncertainty and were similar to the overall pooled result for each outcome. For heart failure admission, using a fixed‐effect model, the result grouping the trials by BNP (Anguita 2010; Jourdain 2007; Karlstrom 2011; Krupicka 2010), or NT‐ProBNP (Berger 2010; Januzzi 2011; Lainchbury 2010; Schou 2013; Skvortsov 2015; Troughton 2000) did not make a difference to the main findings (BNP: RR 0.70, 95% CI 0.56 to 0.87; participants = 600; studies = 4; NT‐proBNP: RR 0.70, 95% CI 0.59 to 0.84; participants = 1328; studies 6) Analysis 7.3. In view of the substantial heterogeneity we tested the robustness of this finding using a random‐effects model and found that the pooled result for studies using the BNP marker continued to favour NP‐guided treatment but now showed uncertainty (BNP: RR 0.68, 95% CI 0.43 to 1.05; participants = 600; studies = 4; NT‐proBNP: RR 0.65, 95% CI 0.48 to 0.89; participants = 1328; studies 6).

#### Sensitivity analysis

Risk of bias within the studies varied across the aspects of bias assessed. Blinding of participants and study personnel appeared to be poor (see Figure 3 and Figure 4), nevertheless, it was not always practical to blind participants and personnel in some studies. High risk in this category could still mean one party was blinded. Blinding of outcome assessment and attrition was judged to potentially impact on the pooled results.

Sensitivity analyses were completed restricting studies to those with low risk of bias for blinding of outcome assessment (Berger 2010; Eurlings 2010; Karlstrom 2011; Lainchbury 2010; Schou 2013) and for attrition (Anguita 2010; Berger 2010; Jourdain 2007; Li 2015; Schou 2013; Shah 2011; Skvortsov 2015; Troughton 2000). For all outcomes, the analyses produced a similar effect to the main findings (see Table 5). Though there was only one study (Karlstrom 2011) assessed as low risk for detection bias for heart failure mortality and therefore no comparison with the main findings could be made in this instance.

**4:** Sensitivity Analyses

	Outcome	Studies(N)	Participants (n)	Risk ratio	95% Confidence intervals
Outcome blinding (low risk of bias studies only)					
Analysis 4.1	All‐cause mortality	5	1663	0.94	0.80 to 1.11
Analysis 4.2	Heart failure mortality	1	268	1.20	0.66 to 2.20
Analysis 4.3	Heart failure admission	4	1318	0.83	0.71 to 0.98
Analysis 4.4	All‐cause admission	2	675	0.98	0.88 to 1.10
Analysis 4.5	Quality of life	3	994	‐0.01	‐1.28 to 1.27
Incomplete data (low risk of bias studies only)					
Analysis 5.1	All‐cause mortality	7	1229	0.83	0.65 to 1.07
Analysis 5.2	Heart failure mortality	4	533	0.52	0.26 to 1.03
Analysis 5.3	Heart failure admission	5	814	0.63	0.49 to 0.81
Analysis 5.4	All‐cause admission	4	833	0.94	0.83 to 1.07
Analysis 5.5	Quality of life	3	534	‐0.57	‐1.92 to 0.78

## Discussion

### Summary of main results

We found the evidence for NP‐guided treatment in patients with heart failure showed uncertainty for all‐cause mortality or heart failure mortality. Furthermore, it showed uncertainty for all‐cause mortality when examining subgroups under or over 75 years of age. Heart failure admission was reduced, but evidence for all‐cause admission showed uncertainty. In addition, the evidence showed uncertainty for NP‐guided treatment improving quality of life. We were not able to pool results for adverse events and cost. All results were pooled from low‐quality evidence except the outcome quality of life where the quality level of evidence was very low (see Table 1). The up‐ or down‐titration of medication varied across studies in terms of the guidelines or algorithms used and changes in medication; neither was the reporting of NT levels consistent across studies. This meant we were unable to evaluate the impact of either of these for heart failure admission.

### Overall completeness and applicability of evidence

Our review included 18 studies, which recruited 3660 participants. The age of the participants in the studies may have favoured younger patients as the average age of participants ranged from 62 to 80 years old; however, New York Heart Association (NYHA) functional classification varied sufficiently across trials to ensure a broad range of severity. We were unable to assess a number of important subgroups; particularly, severity of heart failure at baseline, which may underpin an important effect of NP‐guided treatment on mortality outcomes. A systematic review in heart failure patients including 19 studies reported for each 100 pg/mL increase in BNP there was an associated 35% increase in the relative risk of death (Doust 2005). Further to this, subgroup analysis of baseline NP, and NP decrease, which could underpin the mechanism of effect, was not possible. In addition, a number of analyses were limited by lack of reporting: only six studies reported on all‐cause admission, there were limited data on costs and only six studies reported on adverse events.

### Quality of the evidence

All included studies were reported as randomised, but not all reported on the methods of randomisation. Eight confirmed allocation concealment and were judged to be at low risk of bias, and the other 10 were classified as unclear. Blinding was often poorly done with only one study reporting blinding of both participants and study personnel to treatment allocation, and only five studies reported blinding outcome assessors. Fourteen studies reported outcomes on an intention‐to‐treat basis and attrition bias, eight studies were judged to be low risk as seven studies had no losses to follow‐up, and the one fully documented the reported losses.

Using the Grading of Recommendations Assessment, Development and Evaluation (GRADE) approach, we assessed the quality of the evidence and GRADE profiler (GRADEPRO) was used to import data from Review Manager to create a 'Summary of findings' (SoF) table. For overall quality of evidence, the primary outcome plus heart failure mortality, heart failure admission and all‐cause admission were judged to have low quality and quality of life was judged to be very low quality indicating low/very low confidence in the pooled result, but that the result could vary and is likely to be affected by future research. The quality of evidence for adverse events and cost, which were not pooled, were also judged to be low. Quality of evidence was downgraded predominantly for limitations in the study design and/or inconsistency in the data.

### Potential biases in the review process

Whilst we did perform a thorough search with no date or language restrictions, it is possible some studies may have been overlooked in searching and study selection. We were unable to include data from one study for the primary outcome. Whilst only 15 studies contributed data for the funnel plot for all‐cause mortality, the graph does display a slight asymmetry with a lack of smaller studies showing a beneficial control effect. This suggests the potential for publication bias (see Figure 2).

### Agreements and disagreements with other studies or reviews

At least 12 reviews have been undertaken on the effects of NP‐guided treatment: three narrative reviews (De Vecchis 2013a; De Beradinis 2012; Richards 2012), one systematic review with no meta‐analysis ( Balion 2014), and eight reviews that included meta‐analyses (De Vecchis 2014; Felker 2009; Li 2013; Li 2014; Porapakkham 2010; Savarese 2013; Troughton 2014; Xin 2015). Of these meta‐analyses, seven reported one or more of the same outcome measures as this review*,* whilst De Vecchis 2014 only examined a composite outcome.

Five of the seven previous reviews reported NP reduced all‐cause mortality in heart failure patients and the other two, similar to this review, reported no effect for all‐cause mortality. No previous review has examined heart failure mortality as an outcome. All‐cause admission was analysed in three of the previous reviews and no effect was reported in agreement with our findings. Similar to this review, five previous reviews have reported an effect favouring NP‐guided treatment when examining heart failure admission and all reported a moderate level of heterogeneity. Two reviews examined adverse events and reported no reduction in events for NP‐guided patients compared to clinical assessment. To date, no review has examined costs, and only one previous review (Xin 2015) has reported on quality of life (see Table 6).

**5:** Agreements and disagreements with other reviews

Outcome	Review	Number of RCTs	N	Summary measure (hazard ratio HR, risk ratio RR, odds ratio OR, weighted mean difference WMD)		95% Confidence intervals	p‐value	Heterogeneity (I^2^)
All‐cause mortality (all patients)	Felker 2009	6	1627	HR	0.69	0.55 to 0.86	Not reported	Not reported
	Porapakkham 2010	8	1726	RR	0.76	0.63 to 0.91	0.003	Not reported
	Li 2013	11	2414	RR	0.83	0.69 to 0.99	0.0.35	0%
	Savarese 2013	12	2686	OR	0.74	0.6 to 0.91	0.005	0%
	Li 2014	Not reported	Not reported	RR	0.79	0.67 to 0.92	0.004	Not reported
	Troughton 2014	10	2280	HR	0.82	0.67 to 1.00	0.05	0%
	Xin 2015	14	3004	RR	0.94	0.81 to 1.08	0.39	3%
	This review	15	3169	RR	0.87	0.76 to 1.01	0.06	16%
Heart failure admission	Li 2013	7	1190	RR	0.65	0.5 to 0.84	0.001	52.30%
	Savarese 2013	8	1920	OR	0.55	0.4 to 0.77	<0.0001	58.20%
	Li 2014	Not reported	Not reported	RR	0.67	0.46 to 0.97	0.03	Not reported
	Troughton 2014	11	2431	HR	0.74	0.60 to 0.90	0.002	24.00%
	Xin 2015	11	2572	RR	0.79	0.63 to 0.98	0.03	67.00%
	This review	10	1928	RR	0.7	0.61 to 0.80	<0.0001	60.00%
All‐cause admission	Porapakkham 2010	3	330	RR	0.82	0.64 to 1.05	0.12	Not reported
	Savarese 2013	5	1108	OR	0.8	0.63‐ 1.02	0.077	0%
	Xin 2015	7	1627	RR	0.97	0.89 to 1.07	0.56	8%
	This review	6	1142	RR	0.93	0.84 to 1.03	0.15	0%
Adverse events	Li 2014	Not reported	Not reported	RR	1.15	0.99 to 1.342	0.69	Not reported
Adverse events (symptomatic hypotension)	Xin 2015	4	838	RR	1.72	0.59 to 5.05	0.32	43%
Adverse events (hyper/hypokalemia)	Xin 2015	2	354	RR	1.34	0.42 to 4.34	0.62	0%
Adverse events (renal dysfunction)	Xin 2015	3	769	RR	1.46	0.34 to 6.24	0.21	0%
Adverse events (severe cough)	Xin 2015	2	220	RR	1.93	0.69 to 5.37	0.21	0%
Quality of life	Xin 2015	5	1172	WMD	‐1.29	‐3.81 to 1.22	0.31	49%
	This review	8	1812	WMD	‐0.03	‐1.18 to 1.13	0.97	75%

The meta‐analysis published in 2014, Troughton 2014, included individual patient data (IPD) from nine trials and aggregate data sets from two trials and reported no effect in all‐cause mortality. Though, with the advantage of IPD Troughton and colleagues were able to adjust for patient characteristics and used Kaplan Meier curves to compare time to all‐cause mortality between NP‐guided and clinically‐guided treatment groups and they reported a reduction in all‐cause mortality (hazard ratio (HR) = 0.62; 95% CI, 0.45 to 0.86; P = 0.004, nine IPD studies). Similar to Porapakkham 2010, but again using time to event data, mortality was reduced in those under 75 years of age (HR 0.62; 95% CI, 0.45 to 0.85; P = 0.004), but not in those 75 years and older (HR 0.98; 95% CI, 0.75 to 1.3; P = 0.96), and the test of interaction between age and treatment effect was significant (P = 0.028). Hospitalisation due to heart failure was reduced in patients with NP‐guided therapy, both using time to event data (HR 0.80, 95% CI 0.67 to 0.94, P = 0.009), however, there was no effect for all‐cause hospitalisation using time to event data (HR 0.94, 95% CIs 0.84 to 1.07, P = 0.38).

While not directly comparable to this review, De Vecchis 2014 included six randomised controlled trials (RCTs) (n =  1775 patients) in a systemic review of BNP peptide‐guided versus symptom‐guided therapy in outpatients with chronic heart failure. This review reported guided therapy decreased a composite outcome of mortality and heart failure hospitalisations during the follow‐up period (odds ratio (OR) 0.64; 95%CI: 0.43 to 0.95; P  =  0.028, I^2^ = not reported).

Some subgroup analyses have been completed by previous reviews which can be compared to this review’s subgroup analyses (see Table 7). Only Porapakkham 2010 is directly comparable to this review and similarly reported for all‐cause mortality in patients over 75 years old an uncertain result. However, in patients under 75 years, unlike this review, Porapakkham 2010 reported a significant effect for NP monitoring compared to clinical assessment.

**6:** Subgroup agreements and disagreements with other reviews

Outcome	Review	Number of RCTs	N	Summary measure (hazard ratio HR, risk ratio RR, odds ratio OR, weighted mean difference WMD)		95% Confidence intervals	P value	Heterogeneity (I^2^)
All‐cause mortality (< 75 years)	Porapakkham 2010	2	741	RR	0.52	0.33 to 0.82	0.005	Not reported
	This review	3	420	RR	0.73	0.49 to 1.10	0.13	58%
All‐cause mortality (> 75 years)	Porapakkham 2010	2	741	RR	0.94	0.71 to 1.25	0.7	Not reported
	This review	3	410	RR	1.23	0.96 to 1.57	0.1	58%
All‐cause mortality (< 72 years)	Xin 2015	7	Not reported	RR	0.82	0.58 to 1.17	Not reported	0%
All‐cause mortality (≥ 72 years)	Xin 2015	7	Not reported	RR	0.96	0.83 to 1.13	Not reported	24%
Heart failure admission (<70 years)	Li 2013	Not reported	Not reported	RR	0.45	0.33 to 0.61	< 0.0001	0%
	Li 2014	Not reported	Not reported	RR	0.44	0.31 to 0.63	Not reported	Not reported
Heart failure admission (>70 years)	Li 2013	Not reported						
	Li 2014	Not reported	Not reported	RR	0.89	0.74 ‐ 1.07	Not reported	Not reported
All‐cause admission (< 72 years)	Xin 2015	5	Not reported	RR	0.61	0.41 to 0.93	Not reported	65%
All‐cause admission (≥ 72 years)	Xin 2015	6	Not reported	RR	0.95	0.79 to 1.14	Not reported	38%
All‐cause admission (< 72 years)	Xin 2015	4	Not reported	RR	0.88	0.77 to 1.00	Not reported	0%

Li 2013 reported heart failure admissions were reduced in patients with higher baseline BNP ≥2114 pg/mL (RR, 0.53; 95% CI, 0.39‐ to 0.72; P < 0.0001, I^2^ = 21.8%). Furthermore, Li 2014 completed sensitivity analyses to show a reduction in all‐cause mortality and heart failure admission was especially seen in patients with reduced ejection function.

This review is consistent with previous reviews in all outcomes except all‐cause mortality. For this outcome, the first (chronological) five reviews (Felker 2009; Porapakkham 2010; Li 2013; Savarese 2013; Li 2014) found a reduction, while Troughton 2014 found a reduction after adjustment for patient characteristics. The latest systematic review by Xin 2015 reported no effect on this outcome, similar to this review. One of the latest published trial (Schou 2013) reports higher all‐cause mortality in the NP‐guided group. The pooled estimate of effect based on exclusion of this study shows a reduction in all‐cause mortality similar to previous systematic reviews. Therefore, the inconsistency in this estimate leads us to suggest that further evaluation is required.

## Authors' conclusions

**Implications for practice**

This review confirms the evidence base to date, with at least four systematic reviews and one individual patient meta‐analysis published, of the efficacy of NP‐guided treatment effects on heart failure admission. Our post hoc analysis for this outcome demonstrates that effects are observed in shorter studies, less than two years in duration. This effect observed in the shorter studies could reflect the severity of the disease process whereby many patients would be hospitalised or experience adverse events with NP‐guided treatment having an impact delaying short‐term outcomes.

Although previous reviews consistently report a reduction for all‐cause mortality, our review, the largest to date reports low‐quality evidence that long‐term, all‐cause mortality and heart failure mortality show uncertainty. Furthermore, low‐quality evidence showed uncertainty for all‐cause admissions and very low quality of evidence showed uncertainty for quality of life outcomes.

**Implications for research**

There are a number of significant ongoing trials, therefore we do not perceive the need for any more until these have reported their results; but the significance around our results may change in the light of new data. We will update our review once these new trials are published, and we recommend updating the IPD analysis and using these data to perform cost‐effective analyses. Cost‐effectiveness data would aid decision making, particularly as length of hospital stay and preventing readmissions are important for the health service. In addition, it is important to clearly describe the components of the intervention and of the control group, as subtle changes in the control group in combination with a lack of blinding could have significant effects on treatment escalation and the overall efficacy of the intervention. In case a future update identifies an effect in mortality, the potential mechanisms for this effect, such as increased patient and physician adherence to treatment regimens, would need to be explored.

## Appendices

### Appendix 1. Search strategies

**Cochrane Central Register of Controlled Trials Database [the Cochrane Library, Wiley] (Issue 2 of 12, 2016), Database of Abstracts of reviews of Effectiveness & NHS Economic Evaluation Database [the Cochrane Library, Wiley] (Issue 2 of 4, 2015)**

**Table:**

#1	MeSH descriptor: [Heart Failure] this term only
#2	heart failure or chf or hf:ti,ab,kw (Word variations have been searched)
#3	#1 or #2
#4	MeSH descriptor: [Natriuretic Peptide, Brain] explode all trees
#5	b type natriuretic peptide*:ti,ab,kw (Word variations have been searched)
#6	brain natriuretic peptide*:ti,ab,kw (Word variations have been searched)
#7	brain type natriuretic peptide*:ti,ab,kw (Word variations have been searched)
#8	pro bnp:ti,ab,kw (Word variations have been searched)
#9	probnp:ti,ab,kw (Word variations have been searched)
#10	ntpprobnp:ti,ab,kw (Word variations have been searched)
#11	natriuretic peptide type b:ti,ab,kw (Word variations have been searched)
#12	#4 or #5 or #6 or #7 or #8 or #9 or #10 or #11
# 13	MeSH descriptor: [Monitoring, Physiologic] this term only
#14	MeSH descriptor: [Prognosis] this term only
#15	MeSH descriptor: [Treatment Outcome] this term only
#16	monitor*:ti,ab,kw (Word variations have been searched)
#17	((serial or routine or longterm or long term) near/2 (measure* or test* or follow up)):ti,ab,kw (Word variations have been searched)
#18	((guide* or target*) near/2 (therap* or treatment* or pharmacotherap* or strateg*)):ti,ab,kw (Word variations have been searched)
#19	prognos*:ti,ab,kw (Word variations have been searched)
#20	retest*:ti,ab,kw (Word variations have been searched)
#21	#13 or #14 or #15 or #16 or #17 or #18 or #19 or #20
#22	#3 and #12 and #21

**Embase (OvidSP)(1974‐14/3/16)**

**Table:**

1	Heart Failure/
2	Congestive Heart Failure/
3	(heart failure or hf or chf).tw.
4	1 or 2 or 3
5	brain natriuretic peptide/
6	b type natriuretic peptide*.tw.
7	brain natriuretic peptide*.tw.
8	brain type natriuretic peptide*.tw.
9	bnp*.tw.
10	probnp*.tw.
11	pro bnp*.tw.
12	nt probnp.tw.
13	ntprobnp.tw.
14	natriuretic peptide type b.tw.
15	5 or 6 or 7 or 8 or 9 or 10 or 11 or 12 or 13 or 14
16	Patient monitoring/
17	Biologic monitoring/
18	Prognosis/
19	treatment outcome/
20	Follow up/
21	monitor*.tw.
22	((serial or routine or longterm or long term) adj2 (measure* or test* or follow up)).tw.
23	((guide* or target*) adj2 (therap* or treatment* or pharmacotherap* or strateg*)).tw.
24	prognos*.tw.
25	retest*.tw.
26	16 or 17 or 18 or 19 or 20 or 21 or 22 or 23 or 24 or 25
27	4 and 15 and 26
28	randomized controlled trial/
29	controlled clinical trial/
30	single blind procedure/ or double blind procedure/
31	crossover procedure/
32	random*.tw.
33	placebo*.tw.
34	((singl* or doubl*) adj (blind* or mask*)).tw.
35	(crossover or cross over or factorial* or latin square).tw.
36	(assign* or allocat* or volunteer*).tw.
37	28 or 29 or 30 or 31 or 32 or 33 or 34 or 35 or 36
38	27 and 37
39	(exp animal/ or nonhuman/) not human/
40	38 not 39

**MEDLINE (OvidSP)(1946‐15/3/16)**

**Table:**

1	Heart Failure/
2	(heart failure or hf or chf).tw.
3	1 or 2
4	Natriuretic Peptide, Brain/
5	b type natriuretic peptide*.tw.
6	brain natriuretic peptide*.tw.
7	brain type natriuretic peptide*.tw.
8	bnp*.tw.
9	probnp*.tw.
10	pro bnp*.tw.
11	nt probnp.tw.
12	ntprobnp.tw.
13	natriuretic peptide type b.tw.
14	4 or 5 or 6 or 7 or 8 or 9 or 10 or 11 or 12 or 13
15	Monitoring, Physiologic/
16	Prognosis/
17	treatment outcome/
18	monitor*.tw.
19	((serial or routine or longterm or long term) adj2 (measure* or test* or follow up)).tw.
20	((guide* or target*) adj2 (therap* or treatment* or pharmacotherap* or strateg*)).tw.
21	prognos*.tw.
22	retest*.tw.
23	15 or 16 or 17 or 18 or 19 or 20 or 21 or 22
24	3 and 14 and 23
25	randomized controlled trial.pt.
26	controlled clinical trial.pt.
27	randomized.ab.
28	placebo.ab.
29	drug therapy.fs.
30	randomly.ab.
31	trial.ab.
32	groups.ab.
33	25 or 26 or 27 or 28 or 29 or 30 or 31 or 32
34	exp animals/ not humans.sh.
35	33 not 34
36	24 and 35

**Science Citation Index & Conference Proceedings Citation Index – Science. (ISI Web of Science)(1945 ‐ 15/3/16)**

**Table:**

# 1	752,670	TS=("b‐type natriuretic peptide*") OR TS=(btype natriuretic peptide*) OR TS=("b type natriuretic peptide*") OR TS=("type‐b natriuretic peptide*") OR TS=("natriuretic peptide* type‐b") OR TS=("brain natriuretic peptide*") OR TS=("brain type natriuretic peptide*") OR TS=(bnp*) OR TS=(probnp* or "pro bnp*") OR TS=("nt probnp" or ntprobnp) OR TS=("natriuretic peptide type b")
# 2	17,530	TS=(monitor*) OR TS=(((serial OR routine OR longterm OR long term) SAME (measure* or test* or follow up))) OR TS=(((serial OR routine OR longterm OR long term) SAME (measure* or test* or follow up))) OR TS=(prognos*) OR TS=(retest*)
# 3	1,559,464	2 AND 1
# 4	5,037	TS=(((random* or blind* or allocat* or assign* or trial* or placebo* or crossover* or cross‐over*)))
# 5	2,233,989	4 AND 3

**ClinicalTrials.gov (15/3/16)**

**Table:**

Title=natriuretic peptide OR bnp OR pro bnp OR probnp OR ntprobnp OR pro‐bnp OR nt‐probnp
Intervention=natriuretic peptide OR bnp OR pro bnp OR probnp OR ntprobnp OR pro‐bnp OR nt‐probnp

**WHO ICTRP (15/3/16)**

**Table:**

Title=natriuretic peptide OR bnp OR pro bnp OR probnp OR ntprobnp OR pro‐bnp OR nt‐probnp
Intervention=natriuretic peptide OR bnp OR pro bnp OR probnp OR ntprobnp OR pro‐bnp OR nt‐probnp

## Data and analyses

**Comparison 1:** Primary objective BNP vs no BNP

Outcome or subgroup title	No. of studies	No. of participants	Statistical method	Effect size
1 All‐cause mortality	15	3169	Risk Ratio (M‐H, Fixed, 95% CI)	0.87 [0.76, 1.01]
2 Heart failure mortality	6	853	Risk Ratio (M‐H, Fixed, 95% CI)	0.84 [0.54, 1.30]
3 Heart failure admission	10	1928	Risk Ratio (M‐H, Fixed, 95% CI)	0.70 [0.61, 0.80]
4 All‐cause admission	6	1142	Risk Ratio (M‐H, Fixed, 95% CI)	0.93 [0.84, 1.03]
5 Quality of life	8	1812	Mean Difference (IV, Fixed, 95% CI)	‐0.03 [‐1.18, 1.13]

**Comparison 2:** Clincal vs UC in primary objectives

Outcome or subgroup title	No. of studies	No. of participants	Statistical method	Effect size
1 All‐cause mortality	15	3169	Risk Ratio (M‐H, Fixed, 95% CI)	0.87 [0.76, 1.01]
1.1 Clinical assessment	15	2850	Risk Ratio (M‐H, Fixed, 95% CI)	0.89 [0.76, 1.04]
1.2 Usual care	2	319	Risk Ratio (M‐H, Fixed, 95% CI)	0.79 [0.56, 1.13]
2 Heart failure mortality	6	853	Risk Ratio (M‐H, Fixed, 95% CI)	0.84 [0.54, 1.30]
2.1 Clinical assessment	6	853	Risk Ratio (M‐H, Fixed, 95% CI)	0.84 [0.54, 1.30]
2.2 Usual care	0	0	Risk Ratio (M‐H, Fixed, 95% CI)	0.0 [0.0, 0.0]
3 Heart failure admission	10	1928	Risk Ratio (M‐H, Fixed, 95% CI)	0.70 [0.61, 0.80]
3.1 Clinical assessment	10	1609	Risk Ratio (M‐H, Fixed, 95% CI)	0.70 [0.60, 0.81]
3.2 Usual care	2	319	Risk Ratio (M‐H, Fixed, 95% CI)	0.72 [0.53, 0.99]
4 All‐cause admission	6	1142	Risk Ratio (M‐H, Fixed, 95% CI)	0.93 [0.84, 1.03]
4.1 Clinical assessment	6	1142	Risk Ratio (M‐H, Fixed, 95% CI)	0.93 [0.84, 1.03]
4.2 Usual care	0	0	Risk Ratio (M‐H, Fixed, 95% CI)	0.0 [0.0, 0.0]
5 Quality of life	8	1812	Mean Difference (IV, Fixed, 95% CI)	‐0.03 [‐1.18, 1.13]
5.1 Clincial assessment	8	1812	Mean Difference (IV, Fixed, 95% CI)	‐0.03 [‐1.18, 1.13]
5.2 Usual care	0	0	Mean Difference (IV, Fixed, 95% CI)	0.0 [0.0, 0.0]

**Comparison 3:** Subgroup analyses

Outcome or subgroup title	No. of studies	No. of participants	Statistical method	Effect size
1 All‐cause mortality and age	3	830	Risk Ratio (M‐H, Fixed, 95% CI)	1.02 [0.83, 1.27]
1.1 Equal or greater than 75 yrs old	3	410	Risk Ratio (M‐H, Fixed, 95% CI)	1.23 [0.96, 1.57]
1.2 Under 75 yrs old	3	420	Risk Ratio (M‐H, Fixed, 95% CI)	0.73 [0.49, 1.10]
2 Heart failure admission and age	1	365	Risk Ratio (M‐H, Fixed, 95% CI)	0.93 [0.69, 1.25]
2.1 Equal or greater than 75 yrs old	1	188	Risk Ratio (M‐H, Fixed, 95% CI)	1.13 [0.77, 1.64]
2.2 Under 75 yrs old	1	177	Risk Ratio (M‐H, Fixed, 95% CI)	0.73 [0.45, 1.17]

**Comparison 4:** Sensitivity analyses: Outcome blinding

Outcome or subgroup title	No. of studies	No. of participants	Statistical method	Effect size
1 All‐cause mortality	5	1663	Risk Ratio (M‐H, Fixed, 95% CI)	0.94 [0.80, 1.11]
2 Heart failure mortality	1	268	Risk Ratio (M‐H, Fixed, 95% CI)	1.2 [0.66, 2.20]
3 Heart failure admission	4	1318	Risk Ratio (M‐H, Fixed, 95% CI)	0.83 [0.71, 0.98]
4 All‐cause admission	2	675	Risk Ratio (M‐H, Fixed, 95% CI)	0.98 [0.88, 1.10]
5 Quality of life	3	994	Mean Difference (IV, Fixed, 95% CI)	‐0.01 [‐1.28, 1.27]

**Comparison 5:** Sensitivity analyses: Attrition

Outcome or subgroup title	No. of studies	No. of participants	Statistical method	Effect size
1 All‐cause mortality	7	1229	Risk Ratio (M‐H, Fixed, 95% CI)	0.83 [0.65, 1.07]
2 Heart failure mortality	4	533	Risk Ratio (M‐H, Fixed, 95% CI)	0.52 [0.26, 1.03]
3 Heart failure admission	5	814	Risk Ratio (M‐H, Fixed, 95% CI)	0.63 [0.49, 0.81]
4 All‐cause admission	4	833	Risk Ratio (M‐H, Fixed, 95% CI)	0.94 [0.83, 1.07]
5 Quality of life	3	534	Mean Difference (IV, Fixed, 95% CI)	‐0.57 [‐1.92, 0.78]

**Comparison 6:** Duration of FU BNP vs no BNP

Outcome or subgroup title	No. of studies	No. of participants	Statistical method	Effect size
1 All‐cause mortality	15	3169	Risk Ratio (M‐H, Fixed, 95% CI)	0.87 [0.76, 1.01]
1.1 ≤ 1 yr	5	555	Risk Ratio (M‐H, Fixed, 95% CI)	0.46 [0.25, 0.85]
1.2 1‐2 yrs	8	1842	Risk Ratio (M‐H, Fixed, 95% CI)	0.83 [0.69, 0.99]
1.3 > 2 yrs	2	772	Risk Ratio (M‐H, Fixed, 95% CI)	1.11 [0.87, 1.41]
2 Heart failure mortality	6	853	Risk Ratio (M‐H, Fixed, 95% CI)	0.84 [0.54, 1.30]
2.1 ≤ 1 yr	3	313	Risk Ratio (M‐H, Fixed, 95% CI)	0.64 [0.28, 1.48]
2.2 1 ‐ 2 yrs	3	540	Risk Ratio (M‐H, Fixed, 95% CI)	0.94 [0.56, 1.57]
2.3 > 2 yrs	0	0	Risk Ratio (M‐H, Fixed, 95% CI)	0.0 [0.0, 0.0]
3 Heart failure admission	10	1928	Risk Ratio (M‐H, Fixed, 95% CI)	0.70 [0.61, 0.80]
3.1 ≤ 1 yr	3	278	Risk Ratio (M‐H, Fixed, 95% CI)	0.37 [0.23, 0.58]
3.2 1 ‐ 2 yrs	5	878	Risk Ratio (M‐H, Fixed, 95% CI)	0.65 [0.54, 0.79]
3.3 > 2 ys	2	772	Risk Ratio (M‐H, Fixed, 95% CI)	0.97 [0.77, 1.23]
4 All‐cause admission	6	1142	Risk Ratio (M‐H, Fixed, 95% CI)	0.93 [0.84, 1.03]
4.1 ≤ 1 yr	3	247	Risk Ratio (M‐H, Fixed, 95% CI)	0.79 [0.58, 1.07]
4.2 1 ‐ 2 yrs	2	488	Risk Ratio (M‐H, Fixed, 95% CI)	0.89 [0.77, 1.03]
4.3 > 2 yrs	1	407	Risk Ratio (M‐H, Fixed, 95% CI)	1.04 [0.89, 1.21]
5 Quality of life	8	1812	Mean Difference (IV, Fixed, 95% CI)	‐0.03 [‐1.18, 1.13]
5.1 ≤ 1 yr	5	561	Mean Difference (IV, Fixed, 95% CI)	‐3.14 [‐6.46, 0.19]
5.2 1 ‐ 2 yrs	2	844	Mean Difference (IV, Fixed, 95% CI)	1.98 [‐0.76, 4.72]
5.3 > 2 yrs	1	407	Mean Difference (IV, Fixed, 95% CI)	0.0 [‐1.38, 1.38]

**Comparison 7:** Subgroup: BNP vs NT‐proBNP

Outcome or subgroup title	No. of studies	No. of participants	Statistical method	Effect size
1 All‐cause mortality	15	3169	Risk Ratio (M‐H, Fixed, 95% CI)	0.87 [0.76, 1.01]
1.1 NT‐proBNP	9	2391	Risk Ratio (M‐H, Fixed, 95% CI)	0.87 [0.75, 1.01]
1.2 BNP	6	778	Risk Ratio (M‐H, Fixed, 95% CI)	0.89 [0.62, 1.28]
2 Heart failure mortality	6	853	Risk Ratio (M‐H, Fixed, 95% CI)	0.84 [0.54, 1.30]
2.1 NT‐proBNP	2	127	Risk Ratio (M‐H, Fixed, 95% CI)	0.30 [0.08, 1.19]
2.2 BNP	4	726	Risk Ratio (M‐H, Fixed, 95% CI)	0.98 [0.61, 1.56]
3 Heart failure admission	10	1928	Risk Ratio (M‐H, Random, 95% CI)	0.67 [0.53, 0.84]
3.1 NT‐proBNP	6	1328	Risk Ratio (M‐H, Random, 95% CI)	0.65 [0.48, 0.89]
3.2 BNP	4	600	Risk Ratio (M‐H, Random, 95% CI)	0.68 [0.43, 1.05]
4 All‐cause admission	6	1142	Risk Ratio (M‐H, Fixed, 95% CI)	0.93 [0.84, 1.03]
4.1 NT‐proBNP	2	476	Risk Ratio (M‐H, Fixed, 95% CI)	0.99 [0.85, 1.14]
4.2 BNP	4	666	Risk Ratio (M‐H, Fixed, 95% CI)	0.88 [0.77, 1.01]
5 Quality of life	8	1812	Mean Difference (IV, Fixed, 95% CI)	‐0.03 [‐1.18, 1.13]
5.1 NT‐proBNP	7	1771	Mean Difference (IV, Fixed, 95% CI)	‐0.02 [‐1.19, 1.14]
5.2 BNP	1	41	Mean Difference (IV, Fixed, 95% CI)	‐0.20 [‐15.30, 14.90]

## Characteristics of studies

### Characteristics of included studies [ordered by study ID]

**Table:** Anguita 2010

Methods	Setting: Hospital in Spain Duration of study: 18 months Inclusion criteria: At least NYHA III, receiving at least one diuretic, an ACE inhibitor or ARB and a beta blocker Exclusion criteria: < 18 years old, acute coronary syndrome within 3 months, aetiological treatment or cardiac transplantation pending, life expectancy < 1 year due to co‐morbidities	
Participants	Number of participants at baseline: Intervention 30; Control 30 Gender (male): Intervention 67%; Control 70% Mean age (SD): Intervention 70 (8); Control 69 (12)	
Interventions	BNP‐guided treatment: Minimum four visits in first quarter, six visits in first year, seven visits overall; structured clinical assessment including BNP data; if BNP levels were higher than 100 pg/mL, the pharmacological treatment was increased. Specifically: i) increased dose of loop diuretic; ii) doubling the dose of ACEi (max. 150 mg/d of captopril, 40 mg/d of enalapril, 10 mg/d of ramipril); iii) addition of spironolactone 25 mg/d to 50 mg/d (if not previously administered); iv) double dose of beta blocker (max. 50 mg/d of carvedilol or 10 mg/d of bisoprolol); v) addition of an ARB, at recommended doses; vi) addition of chlorthalidone 50 mg/d; vii) addition of digoxin 0.25 mg/d or adjusted to renal function; viii) other drugs: nitrates, amlodipine. If the target BNP is achieved the patient will follow the same treatment regimen as prior to the visit until the next scheduled visit. Control: Visits same as intervention without BNP data and additional visit at two weeks; treatment guided by less or greater Framingham score of two, recent events, questions to patient and medical history. If target score achieved the patient follow the same treatment regimen as prior to the visit until the next scheduled visit. Intervention provider: Specialist (cardiology service)	
Outcomes	Review relevant: i) All‐cause mortality; ii) HF admission Additional outcomes: i) Cardiovascular events	
Notes		
***Risk of bias***		
**Bias**	**Authors' judgement**	**Support for judgement**
Random sequence generation (selection bias)	Unclear risk	Randomised, but no description of how achieved
Allocation concealment (selection bias)	Unclear risk	Not stated
Blinding of participants and personnel (performance bias) All outcomes	Unclear risk	Not stated
Blinding of outcome assessment (detection bias) All outcomes	Unclear risk	Not stated
Incomplete outcome data (attrition bias) All outcomes	Low risk	No attrition
Selective reporting (reporting bias)	Low risk	All outcomes reported as specified in the publication
Other bias	Unclear risk	Source of funding: Not stated

**Table:** Beck‐da‐Silva 2005

Methods	Setting: Outpatient clinic in Canada Duration of study: Three months Inclusion criteria: Patients with symptomatic HF (NYHA II to IV) for 3 months previous or previous hospital admission due to HF, not on beta blockers, LVEF 40% or less, receiving treatment with an ACE inhibitor or ARB plus loop diuretic and digoxin Exclusion criteria: < 18 years old, one of the following: myocardial infarction or unstable angina within 4 weeks, severe stenotic valvular heart disease or hepatic or renal disease or a contraindication for beta blockers	
Participants	Number of participants at baseline: Intervention 21; Control 20 Gender (male): Intervention 33.3%; Control 35% Mean age (SD): Intervention 64.5 (15.2); Control 65.6 (13.5)	
Interventions	BNP‐guided treatment: Minimum four visits in first quarter, four visits overall; structured clinical assessment including BNP data, beta blocker up‐titration based on starting at 1.25‐2.5 mg/d and titrated up to 10 mg/d. Action taken based on four scenarios: i) clinically better, BNP decreasing: β blocker increased one step; ii) clinically same or mildly worse, BNP decreasing: β blocker increased one step; iii) clinically same or better, BNP increasing: β blocker unchanged; iv) clinically worse, BNP increasing: β blocker decreased one step or discontinued Clincial assessment (control): Visits same as intervention without BNP data, treatment dose increase according to clinical status assessed by attending physician. Up‐titration of β blocker if worsening function Intervention provider: Specialist (HF team)	
Outcomes	Review relevant: i) All‐cause mortality; ii) All‐cause admission iii); Quality of Life Additional outcomes: i) LVEF change	
Notes		
***Risk of bias***		
**Bias**	**Authors' judgement**	**Support for judgement**
Random sequence generation (selection bias)	Unclear risk	'Randomly assigned'. No description of how achieved
Allocation concealment (selection bias)	Low risk	Email from author 19 September 14 "'opaque envelopes"
Blinding of participants and personnel (performance bias) All outcomes	High risk	"BNP values were blinded to the attending physician in the clinical group... (control)... but the doctors were not blinded as to which group the patient belonged"
Blinding of outcome assessment (detection bias) All outcomes	Unclear risk	Not stated
Incomplete outcome data (attrition bias) All outcomes	Unclear risk	Email from author 19 September 14 "There was very few missing data. I believe the participants were then excluded"
Selective reporting (reporting bias)	Low risk	All outcomes reported as specified in the publication
Other bias	Unclear risk	Source of funding: Not stated

**Table:** Berger 2010

Methods	Setting: Hospital and community in Austria Duration of study: 18 months Inclusion criteria: Clincial signs and symptoms of cardiac decompensation at hospitalisation, NYHA III or IV at admission, cardiothoracic ratio > 0.5 or LVEF < 40% Exclusion criteria: None stated	
Participants	Number of participants at baseline: Intervention (BM) 92; Control (MC) 96; Control (UC) 90 Gender (male): Intervention (BM) 63%; Control (MC) 70%; Control (UC) 69% Mean age (SD): Intervention (BM) 70 (12); Control (MC) 73 (11); Control (UC) 71 (13)	
Interventions	NT‐proBNP‐guided intensive management (BM): > 2200 pg/mL at hospital discharge; minimum six visits in first quarter, eight in first year and 8 to 26 visits overall; structured clinical assessment including NT‐proBNP data at outpatient clinic; as long as NT‐proBNP remained above 2200 pg/mL drug treatments were dictated by a flow chart until maximum or tolerated doses of HF drugs were established. If NT‐proBNP fell below 2200 pg/mL 3 or 6 months after discharge then patients reverted to following the treatment schedule for the control group (MC) Multidisplinary care (MC, control): < 2200 pg/mL at hospital discharge; minimum four visits in first quarter, six in first year and six visits overall; structured clinical assessment without NT‐proBNP data via home visits; treatment dose increase according to clinical status assessed by HF nurse Usual care (UC, control): No visit schedule or structured follow‐up. HF specialist only on request Intervention provider: HF specialist (BM), HF nurse (MC), Primary care physician (UC)	
Outcomes	Review relevant: i) All‐cause mortality; ii) HF mortality; iii) HF admission; iv) All‐cause admission; v) Quality of life Additional outcomes: i) Time to death or HF admission; ii) Ambulatory visits at HF clinics	
Notes		
***Risk of bias***		
**Bias**	**Authors' judgement**	**Support for judgement**
Random sequence generation (selection bias)	Low risk	Computer‐generated permuted block randomisation. 6 patients per block
Allocation concealment (selection bias)	Low risk	Randomisation and concealment completed by independent medical project management institute
Blinding of participants and personnel (performance bias) All outcomes	High risk	"Patients and providers knew they were in an intervention group (BM and MC)"
Blinding of outcome assessment (detection bias) All outcomes	Low risk	"Independent data collectors obtained information from medical reports and interviews with relatives". Cardologists blinded to treatment classified the cause of hospitalisation
Incomplete outcome data (attrition bias) All outcomes	Low risk	No attrition
Selective reporting (reporting bias)	High risk	Planned outcomes specified in Berger 2010. Data not reported for HF mortality, all‐cause admission
Other bias	Unclear risk	Source of funding: AstraZeneca, Novartis, Roche Diagnostics, Roche Medical, Merck, Medtronic, and Guidant, who provided the financial support for a clinical investigator, a specialised chronic HF nurse, and data collection

**Table:** Eurlings 2010

Methods	'PRIMA' Setting: 12 hospitals in the Netherlands Duration of study: 24 months Inclusion criteria: European Society of Cardiology (ESC) diagnostic guideline criteria for acute HF, NT‐proBNP levels at admission were required to be at least 1,700 pg/mL, NT‐proBNP levels during hospitalisation were required to decrease more than 10%, with a drop in NT‐proBNP levels of at least 850 pg/mL, from admission to discharge Exclusion criteria: Life‐threatening cardiac arrhythmias during the index hospitalisation, urgent invasive or surgical intervention performed or planned during the index hospital admission, severe COPD with a forced expiratory volume in 1 s (FEV1) of 1 l/s, pulmonary embolism less than 3 months prior to admission, pulmonary hypertension not caused by left ventricular systolic dysfunction (LVSD), a non–HF‐related expected survival of less than 1 year, and patients undergoing haemodialysis or CAPD	
Participants	Number of participants at baseline: Intervention 174; Control 171 Gender (male): Intervention 55%; Control 60% Mean age (SD): Intervention 71.6 (12); Control 72.8 (11.7)	
Interventions	NT‐proBNP‐guided treatment: minimum three visits in first quarter, six in first year and estimated 10 visits overall; structured clinical assessment including NT‐proBNP data; individual patient NT‐proBNP target value was set as the lowest level at discharge or at 2 weeks follow‐up. If NT‐proBNP levels were more than 10% with a minimum of 850 pg/mL above this individual target level, NT‐proBNP level was considered “off‐target,” and therapy was intensified according to the ESC HF treatment guidelines. They report changes in 10 different medications. Except for calcium channel blockers, all changes in drug therapies concern the start or increase of medication or change in the type of medication. It was not specifically stated if no/any action was taken if the patient was below or at target. Clincially‐guided (control): Visits same as intervention without NT‐proBNP data, treatment dictated by clinical assessment alone. Intervention provider: Specialist (HF cardiologists and nurses)	
Outcomes	Review relevant: i) All‐cause mortality; ii) Quality of life Additional outcomes: i) Survival free of hospitalisation; ii) Cardiovascular mortality; iii) Cardiovascular admissions; vi) Composite of total cardiovascular morbidity and mortality	
Notes		
***Risk of bias***		
**Bias**	**Authors' judgement**	**Support for judgement**
Random sequence generation (selection bias)	Unclear risk	'Randomised to'. No description of how achieved
Allocation concealment (selection bias)	Low risk	Email from author 23 October 14 "completed by non‐transparent envelopes"
Blinding of participants and personnel (performance bias) All outcomes	High risk	Email from author 23 October 14 "Patients were blinded to the treatment allocation. The treating physician however was not."
Blinding of outcome assessment (detection bias) All outcomes	Low risk	"All events were adjudicated by a blinded event committee, consisting of medical specialists in cardiology, nephrology, vascular medicine, pulmonology, and neurology."
Incomplete outcome data (attrition bias) All outcomes	Unclear risk	One‐year attrition documented with reasons. Unclear beyond 1 year
Selective reporting (reporting bias)	High risk	Planned outcomes specified in Eurlings 2010. No data reported for all‐cause admission
Other bias	Unclear risk	Source of funding: Main funding from the Netherlands heart foundation, Netherlands organisation for scientific research and Royal Netherlands academy of arts and sciences‐inter university cardiology institute of the Netherlands. Minor funding of an unrestricted fund was provided by Pfizer

**Table:** Januzzi 2011

Methods	'PROTECT' Setting: Hospital in USA Duration of study: 12 months Inclusion criteria: ≥ 21 years old, LVEF ≤ 40%, NYHA class II ‐ IV, hospital admission, emergency dept. or outpatient therapy for destabilised HF at least once in last 6 months Exclusion criteria: Serum creatinine >2.5 mg/dL, inoperable aortic valvular heart disease, life expectancy < 1 year due to causes other than HF, cardiac implant or revascularisation indicated or expected within 6 months, severe obstructive or restrictive pulmonary disease, unwilling or unable to give consent, coronary revascularisation within previous 3 months	
Participants	Number of participants at baseline: Intervention 75; Control 76 Gender (male): Intervention 88.2%; Control 81.3% Mean age (SD): Intervention 63 (14.5); Control 63.5 (13.5)	
Interventions	NT‐proBNP‐guided treatment: minimum two visits in first quarter, quarterly visits up to a maximum of 12 months (median number of visits for both arms was five); however scheduled visits were every two weeks until optimal/maximal medical therapy was achieved; structured clinical assessment including NT‐proBNP data at outpatient clinic; if NT‐proBNP levels were higher than 1000 pg/mL the drug therapy was intensified irrespective of clinical status; choice of medication therapy for either intervention arm was made by the physician according to consensus guidelines (American College of Cardiology foundation/American Association task force on practical guidelines); no algorithm for drug titration as used; once the patient achieved ≤ 1000 pg/mL (NT‐proBNP‐targeted optimal medical regimen) or if the target was not achieved but reached clear therapeutic limit then the patient will cease two weekly visits and revert to quarterly schedule. Standard of care treatment (control): Visits same as intervention without NT‐proBNP data, treatment dictated by clinical assessment and managed according to consensus guidelines. Once the patient achieves optimal medical regimen they will cease two‐weekly visits and revert to quarterly schedule. Intervention provider: Specialist (physicians skilled in HF care)	
Outcomes	Review relevant: i) HF admission; ii) Adverse events; iii) Cost; iv) Quality of life Additional outcomes: i) Total cardiovascular events in one year; ii) Cardiac structure and function; iii) Cost of care	
Notes		
***Risk of bias***		
**Bias**	**Authors' judgement**	**Support for judgement**
Random sequence generation (selection bias)	Low risk	Block randomisation
Allocation concealment (selection bias)	Unclear risk	Not stated
Blinding of participants and personnel (performance bias) All outcomes	High risk	'Neither caregivers nor the patients were blinded to the NT‐proBNP results'
Blinding of outcome assessment (detection bias) All outcomes	Unclear risk	Not stated
Incomplete outcome data (attrition bias) All outcomes	Unclear risk	Not stated
Selective reporting (reporting bias)	Low risk	All outcomes reported as specified in the protocol
Other bias	Unclear risk	Source of funding: In part by Roche diagnostics, Inc. First author partly funded by Roche Diagnostics, Inc., Siemens Diagnostics, and Critical Diagnostics

**Table:** Jourdain 2007

Methods	'STARS‐BNP' Setting: 17 hospitals in France Duration of study: Minimum six months Inclusion criteria: > 18 years old, NYHA II to III, LVEF < 45%, stable condition (no hospital stay in previous month) and treated by optimal therapy (ESC guidelines), dosages of medication stable for at least 1 month, diuretics, ACEs, ARBs, and β blockers at maximum tolerated doses Exclusion criteria: Acute coronary syndrome in last 3 months, chronic renal failure (plasma creatinine > 250 µmol/L), documented hepatic cirrhosis, asthma, or COPD	
Participants	Number of participants at baseline: Intervention 110; Control 110 Gender (male): Intervention 59%; Control 56% Mean age (SD): Intervention 65 (5); Control 66 (6)	
Interventions	BNP‐guided treatment: minimum four visits in first quarter, six in first year and overall; structured clinical assessment including BNP data at outpatient clinic; treatment modified according to judgment of investigator based on ESC guidelines 2001. It was not specifically stated if no/any action was taken if the patient was below or at target. Clinically‐guided treatment (control): Visits same as intervention without BNP data, medical therapy adjusted according to opinion of the investigator on basis of physical examination and biological parameters; treatment modified according to judgment of investigator based on ESC guidelines 2001 Intervention provider: Specialist (highly qualified cardiologists)	
Outcomes	Review relevant: i) All‐cause mortality; ii) HF mortality; iii) HF admission; iv) All‐cause admission Additional outcomes: i) Composite of HF mortality or HF hospital admissions	
Notes		
***Risk of bias***		
**Bias**	**Authors' judgement**	**Support for judgement**
Random sequence generation (selection bias)	Unclear risk	Randomised, but no description of how achieved
Allocation concealment (selection bias)	Unclear risk	Not stated
Blinding of participants and personnel (performance bias) All outcomes	High risk	Patients blinded to BNP results. BNP results only available to investigator to guide treatment
Blinding of outcome assessment (detection bias) All outcomes	Unclear risk	Not stated
Incomplete outcome data (attrition bias) All outcomes	Low risk	No attrition
Selective reporting (reporting bias)	Low risk	All outcomes reported as specified in the publication
Other bias	Unclear risk	Source of funding: Unrestricted grant from Biosite Inc. (San Diego, Calafornia) to the french working group on HF

**Table:** Karlstrom 2011

Methods	'UPSTEP' Setting: 19 hospitals in Sweden and Norway Duration of study: Minimum 12 months Inclusion criteria: > 18 years old, with verified systolic HF, worsening HF in last month (requiring hospitalisation, and/or intravenous diuretic treatment, metolazone, or increased daily doses of diuretics and /or need of intravenous inotropic support), LVEF < 40% (measured in last 6 months)4. NYHA II‐IV, ongoing standard HF treatment according to guidelines (ACE, ACEI, ARB, BB and/or diuretics, AA and/or digoxin if needed) Exclusion criteria: If any of the following conditions existed: haemodynamically unstable patients on waiting list for cardiac surgery, myocardial infarction within the last 3 months, patients with haemodynamically significant valvular heart disease, patients with impaired renal function (s‐creatinine >250 µmol/L) or liver function (> 3x normal value), patients with severely decreased pulmonary function, patients with limited life expectancy	
Participants	Number of participants at baseline: Intervention 147; Control 132 Gender (male): Intervention 73%; Control 73% Mean age (SD): Intervention 71.6 (9.7); Control 70.1 (10)	
Interventions	BNP‐guided treatment: minimum three visits in first quarter, seven in first year and overall ; structured clinical assessment including BNP data at outpatient clinic; treatment modified according to judgment of investigator based on ESC guidelines 2001. Specifically i) increase ACEi/ARB to maximum tolerated or target dose according to guidelines; ii) increase BB to maximum tolerated or target dose according to guidelines; iii) add AA in low dose (spironolactone 25 mg;) iv) add ARB and increase to target dose according to guidelines; v) increase ACEi/ARB to up to twice the target dose; vi) increase BB up to twice the target dose; vii) increase AA (spironolactone) to 50 mg. Adjustment of loop diuretic does was at the discretion of the investigator. It was not specifically stated if no/any action was taken if the patient was below or at target. Control: Visits same as intervention without BNP data, structured assessment at the discretion of the investigator based on changes in clinical status and/or signs of worsening HF in accordance with ESC guidelines 2001 Intervention provider: Specialist (treating physician experienced in managing patients with HF)	
Outcomes	Review relevant: i) All‐cause mortality; ii) HF mortality; iii) HF admission; iv) All‐cause admission; v) Quality of life Additional outcomes: i) Composite of mortality, need for hospitalisation and worsening HF; ii) Cardiovascular mortality; iii) Cardiovascular hospital admissions; iv) Worsening HF	
Notes		
***Risk of bias***		
**Bias**	**Authors' judgement**	**Support for judgement**
Random sequence generation (selection bias)	Low risk	Block randomisation
Allocation concealment (selection bias)	Low risk	Email by author 21 October 14 "Opaque envelopes"
Blinding of participants and personnel (performance bias) All outcomes	High risk	Unblinded "patients were made aware of their BNP value in order increase motivation to adhere to treatment"
Blinding of outcome assessment (detection bias) All outcomes	Low risk	"All endpoints were adjudicated using a predefined endpoint protocol by a committee with two experienced cardiologists who did not participate in the study and were blinded to the results"
Incomplete outcome data (attrition bias) All outcomes	Unclear risk	Numbers provided, but not reasons
Selective reporting (reporting bias)	Low risk	All outcomes reported as specified in the publication
Other bias	Unclear risk	Source of funding: Swedish Heart‐Lung foundation, Regional research foundation in south eastern Sweden, regional foundation in northern Sweden, and by unrestricted grant from Biosite International and Infiniti Medical AB who supplied BNP analysing equipment

**Table:** Krupicka 2010

Methods	'OPTIMA' Setting: Hospitals in Czech Republic Duration of study: 24 months Inclusion criteria: Newly diagnosed or acutely deteriorating advanced chronic failure (NYHA III‐IV), LVEF ≤ 45% Exclusion criteria: Age under 18 or above 90 years old; acute coronary syndrome during the last three months, pulmonary embolism during the last three months, history of hepatic cirrhosis, severe renal insufficiency (creatinine >250 µmol/L), severe chronic lung disease, current malignant disease.	
Participants	Number of participants at baseline: Intervention 26; Control 26 Gender (male): Intervention 69%; Control 65% Median age (range): Intervention 71 (36‐89); Control 70 (45‐84)	
Interventions	BNP‐guided treatment: minimum two visits in first quarter, five in first year and nine overall ; structured clinical assessment including BNP data at outpatient clinic; treatment intensified according to study algorithm: i) in case of congestion (lung venostasis, peripheral oedema) either daily loop diuretic dose was increased or second diuretic was added, thiazid if creatinine was below 180umol/L; ii) in patients without congestion, ACEi daily dose was increased up to maximal recommended dose. In case of ACEi intolerance, ARB was administered and subsequently titrated; iii) increase of betablocker daily dose up to maximal recommended dose; iv) increase of MRA daily dose up to maximal recommended dose. It was not specifically stated if no/any action was taken if the patient was below or at target. Clincally‐guided treatment (control): Visits same as the intervention group without BNP data, treatment according to standard clinical practice with respect to current Czech guidelines for HF Intervention provider: Specialist	
Outcomes	Review relevant: i) All‐cause mortality; ii) HF mortality; iii) HF admission; iv) Adverse events Additional outcomes: i) Composite of cardiovascular mortality, hospitalisation for worsening HF and outpatient episodes of worsening HF requiring to increase diuretic by at least 50%	
Notes		
***Risk of bias***		
**Bias**	**Authors' judgement**	**Support for judgement**
Random sequence generation (selection bias)	Unclear risk	'randomised'. No description of how achieved
Allocation concealment (selection bias)	Low risk	Email from author 17 October 14 "opaque envelopes"
Blinding of participants and personnel (performance bias) All outcomes	High risk	Email from the author 17 October 14 "Only the patients were blinded to the group allocation"
Blinding of outcome assessment (detection bias) All outcomes	Unclear risk	Not stated
Incomplete outcome data (attrition bias) All outcomes	Unclear risk	Not stated
Selective reporting (reporting bias)	Low risk	All outcomes reported as specified in Krupicka 2010
Other bias	Unclear risk	Source of funding: supported by an educational grant from the ZENTIVA company (ZENTIVA is Czech generic pharmaceutical company)

**Table:** Lainchbury 2010

Methods	'BATTLESCARRED' Setting: Hospital in New Zealand Duration of study: Three years Inclusion criteria: > 18 years old with symptomatic CHF (as defined by Framingham criteria and satisfying ESC guidelines for the diagnosis of HF), requiring admission to hospital and able to give informed consent, pre‐randomisation plasma NTproBNP must exceed 50 pmol/L (i.e. approximately 400 pg/mlL. Recruitment deliberately included elderly patients and patients with a preserved LVEF Exclusion criteria: Active myocarditis/pericarditis, life expectancy due to non‐cardiovascular disease of < 24 months, severe hepatic or pulmonary disease, renal impairment (plasma creatinine > 250 µmol/L), transient HF from myocardial infarction treated with acute revascularisation and a subsequent ejection fraction during the index hospital admission of > 40%, severe valvular disease being considered for surgery, severe aortic stenosis (valve area < 1 cm^2^), HF secondary to mitral stenosis or are under consideration for cardiac transplantation	
Participants	Number of participants at baseline: Intervention 121; Control (CG) 121; Control (UC) 122 Gender (male): Intervention 63%; Control (CG) 67%; Control (UC) 62% Median age (range): Intervention 76 (44 to 89); Control (CG) 76 (34 to 89); Control (UC) 75 (31 to 89)	
Interventions	NT‐proBNP‐guided treatment: minimum two visits in first quarter, five in first year and nine overall ; structured clinical assessment including NT‐proBNP data at outpatient clinic; general education regarding HF; treatment triggered by NT‐proBNP level greater than 150 pmol/L and/or a HF score greater than 2, for values below this threshold, treatment was not altered Algortihm for heart score >2: i) increase frusemide to 120 mg/day or optimisation of ACE inhibitor dose if sub optimal; ii) addition of digoxin 0.25 mg/day adjusted for creatinine clearance; iii) add spironolactone (up to 50 mg/day) in patients with persisting class III or IV symptoms; iv) increase frusemide with twice‐daily doses up to a maximum of 500 mg twice daily with doubling increments; v) addition of bendrofluazide or metolazone Algortihm for NT‐proBNP >150 p/mol, heart score stable: i) optimisation of ACE inhibitor to trial‐based doses; ii) addition or titration of beta blockade to trial‐based doses; iii) addition of further therapy as for the clinically‐guided group Clinically‐guided (CG, control): Visits same as intervention without NT‐proBNP data; treatment determined by HF score above or below 2 Algorithm for heart score < 2: i) optimisation of ACE inhibitor dose; ii) addition and titration or optimisation of beta‐blocker dose Algorithm for heart score > 2: same as NT‐proBNP‐guided treatment Usual care (UC, control): No visit schedule or structured follow‐up; management in primary care with or without requested HF clinic referrals Intervention provider: Specialist (research outpatient clinic) (NT‐proBNP and CG), Primary care physician (UC)	
Outcomes	Review relevant: i) All‐cause mortality; ii) HF admission; iii) Quality of life Additional outcomes: i) Mortality plus episodes of inpatient or outpatient HF decompensation; ii) Mortality plus hospital admission for any cardiovascular event plus episodes of outpatient decompensated HF requiring increased medication treatment for decompensated HF; iii) Episodes of HF decompensation; iv) Episodes of HF decompensation; (v) Changes in NTproBNP, NYHJA status, LVEF, six‐minute walk distance	
Notes		
***Risk of bias***		
**Bias**	**Authors' judgement**	**Support for judgement**
Random sequence generation (selection bias)	Low risk	Stratified by age (≤75 or > 75) in permuted blocks of 30
Allocation concealment (selection bias)	Unclear risk	Not stated
Blinding of participants and personnel (performance bias) All outcomes	Low risk	"double blind", "Patients will be blinded as to their group allocation, and clinical assessments will be made by a physician also blinded. Intensification of drug treatment will be made by an unblinded physician in the research team"
Blinding of outcome assessment (detection bias) All outcomes	Low risk	"double blind"
Incomplete outcome data (attrition bias) All outcomes	Unclear risk	Numbers provided, but not reasons
Selective reporting (reporting bias)	High risk	Planned outcomes specified in protocol. No follow‐up quality of life data for usual care (UC) control group. Analyses for two secondary outcomes were completed and commented on, but data were not provided.
Other bias	Low risk	Source of funding: Grants from the Health Research Council of New Zealand and the National Heart Foundation of New Zealand

**Table:** Li 2015

Methods	Setting: Hospital in China Duration of study: 1 month Inclusion criteria: Moderate to severe HF (NYHA III ‐ IV) Exclusion criteria: Patients with severe renal function damage (serum creatinine > 265 umol/L), bronchial asthma or COPD were excluded, as well as end‐stage HF patients without response to intravenous drug treatment.	
Participants	Number of participants at baseline: Intervention 96; Control 99 Gender (male): Intervention 56.3%; Control 55.4% Average age (range): Intervention 57 (40 to 78); Control 58 (38 to 81)	
Interventions	BNP‐guided treatment: minimum five visits in first month and overall; structured clinical assessment including BNP data; start‐up and use of metoprolol succinate according to BNP level; the BNP level was controlled every 3 to 5 days during the application of intravenous cardiotonic, vasodilator and diuretic; metoprolol succinate treatment triggered if more than 50 % reduction of basal BNP level or BNP < 300 pg/mL. Ongoing dose of metoprolol succinate doubled every visit. If the BNP level did not decrease, but was elevated more than 10% then the metoprolol succinate was stopped or decreased whilst application of intravenous cardiotonic, vasodilator or diuretic drugs took place until start up BNP level achieved then the metoprolol succinate was recommenced Observation group (control): Visits same as intervention group without BNP; structured clinical assessment; start‐up and use of metoprolol succinate according to clinical manifestation; all other HF drugs stopped; after 3 days of stable weight initial dose of 6.25 mg of metoprolol succinate; dose of metoprolol succinate doubled every week until the maximum tolerated dose or target dose if no HF signs and symptoms were observed. Otherwise metoprolol succinate was reduced and intravenous cardiotonic, vasodilator or diuretic was applied until HF signs and symptoms improved and the metoprolol succinate was gradually applied again. Intervention provider: Specialist (highly placed medical profession in cardiology)	
Outcomes	Review relevant: i) HF mortality Additional outcomes: i) Average start up of metoprolol succinate; ii) Maximum dose of metoprolol succinate; iii) Recurrance rate of additional drugs	
Notes		
***Risk of bias***		
**Bias**	**Authors' judgement**	**Support for judgement**
Random sequence generation (selection bias)	Unclear risk	Randomised, but no description of how achieved
Allocation concealment (selection bias)	Unclear risk	Not stated
Blinding of participants and personnel (performance bias) All outcomes	Unclear risk	Not stated
Blinding of outcome assessment (detection bias) All outcomes	Unclear risk	Not stated
Incomplete outcome data (attrition bias) All outcomes	Low risk	Numbers and reasons provided. ".....due to severe bradycardia"
Selective reporting (reporting bias)	Low risk	All outcomes reported as specified in the publication
Other bias	Unclear risk	Source of funding: Not stated

**Table:** Maeder 2013

Methods	'TIME‐CHF (Heart failure preserved LVEF (HFpEF)) Setting: 15 hospital outpatient clinics in Switzerland and Germany Duration of study: 18 months Inclusion criteria: 60 years or older with dyspnoea (NYHA class II with current therapy), a history of hospitalisation for HF within the last year, N‐terminal BNP level of 400 pg/mL or higher in patients younger than 75 years and a level of 800 pg/mL or higher in patients aged 75 years or older, > 45% LVEF Exclusion criteria: patients with dyspnoea not mainly due to HF, with valvular disease requiring surgery, acute coronary syndromes within the previous 10 days, angina pectoris classified as being in the Canadian Cardiovascular Society Class higher than II, revascularisation within the previous month, BMI (calculated as weight in kilograms divided by height in meters squared) higher than 35, serum creatinine level higher than 2.49 mg/dL, a life expectancy of less than 3 years for non cardiovascular diseases, unable to give informed consent, no follow‐up possible, or participating in another study	
Participants	Number of participants at baseline: Intervention 59; Control 64 Gender (male): Intervention 36%; Control 33% Mean age (SD): Intervention 80.3 (6.8); Control 79.9 (7.2)	
Interventions	NT‐proBNP‐guided treatment: minimum three visits in first quarter, five in first year and six or more overall ; structured clinical assessment including NT‐proBNP data, treatment according to recommendations based on previous clinical trials, ESC 2001 and American College of Cardiology and American heart Association guidelines, ongoing trials, pathophysiologic consideration and homogeneity of therapy within the study: i) symptoms and fluid retention are treated with diuretics, all patients should be on an angiotensin II receptor antagonist or ACE inhibitor; ii) if blood pressure is still elevated (i.e. ≥ 140/90 mmHg), a beta blocker should be added. If treatment targets are not reached then the algorithm as for reduced HF patients (Pfisterer 2009) will be used for escalation of treatment: addition of spironolactone, escalating doses of ACE inhibitors, angiotensin II receptor blockers, and ‐blockers, loop diuretics, low‐dose digoxin, long‐acting nitrates, metalozone or another thiazide, molsidomide during nitrate‐free intervals, and intravenous diuretics or inotropes. Therapy was reduced in cases of significant adverse effects, diuretics were recommended to be reduced prior to prognostically relevant medication, all other therapies left to the discretion of the treating physician. Further adjustment of treatment is only completed if criteria for further adjustment are met. Symptom‐guided treatment (control): Visits same as intervention without NT‐proBNP data; pre‐defined escalation rules to reduce symptoms to dyspnoea NYHA class of II or less, all other therapies at discretion of treating physician. Intervention provider: Specialist (HF outpatient clinic with collaboration of general practitioner)	
Outcomes	Review relevant: i) All‐cause mortality; ii) Adverse events; iii) Cost; iv) Qualtiy of life Additional outcomes: i) Survival free of hospitalisation	
Notes	Linked to Pfisterer 2009. Two separate groups of participants in TIME‐CHF	
***Risk of bias***		
**Bias**	**Authors' judgement**	**Support for judgement**
Random sequence generation (selection bias)	Low risk	Stratified by 2 age groups using central allocation in blocks of 8 patients
Allocation concealment (selection bias)	Low risk	"concealed"
Blinding of participants and personnel (performance bias) All outcomes	High risk	"Patients, but not treating physicians, were blinded to group allocation"
Blinding of outcome assessment (detection bias) All outcomes	Unclear risk	Not stated
Incomplete outcome data (attrition bias) All outcomes	Unclear risk	Numbers provided, but not reasons
Selective reporting (reporting bias)	High risk	Planned outcomes specified in Brunner‐LA Rocca 2006. Quality of life outcome not reported
Other bias	Unclear risk	Source of funding: Sponsored by the Horten Research Foundation (Lugano, Switzerland; 55% of the study’s budget), as well as by smaller unrestricted grants from AstraZeneca Pharma, Novartis Pharma, Menarini Pharma, Pfizer Pharma, Servier, Roche Diagnostics, Roche Pharma, and Merck Pharma

**Table:** Persson 2010

Methods	'SIGNAL‐HF' Setting: Community in Sweden Duration of study: Nine months Inclusion criteria: Diagnosis of chronic HF, stable NYHA class II–IV, LVEF 50%, elevated NT‐proBNP levels (males 800, females 1000 ng/L) Exclusion criteria: planned cardiovascular hospitalisation; stroke, acute myocardial infarction, or open heart surgery within the last 3 months before enrolment, mitral stenosis, aortic stenosis of clinical significance, patients already receiving optimal pharmacological treatment for chronic HF according to the national guidelines, serum creatinine ≥265 mmol/L	
Participants	Number of participants at baseline: Intervention 126; Control 124 Gender (male): Intervention 76%; Control 66% Mean age: Intervention 78; Control 77	
Interventions	NT‐proBNP‐guided treatment: minimum four visits in first quarter, six in first year and six overall ; structured clinical assessment including NT‐proBNP data at outpatient clinic, treatment intensified until at least a 50% reduction from baseline NT‐proBNP, stepwise treatment to Swedish guidelines: Patients with NYHA II: base therapy included an ACE‐inhibitor and a betablocker, Loop diuretics could be added and used based on signs of fluid retention. In patients who did not tolerate ACE‐inhibitor treatment, an ARB was to be used instead. Patients with NYHA III–IV: base therapy as for NYHA II, in patients with persistent CHF symptoms despite target or maximum tolerated doses of ACE‐inhibitor and beta‐blocker, additional therapy with an ARB or spironolactone (or eplerenone in the case of hormonal side effects) could be initiated. In addition, digoxin could be added as an option for extra symptom relief, although the main indication for this treatment was atrial fibrillation. Not NT‐proBNP group (control): Visits same as intervention without NT‐proBNP data; same stepwise treatment used based on clinical assessment only It was not specifically stated if no or any action was taken if the patient was below or at target. Intervention provider: Generalist plus 2‐3 hours training about HF guidelines with local cardiologist	
Outcomes	Review relevant: i) All‐cause mortality; ii) Adverse events; iii) Quality of life (not reported) Additional outcomes: i) Composite endpoint of days alive, days out of hospital (for cardiovascular reasons), and symptom score from the Kansas City Cardiomyopathy Questionnaire ii) Change in NT‐proBNP, NYHA, level of titration and intensification of treatment	
Notes		
***Risk of bias***		
**Bias**	**Authors' judgement**	**Support for judgement**
Random sequence generation (selection bias)	Unclear risk	Randomised, but no description of how achieved
Allocation concealment (selection bias)	Unclear risk	Not stated
Blinding of participants and personnel (performance bias) All outcomes	Unclear risk	"single‐blind", lack of details
Blinding of outcome assessment (detection bias) All outcomes	Unclear risk	"single‐blind", lack of details
Incomplete outcome data (attrition bias) All outcomes	Unclear risk	Numbers provided, but not reasons
Selective reporting (reporting bias)	High risk	Planned outcomes specified in Persson 2010. Quality of life outcomes not reported
Other bias	Unclear risk	Source of funding: AstraZeneca

**Table:** Pfisterer 2009

Methods	'TIME‐CHF (Heart failure reduced LVEF (HFrEF)) Setting: 15 hospital outpatient clinics in Switzerland and Germany Duration of study: 18 months Inclusion criteria: 60 years or older with dyspnoea (NYHA class II with current therapy), a history of hospitalisation for HF within the last year, N‐terminal BNP level of 400 pg/mL or higher in patients younger than 75 years and a level of 800 pg/mL or higher in patients aged 75 years or older, ≤ 45% LVEF Exclusion criteria: patients with dyspnoea not mainly due to HF, with valvular disease requiring surgery, acute coronary syndromes within the previous 10 days, angina pectoris classified as being in the Canadian Cardiovascular Society Class higher than II, revascularisation within the previous month, BMI (calculated as weight in kilograms divided by height in meters squared) higher than 35, serum creatinine level higher than 2.49 mg/dL, a life expectancy of less than 3 years for non cardiovascular diseases, unable to give informed consent, no follow‐up possible, or participating in another study	
Participants	Number of participants at baseline: Intervention 251; Control 248 Gender (male): Intervention 68.1%; Control 62.9% Mean age: Intervention 76; Control 77	
Interventions	NT‐proBNP‐guided treatment: minimum three visits in first quarter, five in first year and six or more overall ; structured clinical assessment including NT‐proBNP data, treatment according to ESC 2001 and American College of Cardiology and American heart Association guidelines. Algortihm for escalation of treatment: addition of spironolactone, escalating doses of ACE inhibitors, angiotensin II receptor blockers, and ‐blockers, loop diuretics, low‐dose digoxin, long‐acting nitrates, metalozone or another thiazide, molsidomide during nitrate‐free intervals, and intravenous diuretics or inotropes, therapy was reduced in cases of significant adverse effects, diuretics were recommended to be reduced prior to prognostically‐relevant medication, all other therapies left to the discretion of the treating physician. Further adjustment of treatment is only completed if criteria for further adjustment are met. Symptom‐guided treatment (control): Visits same as intervention without NT‐proBNP data; pre‐defined escalation rules to reduce symptoms to dyspnoea NYHA class of II or less, all other therapies at discretion of treating physician. Intervention provider: Specialist (HF outpatient clinic with collaboration of general practitioner)	
Outcomes	Review relevant: i) All‐cause mortality; ii) Adverse events; iii) Cost; iv) Qualtiy of life Additional outcomes: i) Survival free of hospitalisation	
Notes	Linked to Maeder 2013. Two separate groups of participants in TIME‐CHF	
***Risk of bias***		
**Bias**	**Authors' judgement**	**Support for judgement**
Random sequence generation (selection bias)	Low risk	Stratified by 2 age groups using central allocation in blocks of 8 patients
Allocation concealment (selection bias)	Low risk	"concealed"
Blinding of participants and personnel (performance bias) All outcomes	High risk	"Patients, but not treating physicians, were blinded to group allocation"
Blinding of outcome assessment (detection bias) All outcomes	Unclear risk	Not stated
Incomplete outcome data (attrition bias) All outcomes	Unclear risk	Numbers provided, but not reasons
Selective reporting (reporting bias)	Low risk	Planned outcomes specified in protocol. All outcomes reported.
Other bias	Unclear risk	Source of funding: Sponsored by the Horten Research Foundation (Lugano, Switzerland; 55% of the study’s budget), as well as by smaller unrestricted grants from AstraZeneca Pharma, Novartis Pharma, Menarini Pharma, Pfizer Pharma, Servier, Roche Diagnostics, Roche Pharma, and Merck Pharma

**Table:** Schou 2013

Methods	'NorthStar' Setting: 18 HF clinics in Denmark Duration of study: 30 months Inclusion criteria: > 18 years old, LVEF < 45%, educated in HF disease and management, on optimal medical therapy (ACE inhibitor/ARB, beta‐blocker, aldosterone receptor antagonist) or an implantable cardioverter‐defibrillator and/or CRT, if indicated,and NT‐proBNP ≥ 1000 pg/mL after up‐titration (high‐risk patients were included, but not as target since the patients should receive guideline treatment based on LVEF, functional class, and QRS duration on the ECG before randomisation), euvolaemic and clinically stable according to the pre‐defined stability criteria Exclusion criteria: Plasma creatinine >200 µmol/l200720, waiting for a heart transplant, valvular or Ischaemic heart disease with planned surgery or PCI, withdrawal of ACE inhibitors/ARBs, BB, and ARAs due to a reversible cause of cardiomyopathy, malignancy with life expectancy, 5 years, dementia	
Participants	Number of participants at baseline: Intervention 199; Control 208 Gender (male): Intervention 76%; Control 76% Median age (range): Intervention 72 (56 to 85); Control 74 (51 to 89)	
Interventions	NT‐proBNP‐guided treatment: minimum two visits in first quarter, five in first year and 17 or more overall; structured clinical assessment including NT‐proBNP data, if NT‐proBNP increased to >30% compared with randomisation visit then treatment algorithm triggered (complex algorithm ‐ see article) Clinical management (control): Visits potentially same as intervention without NT‐proBNP data, but at discretion of the investigators; no treatment algorithm, medical treatment controlled at each visit. Intervention provider: Specialist (HF nurse supervised by local cardiologist)	
Outcomes	Review relevant: i) All‐cause mortality; ii) HF admission; iii) All‐cause admission; iv) Quality of life Additional outcomes: i) Composite of all‐cause mortality or admission for a protocol‐specified cardiovascular cause; ii) Cardiovascular hospital admissions; iii) Change in NYHA class and NT‐proBNP levels; iv) Admission days; v) Number of admissions	
Notes		
***Risk of bias***		
**Bias**	**Authors' judgement**	**Support for judgement**
Random sequence generation (selection bias)	Unclear risk	"Randomisation performed". No description of how achieved
Allocation concealment (selection bias)	Unclear risk	"sealed envelopes kept at the local site". Not stated whether opaque
Blinding of participants and personnel (performance bias) All outcomes	High risk	"NT‐proBNP levels are neither blinded for the patients, cardiologists, HFC nurses, or the GPs."
Blinding of outcome assessment (detection bias) All outcomes	Low risk	"vital status and admissions evaluated by an independent endpoint committee whose members were unaware of the study group assignments"
Incomplete outcome data (attrition bias) All outcomes	Low risk	No attrition
Selective reporting (reporting bias)	High risk	Planned outcomes specified in protocol. Cost not reported
Other bias	Unclear risk	Source of funding: Supported by unrestricted grants from Roche Diagnostics International, Schwitzerland; Merck, Sharp and Dohme, Denmark supported development of the electronic case report form; M.S. was supported by a grant from the Copenhagen Hospital Corporation

**Table:** Shah 2011

Methods	'STARBRITE' Setting: Three hospitals in USA Duration of study: Four months Inclusion criteria: LVEF ≤ 35%, NYHA class III/IV on admission, follow‐up in the HF program of each site, and regular access to a telephone Exclusion criteria: Diagnosed with an acute coronary syndrome during the index hospitalisation, serum creatinine level >3.5 mg/dL, required haemodialysis	
Participants	Number of participants at baseline: Intervention 68; Control 69 Gender (male): Intervention 67.7%; Control 72.3% Median age (IQR): Intervention 59 (50,70); Control 63 (52,74)	
Interventions	BNP‐guided treatment: minimum five visits in first quarter, six in first year and overall; structured clinical assessment including BNP data, treatment triggered if BNP increased by more than two times or less than the hospital discharge value of BNP, treatment based on general guidelines and clinician's judgement, telephone follow‐up after visits. Guidelines: i) ≥ target BNP & ≥ target congestion score (CS): Double loop diuretics or add metolazone/HCTZ, check electrolytes and supplement KCl and Mg during visit as needed, ii) ≥ 2x target BNP & < target CS: Double loop diuretics, check electrolytes and supplement KCl and Mg during visit as needed iii) ≥ 2x target BNP & orthostatic hypotension or renal insufficiency: Consider hospital admission if patient unstable and/or has CS 3–5, check electrolytes and supplement KCl and Mg during visit as needed iv) < 2x target BNP & > target CS plus < 2x target BNP & ≤ target CS : Continue current medical regimen v) < 2x target BNP & orthostatic hypotension or renal insufficiency: Consider admission to hospital if patient is unstable, if patient is stable, discontinue thiazide/metolazone; if not taking thiazide/metolazone, reduce daily dose of loop diuretics by half, check electrolytes and supplement KCl and Mg during visit as needed. For all guidelines optimise ACE inhibitors, nitrates, beta‐blockers, spironolactone, and digoxin. Congestion score strategy (control): Visits same as intervention without BNP data; clinical assessment based on congestion score (method to quantify key variables of the clinical assessment, congestion score at hospital discharge used as a target). Guidelines: i) > Target CS: Double loop diuretics or add metolazone/HCTZ, check electrolytes and supplement KCl and Mg during visit as needed; ii) > Target CS & orthostatic hypotension or renal insufficiency: Consider admission to hospital if patient unstable and/or has CS 3–5. If patient is stable and/or has CS 1–2: Discontinue thiazide/metolazone; if patient not taking thiazide/metolazone, reduce daily dose of loop diuretics by half, check electrolytes and supplement KCl and Mg during visit as needed; iii) ≤ Target CS: Continue current medical regimen; iv) ≤ Target CS & orthostatic hypotension or renal insufficiency: Discontinue thiazide/metolazone; if patient not taking thiazide/metolazone, reduce daily dose of loop diuretics by half, check electrolytes and supplement KCl and Mg during visit as needed. For all guidelines optimise ACE inhibitors, nitrates, beta‐blockers, spironolactone, and digoxin. It was not specifically stated if no or any action was taken if the patient was below or at target. Intervention provider: Specialist (HF clinic clinicians, plus HF nurses for follow‐up telephone calls)	
Outcomes	Review relevant: i) All‐cause mortality; ii) All‐cause admission Additional outcomes: i) Survival free of hospitalisation during 90 days; ii) Number of days alive during the study period; iii) Number of diuretic adjustments; iv) Cost (not reported) Trial stopped early due to poor enrolment	
Notes		
***Risk of bias***		
**Bias**	**Authors' judgement**	**Support for judgement**
Random sequence generation (selection bias)	Low risk	"stratified by site with randomisation blocks of 6 through a central telephone centre"
Allocation concealment (selection bias)	Low risk	Email by author 7 October 2014 "opaque envelopes were used"
Blinding of participants and personnel (performance bias) All outcomes	High risk	"Clinicians were aware of the treatment allocation but were blinded to BNP levels in patients in the congestion score strategy arm. Patients were blinded to the randomisation arm."
Blinding of outcome assessment (detection bias) All outcomes	High risk	Email from author 7 October 2014: "No blinding. Outcomes were based on case report forms"
Incomplete outcome data (attrition bias) All outcomes	Low risk	No attrition
Selective reporting (reporting bias)	High risk	Planned outcomes specified in protocol. Cost not reported.
Other bias	Unclear risk	Source of funding: Sponsored by the American Heart Association, the American College of Cardiology/Merck Foundation, and the Duke Clinical Research Institute

**Table:** Shochat 2012

Methods	Setting: Hospital in Israel Duration of study: 16 (±11) months Inclusion criteria: ≥ 18 years old, known chronic HF, HF hospitalisation within last year before recruitment, GFR > 30 ml/mi, signed agreement, NYHA II – IV, NT‐ProBNP >2000 at day of randomisation Exclusion criteria: None	
Participants	Number of participants at baseline: Intervention 60; Control 60 Gender (male): Intervention 88.3%; Control 83% Mean age (SD): Intervention 70.2 (11); Control 69.4 (10.5)	
Interventions	NT‐proBNP‐guided treatment: minimum two visits in first quarter, remainder unclear, visits on average every 45 (SD 19) days; clinical assessment including NT‐proBNP data, treatment intensified if NT‐proBNP higher by more than 30% since last visit and < 2000 pg/mL. Algorrithm (email from author 12 November 14): i) diuretics increased; ii) ACE/ AT1 blocker and/or beta blockers increased. Doses at discretion of clinician Conventional treatment (control): Visit schedule same as NT‐proBNP group, conventionally‐guided treatment without BNP data; No algorithm reported. Intervention provider: Specialist (HF clinic)	
Outcomes	Review relevant: i) All‐cause mortality; ii) HF mortality (data not confirmed); iii) HF admission (data not confirmed); iv) All‐cause admission (data not confirmed) Additional outcomes: i) Cardiovascular mortality	
Notes		
***Risk of bias***		
**Bias**	**Authors' judgement**	**Support for judgement**
Random sequence generation (selection bias)	Low risk	"randomised' by computer"
Allocation concealment (selection bias)	Unclear risk	Email from author 12 November 14 "computer generated".
Blinding of participants and personnel (performance bias) All outcomes	Unclear risk	Email from author 12 November 14 "Patients and physicians blinded to group allocation. Study co‐ordinator not blinded but did not participate in study process". Correspondence with author makes evaluation of bias unclear as it is not known if participants and clinicians were blinded to the monitoring process (intervention).
Blinding of outcome assessment (detection bias) All outcomes	Unclear risk	Not stated
Incomplete outcome data (attrition bias) All outcomes	Unclear risk	Numbers provided, but not reasons
Selective reporting (reporting bias)	Unclear risk	Insufficient information to assess risk
Other bias	Unclear risk	Source of funding: 'Rosh' Company granted sets for NT‐proBNP determination, no additional funding

**Table:** Skvortsov 2015

Methods	Setting: Hospital outpatients in Russia Duration of study: One year Inclusion criteria: Hospital admission due to acute decompensation HF, NYHA class III – IV at admission, LVEF < 40%, high risk at hospital discharge (> 1400 pg/mL NT‐proBNP) Exclusion criteria: Participant unable or unwilling to provide written informed consent, inoperable aortic or mitral valve disease, coronary revascularisation (PCI or CABG) within the previous 3 months, acute myocardial infarction in previous 6 month, inflammatory myocardium disease, serum creatinine > 220 mkmol/mL, severe obstructive or restrictive pulmonary disease, high degree atrioventricular block, alcohol abuse, oncology	
Participants	Number of participants at baseline: Intervention 35; Control 35 Gender (male): Intervention 61%; Control 89% Mean age (SD): Intervention 63.7 (8.6); Control 62.5 (13.3)	
Interventions	NT‐proBNP‐guided treatment: Minimum four visits in first quarter, eight in first year, visits monthly in first six months and then every three months up to one year, structured clinical assessment including NT‐proBNP data, target NP of < 1000 pg/mL pr at least 50% of initial NP measurement at discharge, algorithm for treatment: i) increase in NT‐proBNP, but no clinical deterioration then patients revisited in two weeks. If the trend of increased NT‐proBNP continued without deterioration of clinical symptoms then diuretics were recommended with further visit in 2 weeks (though this may coincide with a scheduled visit); ii) increase in NT‐proBNP with increase in clinical HF symptoms then patients immediately received correction of diuretic therapy; iii) decrease in NT‐proBNP plus increase in clinical symptoms then patients immediately received correction of diuretic therapy (this did effect did not happen in the study), the choice of medications and dose titration was individually determined and continued until the maximum‐tolerated doses of drugs were administered. Standard therapy (control): Minimum four visits in first quarter, eight in first year, visits monthly in first six months and then every three months up to one year, treatment same as intervention group without NT‐proBNP data, treatment adjusted according to ESC and ACCF/AHATF guidelines. Intervention provider: Specialist (HF clinic)	
Outcomes	Review relevant: i) All‐cause mortality; ii) HF mortality; iii) HF admission; iv) Quality of life Additional outcomes: i) Total cardiovascular events; ii) Changes in NT‐proBNP, LVEF, functional capacity i) Cardiovascular events; ii) Cardiovascular mortality; iii) Alternative biomarkers; iv) Clinical and functional status; v) LV systolic and diastolic function; vi) Episodes of HF deterioration needing additional i/v diuretics vii) Blood pressure viii) Serum creatinine ix) Recovery of patients	
Notes		
***Risk of bias***		
**Bias**	**Authors' judgement**	**Support for judgement**
Random sequence generation (selection bias)	Low risk	"randomisation 1:1" using block design, email from author 17.4.16 confirms randomisation by independent investigator
Allocation concealment (selection bias)	Unclear risk	Not stated
Blinding of participants and personnel (performance bias) All outcomes	Unclear risk	Email from author 17 April 16 confirms patients and clinicians blinded to NT‐proBNP measurements in the control group, but unclear if blinded to group allocation
Blinding of outcome assessment (detection bias) All outcomes	High risk	Email from author 17 April 16 confirms outcomes not blinded
Incomplete outcome data (attrition bias) All outcomes	Low risk	Numbers provided with reasons
Selective reporting (reporting bias)	Unclear risk	Planned outcomes specified in Skvortsov 2015. Not all outcomes reported. Email from author 17 April 16 confirmed further publications due shortly
Other bias	Unclear risk	Source of funding: Not stated

**Table:** Troughton 2000

Methods	Setting: Hospital in New Zealand Duration of study: Maximum 17 months Inclusion criteria: Aged 35 to 85, after hospital admission with decompensated HF or from a specialist cardiology outpatient clinic, LVEF < 40%, NYHA class II–IV, treated with ACE inhibitors, loop diuretic with or without digoxin Exclusion criteria: Acute coronary syndrome (within 3 months), pending cardiac transplant or revascularisation, severe stenotic valvular heart disease, or by severe pulmonary (forced expiratory volume in 1 s <1 L) hepatic or renal (plasma creatinine > 0·2 mmol/L) disease	
Participants	Number of participants at baseline: Intervention 33; Control 36 Gender (male): Intervention 78%; Control 75% Mean age: Intervention 68; Control 72	
Interventions	NT‐proBNP‐guided treatment: minimum one visits in first quarter, four in first year, visits two‐weekly until target met and then three‐monthly, structured clinical assessment including NT‐proBNP data, HF score used based on Framingham criteria (score of two or more indicates HF) treatment intensified if BNP target (200 pmol/L) not met.Stepwise increase in therapy: i) maximisation of ACE inhibitors (up to enalapril equivalent of 20 mg twice a day); ii) increase in loop diuretic to furosemide 500 mg twice a day; iii) addition of digoxin up to 0·25 mg/day; additional diuretic (spironolactone 25 mg to 50 mg once a day, then metolazone 2·5 mg to 5 mg once a day) iv) additional vasodilator (isosorbide mononitrate 60 mg to 120 mg once a day then felodipine 2·5 mg to 5 mg once a day) Clinically‐guided treatment (control): minimum one visits in first quarter, two in first year and four overall, treatment same as intervention group without NT‐proBNP data, treatment intensified same as intervention group when triggered by HF score of two or more Intervention provider: Specialist (HF clinic)	
Outcomes	Review relevant: i) All‐cause mortality; ii) HF mortality; iii) HF admission; iv) All‐cause admission; v) Adverse events; vi) Qualtiy of life (no Additional outcomes: i) Total cardiovascular events; ii) Changes in NT‐proBNP, LVEF, functional capacity	
Notes		
***Risk of bias***		
**Bias**	**Authors' judgement**	**Support for judgement**
Random sequence generation (selection bias)	Low risk	"randomised" by computer. Email from author 21 October 2014 "Computer generated randomisation schedule".
Allocation concealment (selection bias)	Unclear risk	Not stated
Blinding of participants and personnel (performance bias) All outcomes	High risk	Investigator intensifying treatment aware of group allocations
Blinding of outcome assessment (detection bias) All outcomes	Unclear risk	Not stated
Incomplete outcome data (attrition bias) All outcomes	Low risk	No attrition.
Selective reporting (reporting bias)	Low risk	Planned outcomes specified in Troughton 2000. All outcomes reported
Other bias	Unclear risk	Source of funding: grants from Health Research Council of New Zealand and Lottery Health

ACE: angiotensin‐converting enzyme ACEi: angiotensin‐converting enzyme inhibitor ARB: angiotensin receptor blocker BMI: body mass index BNP: brain natriuretic peptide or b‐type natriuretic peptide CABG: coronary artery bypass graft CHF: chronic heart failure CAPD: continuous ambulatory peritoneal dialysis COPD: chronic obstructive pulmonary disease CRT: cardiac resynchronisation therapy ECG: electrocardiogram ESC: European Society of Cardiology FEV1: forced expiratory volume GFR: glomerular filtration rate HF: heart failure KCL: potassium chloride LVEF: left ventricular ejection fraction Mg: magnesium MRA: mineralocorticoid receptor antagonists NT‐proBNP: N‐terminal pro b‐type natriuretic peptide NYHA: New York Heart Association PCI: percutaneous coronary intervention SD: standard deviation [STEMI: segment elevation myocardial infarction} /d: per day

### Characteristics of excluded studies [ordered by study ID]

**Table:**

Study	Reason for exclusion
Brunner‐La Rocca 2015	Not RCT. Further analysis from Troughton 2014 individual patient data meta analysis
ChiCTR‐TRC‐08000284	Not NP‐guided treatment
Cocco 2015	Not RCT
Dandamudi 2012	Not RCT
De Vecchis 2013	Not RCT
Di Somma 2008	Not RCT
Dong 2014	Not RCT
El‐Muayed 2004	Not RCT
Felker 2006	Not RCT
Gaggin 2013	Not RCT
Gonzalez 2012	Not RCT
Green 2009	Not RCT
Jernberg 2003	Not treatment for heart failure
Kociol 2011	Not NP‐guided treatment
Koitabashi 2005	Not RCT
Komajda 2006	Not NP‐guided treatment
Krackhardt 2008	Not RCT
Krackhardt 2011	Not RCT
Ledwidge 2013	Not heart failure population
Leuchte 2005	Not RCT
Li 2007	Not NP‐guided treatment
Lindahl 2005	Not NP‐guided treatment
Luchner 2012	Not NP‐guided treatment
Maisel 2013	Not RCT
McNairy 2002	Not RCT
Miller 2009	Not RCT
Murdoch 1999	No prespecified outcomes
NCT00206856	Trial terminated
NCT00622531	Trial terminated
NCT01299350	Not NP‐guided treatment
Pascual‐Figal 2008	Not RCT
Tang 2005	Not RCT
Troughton 2004	Not RCT
Valle 2008	Not RCT
Wasywich 2009	Not RCT

RCT: randomised controlled trial

### Characteristics of ongoing studies [ordered by study ID]

**Table:** Felker 2014

Trial name or title	NCT01685840 'GUIDE‐IT'
Methods	Setting: USA & Canada Duration of study: 12‐24 months Inclusion criteria: ≥18 years old, LVEF ≤ 40% within 12 months of randomisation, High risk HF (HF hospitalisation, treatment in emergency department, outpatient treatment with intravenous diuretics in the prior 12 months) AND NT‐proBNP greater than 2000 pg/mL or BNP greater than 400 pg/mL at any time during the 30 days prior to randomisation, willing to provide informed consent Exclusion criteria: Acute coronary syndrome or cardiac revascularisation procedure within 30 days, cardiac resynchronisation therapy (CRT) within prior 3 months or current plan to implant CRT device, active myocarditis, hypertrophic obstructive cardiomyopathy, pericarditis, or restrictive cardiomyopathy, severe stenotic valvular disease, anticipated heart transplantation or ventricular assist device within 12 months, chronic inotropic therapy, complex congenital heart disease, end stage renal disease with renal replacement therapy, non cardiac terminal illness with expected survival less than 12 months, women who are pregnant or planning to become pregnant, inability to comply with planned study procedures, enrolment or planned enrolment in another clinical trial
Participants	Number of participants at baseline: 1100 (all groups)
Interventions	NT‐proBNP‐guided treatment: Visits every two weeks until optimal doses of therapies achieved, then every three months. Titration of HF treatment using guideline recommended therapies with a target of achieving and maintaining NT‐proBNP level <1000 pg/mL Usual care: Visit schedule same as for first arm. Ttitration of HF treatment based on target doses of evidence‐based guidelines (American Heart Association and American College of Cardiology) Intervention provider: Treating physician for all arms
Outcomes	Review relevant: i) quality of life; ii) adverse events; iii) medical costs, resource and cost‐effectiveness Additional outcomes: i) time to cardiovascular death or HF hospitalisation; ii) time to all‐cause mortality and cardiovascular mortality; iii) cumulative morbidity; iv) time to first HF hospitalisation
Starting date	December 2012
Contact information	gayle.e.paynter@duke.edu michael.felker@duke.edu
Notes	Unblinded. Except blinded clinical committee to adjudicate all deaths and hospitalisations Analysis on intention‐to‐treat basis Due to finish in December 2017

**Table:** Jourdain 2014

Trial name or title	NCT02110433
Methods	Setting: Hospitals in France Duration of study: 12 months Inclusion criteria: > 18 years old, HF diagnosed on a first hospitalisation for acute exacerbation during the last 12 months, without high age limit, minimal knowledge of the French language (patient or his relatives), informed written consent, resides or is treated in Ile de France, insured under the social security system Exclusion criteria: Myocardial infarction or revascularisation or heart valve surgery < 3 months, inability to execute the feasibility test, major cognitive disorders do not allow access to the platform, patient does not have the necessary autonomy to use the equipment, patient enrolled in another clinical trial, renal failure with creatininemia clearance (cockcroft) <15 mL/min 24h/day oxygen
Participants	Number of participants at baseline: 330 (all groups)
Interventions	BNP‐guided treatment plus Cordiva system: Cordiva system plus BNP home monitoring (weekly) Cordiva system (tele monitoring system): scheduled visit with cardiologist every three months, monthly phone contact, daily questions via Cordiva system (eight questions for decompensation and body weight) Placebo (control): unlimited visits, managed according to ESC guidelines
Outcomes	Review relevant: i) all‐cause mortality; ii) HF admission; iii) quality of life; vi) cost Additional outcomes: i) composite end point including unplanned hospitalisations for CHF with hospital stay > 1 day / all‐cause death/ non‐programmed emergency department admission related to CHF; ii) emergency admission; iii) adherence to strategy; iv) false positive induced by the system; v) false positive induced by the system
Starting date	December 2013
Contact information	patrick.jourdain@ch‐pontoise.fr, maryline.delattre@ch‐pontoise.fr
Notes	Due to finish in December 2015

**Table:** Metra 2012

Trial name or title	
Methods	Setting: Italy
Participants	Number of participants at baseline: 300 (all groups)
Interventions	BNP‐guided treatment Control
Outcomes	
Starting date	January 2005
Contact information	metramarco@libero.it
Notes	Recrutiment finished in August 2009 Currently in write up

**Table:** Moe 2007

Trial name or title	EX‐IMPROVE‐CHF (NCT00601679)
Methods	Setting: Three hospitals in Canada Duration of study: 24 months Inclusion criteria: ≥ 18 years old, NYHA class II‐IV, followed in a programmed HF management setting Exclusion criteria: Life expectancy <1 year due to causes other than HF such as advanced cancer, any other conditions that may render the patient ineligible according to the investigator's judgment
Participants	Number of participants at baseline: 400 (all groups)
Interventions	NT‐proBNP‐guided treatment: minimum two visits in first quarter, five in first year, surveillance NT‐proBNP levels disclosed to physicians Usual care (control): minimum two visits in first quarter, five in first year, no intervention, surveillance NT‐proBNP levels blinded Intervention provider: HF clinic specialists
Outcomes	Review relevant: i) All‐cause mortality Additional outcomes: i) HF hospitalisation and death; ii) time to hospitalisation/admission to emergency department due to HF; iii) total number of HF events; iv) total number of hospitalisations for cardiovascular events; v) cardiovascular mortality; vi) worsening in clinical status but not requiring hospital admission
Starting date	December 2007
Contact information	moeg@smh.ca fernandoc@smh.ca
Notes	Due to finish in December 2014

**Table:** Saraya 2015

Trial name or title	
Methods	Setting: Hospital in Eygpt Duration of study: Six months Inclusion criteria: Patients with HF and reduced ejection fraction Exclusion criteria: acute or chronic renal failure, chronic lung disease, massive pericardial effusion, acute coronary syndrome
Participants	Number of participants at baseline: Intervention 25; Control 25 (2 further groups: ultrasound lung comets [n = 25], Doppler imaging [n = 25])
Interventions	BNP‐guided treatment: Plus clinical findings, point of care device for BNP, target level below 200 pg/mL Clinical findings alone (control) Ultrasound lung comets: Plus clinical findings, targeting a score below 15 Doppler imaging: Plus clinical findings, targeting a mean below 10 E/E
Outcomes	Review relevant: i) HF admission
Starting date	July 2012
Contact information	Not stated
Notes	Finished August 2014 Limited data in the conference abstract, awaiting full publication Source of funding: Eygptian Society of Cardiology

**Table:** Steinen 2014

Trial name or title	PRIMA II (NTR3279)
Methods	Setting: Hospitals in the Netherlands Duration of study: Six months Inclusion criteria: Acute decompensated HF (either de novo or acute‐on‐chronic HF) and NT‐proBNP levels of N1,700 ng/L (ie, 200 pmol/L) measured within 24 hours of hospital admission Exclusion criteria: COPD with FEV1 of <1 L, pulmonary embolism within 1 month before admission and pulmonary hypertension not caused by left ventricle dysfunction, undergoing CAPD/haemodialysis patients, planned coronary artery bypass graft (CABG), percutaneous coronary intervention (PCI), cardiac resynchronisation therapy (CRT), and/or valvular surgery before randomisation, cardiogenic shock at admission requiring invasive treatment, history of STEMI, CABG, PCI, CRTand/or valvular surgery within 1 month before admission, signed informed consent for any current interventional study, presence of severe noncardiac‐related life‐threatening disease before inclusion with an expected survival of < 6 months after inclusion, unwillingness to give or mental or physical status not allowing written informed consent, circumstances that prevent follow‐up (no permanent home address, transient, etc)
Participants	Number of participants at baseline: Intervention 170; Control 170
Interventions	NT‐proBNP‐guided treatment: minimum three plus visits in first quarter, four plus in first year, four plus visits overall, structured clinical assessment including NT‐proBNP data in hospital, when patients achieve over 30% reduction in NT‐proBNP values hospital discharge and follow‐up occurs. Under 30% NT‐proBNP measurements triggers a drug algorithm: For patients with reduced ejection fractions: i) up‐titration or addition of ACE inhibitor, β‐blocker, and/or aldosterone antagonist; ii) CRT for patients who meet current guideline criteria; iii) electrical cardioversion for new‐onset atrial fibrillation; iv) coronary artery angiography (CAG) or intervention when ischemia is suspected. For patients with preserved ejection fractions: i) adequately treat hypertension and myocardial ischaemia; ii) ventricular rate control in atrial fibrillation; iii) electrical cardioversion for new‐onset atrial fibrillation; iv) CAG or intervention when ischaemia is suspected Conventional therapy (control): Discharge and follow‐up of the patients can be planned at the discretion of the treating physician, physicians are discouraged from taking NT‐proBNP measurements Intervention provider: Physicians (control), HF nurses/cardiologists (intervention)
Outcomes	Review relevant: i) all‐cause mortality; ii) HF admission; iii) cost; iv) quality of life Additional outcomes: i) composite all‐cause mortality and HF hospitalisations; ii) hospital free survival in the first 180 days
Starting date	November 2011
Contact information	w.e.kok@amc.uva.nl
Notes	Due to finish in December 2014 Source of funding: Netherlands Heart Foundation, Dutch Organization for Scientific Research (NWO), the Royal Dutch Academy of Arts and Sciences (KNAW) – Interuniversity Cardiology Institute of the Netherlands, Pfizer, Astra‐Zeneca, Medtronic, and Roche Diagnostics

ACE: angiotensin‐converting enzyme CHF: chronic heart failure CAPD: continuous ambulatory peritoneal dialysis COPD: chronic obstructive pulmonary disease ESC: European Society of Cardiology FEV1: forced expiratory volume HF: heart failure LVEF: left ventricular ejection fraction NYHA: New York Heart Association STEMI: segment elevation myocardial infarction

## Differences between protocol and review

The search strategies in the final review differ slightly from those published in the protocol. Since the original protocol Cochrane updated the filter for Embase, which introduced terms making the search more specific for trial design. The current search reflects these updates.

Post hoc subgroup analyses were considered for baseline left ventricular ejection fraction (LVEF), control type and duration of follow‐up. LVEF was considered after extraction of data from the studies when it was identified that LVEF frequently formed one of the inclusion/exclusion criteria for participants and was usually recorded in the baseline characteristics of participants in studies. It was not anticipated that there could be more than one type of control group in the original protocol. Finally, most included studies had a follow‐up period of one to two years, only two studies monitored for a longer period and only two concentrated on up‐titration of heart failure drug(s). Similarly, this had not been anticipated in the original protocol. We wanted to assess if studies subgrouped by either of these aspects could lead to further understanding of NP‐guided treatment.

Post hoc, in response to peer reviewer comments, we completed a sensitivity analysis for all outcomes to evaluate the impact of any differences between the two biomarkers: BNP and NT‐proBNP.

Whilst not pre‐specified in the protocol, a 'Summary of findings' table and GRADE assessment were completed. These now form a mandatory, and desirable, part of the Cochrane review process.

## References

[CD008966-bib-0001] Anguita M, Esteban F, Castillo JC, Mazuelos F, Lopez‐Granados A, Arizon JM, et al. Usefulness of brain natriuretic peptide levels, as compared with usual clinical control, for the treatment monitoring of patients with heart failure. Medicina Clinica 2010;135(10):435‐40. 10.1016/j.medcli.2009.11.048 20673678

[CD008966-bib-0002] Beck‐da‐Silva L, Bold A de, Fraser M, Williams K, Haddad H. BNP‐guided therapy not better than expert's clinical assessment for beta‐blocker titration in patients with heart failure. Congestive Heart Failure 2005;11(5):248‐53. 10.1111/j.1527-5299.2005.04239.x 16230866

[CD008966-bib-0003] Adlbrecht C, Huelsmann M, Berger R, Moertl D, Strunk G, Oesterle A, et al. Cost analysis and cost‐effectiveness of NT‐proBNP‐guided heart failure specialist care in addition to home‐based nurse care. European Journal of Clinical Investigation 2011;41(3):315‐22. 10.1111/j.1365-2362.2010.02412.x 21070222

[CD008966-bib-0004] Berger R, Moertl D, Peter S, Ahmadi R, Huelsmann M, Yamuti S, et al. N‐terminal pro‐B‐type natriuretic peptide‐guided, intensive patient management in addition to multidisciplinary care in chronic heart failure a 3‐arm, prospective, randomised pilot study. Journal of the American College of Cardiology 2010;55(7):645‐53. 10.1016/j.jacc.2009.08.078 20170790

[CD008966-bib-0005] Eurlings LW, Sanders‐van Wijk S, Kraaij DJW van, Kimmenade R van, Meeder JG, Kamp O, et al. Risk stratification with the use of serial N‐terminal pro‐B‐type natriuretic peptide measurements during admission and early after discharge in heart failure patients: post hoc analysis of the PRIMA study. Journal of Cardiac Failure 2014;20(12):881‐90. 10.1016/j.cardfail.2014.08.014 25175696

[CD008966-bib-0006] Eurlings LWM, Pol PEJ van, Kok WE, Wijk S van, Lodewijks‐van der Bolt C, Balk AHMM, et al. Management of chronic heart failure guided by individual N‐terminal pro‐B‐type natriuretic peptide targets: results of the PRIMA (Can PRo‐brain‐natriuretic peptide guided therapy of chronic heart failure IMprove heart fAilure morbidity and mortality?) study. Journal of the American College of Cardiology 2010;56(25):2090‐100. 10.1016/j.jacc.2010.07.030 21144969

[CD008966-bib-0007] Bhardwaj A, Rehman SU, Mohammed AA, Gaggin HK, Barajas L, Barajas J, et al. Quality of life and chronic heart failure therapy guided by natriuretic peptides: results from the ProBNP Outpatient Tailored Chronic Heart Failure Therapy (PROTECT) study. American Heart Journal 2012;164(5):793‐99. 10.1016/j.ahj.2012.08.015 23137512

[CD008966-bib-0008] Bhardwaj A, Rehman SU, Mohammed AA, Han‐na K, Barajas L, Barajas J, et al. NT‐ProBNP guided therapy improves the quality of life in patients with chronic heart failure. Results from the ProBNP outpatient tailored chronic heart failure therapy study. Circulation. 2011; Vol. 124, issue 21 Suppl.1:A13596.

[CD008966-bib-0009] Bhardwaj A, Rehman SU, Mohammed AA, Kim HN, Barajas L, Barajas J, et al. NT‐proBNP guided therapy improves the quality of life in patients with chronic heart failure. Results from the ProBNP outpatient tailored chronic heart failure therapy study. Journal of Cardiac Failure 2011;17(8 Suppl 1):S94‐5.

[CD008966-bib-0010] Bhardwaj A, Rehman SU, Mohammed Asim, Baggish AL, Moore SA, Januzzi JL. Design and methods of the Pro‐B Type Natriuretic Peptide Outpatient Tailored Chronic Heart Failure Therapy (PROTECT) Study. American Heart Journal 2010;159(4):532‐8. 10.1016/j.ahj.2010.01.005 20362709

[CD008966-bib-0011] Dudzinski DM, Gaggin HK, Belcher A, Berardinis B De, He W, Januzzi JL. NT‐proBNP Guided outpatient management of systolic heart failure is cost‐saving. Circulation. 2012; Vol. 126, issue 21 SUPPL.1:A13263.

[CD008966-bib-0012] Gaggin HK, Mohammed AA, Bhardwaj A, Rehman SU, Gregory SA, Weiner RB, et al. Heart failure outcomes and benefits of NT‐proBNP‐guided management in the elderly: results from the prospective, randomised ProBNP outpatient tailored chronic heart failure therapy (PROTECT) study. Journal of Cardiac Failure 2012;18(8):626‐34. 10.1016/j.cardfail.2012.05.005 22858078

[CD008966-bib-0013] Gandhi PU, Szymonifka J, Motiwala SR, Belcher AM, Januzzi JL, Gaggin HK. Characterization and prediction of adverse events from intensive chronic heart failure management and effect on quality of life: results from the pro‐B‐type natriuretic peptide outpatient‐tailored chronic heart failure therapy (PROTECT) study. Journal of Cardiac Failure 2014;21(1):9‐15. 10.1016/j.cardfail.2014.10.008 25463415

[CD008966-bib-0014] Januzzi JL, Rehman SU, Mohammed AA, Bhardwaj A, Barajas L, Barajas J, et al. Use of amino‐terminal pro‐B‐type natriuretic peptide to guide outpatient therapy of patients with chronic left ventricular systolic dysfunction. Journal of the American College of Cardiology 2011;58(18):1881‐9. 10.1016/j.jacc.2011.03.072 22018299

[CD008966-bib-0015] Mallick A, Gandhi PU, Gaggin HK, Januzzi JL. "Worsening heart failure" in chronic heart failure with reduced ejection fraction: Definition, characteristics, and effects of NT‐proBNP guided therapy. Circulation. 2015; Vol. 132.

[CD008966-bib-0016] NCT00351390. The use of Pro‐Brain Natriuretic Peptide targeted therapy to tailor medical management of patients with congestive heart failure followed in an outpatient setting: the ProBNP Outpatient Tailored CHF Therapy (PROTECT) Study. http://clinicaltrials.gov/ct2/show/NCT00351390 (accessed 25 June 2010).

[CD008966-bib-0017] Weiner RB, Baggish AL, Chen‐Tournoux A, Marshall JE, Gaggin HK, Bhardwaj A, et al. Improvement in structural and functional echocardiographic parameters during chronic heart failure therapy guided by natriuretic peptides: mechanistic insights from the ProBNP Outpatient Tailored Chronic Heart Failure (PROTECT) study. European Journal of Heart Failure 2013;15(3):342‐51. 10.1093/eurjhf/hfs180 23132825

[CD008966-bib-0018] Weiner RB, Baggish AL, Chen‐Tournoux AA, Marshall JE, Kim HN, Bhardwaj A, et al. Improvement of echocardiographic parameters associated with NT‐proBNP guided heart failure management: Mechanistic insights from the proBNP outpatient tailored chronic heart failure (protect) study. Journal of the American College of Cardiology 2011;Conference: 60th Annual Scientific Session of the American College of Cardiology and i2 Summit: Innovation in Intervention, ACC.11 New Orleans, LA United States. Conference Start: 20110402 Conference End: 20110405. Conference Publication:(57 (14 SUPPL. 1)):E2030.

[CD008966-bib-0019] Jourdain P, Jondeau G, Funck F, Gueffet P, Helloco A Le, Donal E, et al. Plasma brain natriuretic peptide‐guided therapy to improve outcome in heart failure: the STARS‐BNP Multicenter Study. Journal of the American College of Cardiology 2007;49(16):1733‐9. 10.1016/j.jacc.2006.10.081 17448376

[CD008966-bib-0020] Karlstrom P, Alehagenm U, Boman K, Dahlstrom U, U.PSTEP‐study group. Brain natriuretic peptide‐guided treatment does not improve morbidity and mortality in extensively treated patients with chronic heart failure: responders to treatment have a significantly better outcome.[Erratum appears in Eur J Heart Fail. 2012 May;14(5):563 Note: von den Luederer Tomas [corrected to von Lueder, Thomas G]]. European Journal of Heart Failure 2011;13(10):1096‐103. 10.1093/eurjhf/hfr078 21715446

[CD008966-bib-0021] Karlstrom P, Dahlstrom U, Boman K, Alehagen U. Responder to BNP‐guided treatment in heart failure. The process of defining a responder Results from the Use of PeptideS in Tailoring hEart failure Project or UPSTEP study. Scandinavian Cardiovascular Journal 2015;49(6):316‐24. 10.3109/14017431.2015.1070961 26153427

[CD008966-bib-0022] Karlstrom P, Johansson P, Dahlstrom U, Boman K, Alehagen U. Can BNP‐guided therapy improve health‐related quality of life, and do responders to BNP‐guided heart failure treatment have improved health‐related quality of life? Results from the UPSTEP study. BMC Cardiovascular Disorders 2016;16(1):39. 10.1186/s12872-016-0221-7 PMC4763442 26905220

[CD008966-bib-0023] Hradec J, Krupicka J, Janota T. [Will the therapy of chronic heart failure be guided by plasma levels of natriuretic peptides?]. Casopis Lekaru Ceskych 2009;148(8):383‐8. 19899725

[CD008966-bib-0024] Krupicka J, Janota T, Hradec J. Optimalization of heart failure therapy guided by plasma BNP concentrations. European Society of Cardiology 2010;Conference: European Society of Cardiology, ESC Congress 2010 Stockholm Sweden. Conference Start: 20100828 Conference End: 20100901. Conference Publication: (var.pagings). 31:859‐60.

[CD008966-bib-0025] Krupicka J, Janota T, Kasalova Z, Hradec J. Natriuretic peptides ‐ physiology, pathophysiology and clinical use in heart failure. Physiological Research 2009;58(2):171‐7. 10.33549/physiolres.931461 18380534

[CD008966-bib-0026] Lainchbury JG, Troughton R, Strangman KM, Frampton CM, Pilbrow A, Yandle TG, et al. N‐terminal pro‐B‐type natriuretic peptide‐guided treatment for chronic heart failure: results from the BATTLESCARRED (NT‐proBNP‐Assisted Treatment To Lessen Serial Cardiac Readmissions and Death) trial. Journal of the American College of Cardiology 2010;55(1):53‐60. 10.1016/j.jacc.2009.02.095 20117364

[CD008966-bib-0027] Lainchbury JG, Troughton RW, Frampton CM, Yandle TG, Hamid A, Nicholls M, et al. NTproBNP‐guided drug treatment for chronic heart failure: design and methods in the "BATTLESCARRED" trial. European Journal of Heart Failure 2006;8(5):532‐8. 10.1016/j.ejheart.2006.04.004 16829189

[CD008966-bib-0028] Li JJ, Xiang XL, Tian XY, Shi YF. Clinical research on brain natriuretic peptide guiding the application of beta1 receptor blocker in patients with moderate to severe heart failure. Acta Cardiologica Sinica 2015;31(1):52‐8. 10.6515/ACS20140728A PMC4804913 27122846

[CD008966-bib-0029] Brunner‐La Rocca HP, Buser PT, Schindler R, Bernheim A, Rickenbacher P, Pfisterer M, et al. Management of elderly patients with congestive heart failure‐‐design of the Trial of Intensified versus standard Medical therapy in Elderly patients with Congestive Heart Failure (TIME‐CHF). American Heart Journal 2006;151(5):949‐55. 10.1016/j.ahj.2005.10.022 16644310

[CD008966-bib-0030] Brunner‐La Rocca HP, Maeder MT, Muzzarelli S, Rickenbacher P, Gutmann M, Jeker U, et al. Does response to therapy differ between preserved and reduced LV systolic function in heart failure? Results from TIME‐CHF. European Journal of Heart Failure, Supplement 2010;Conference: Heart Failure 2010 Congress Berlin Germany. Conference Start: 20100529 Conference End: 20100601. Conference Publication: (var.pagings). 9:S116.

[CD008966-bib-0031] Kaufmann BA, Min SY, Goetschalckx K, Bernheim AM, Buser PT, Pfisterer ME, et al. How reliable are left ventricular ejection fraction cut offs assessed by echocardiography for clinical decision making in patients with heart failure?. International Journal of Cardiovascular Imaging 2013;29(3):581‐8. 10.1007/s10554-012-0122-5 22965859

[CD008966-bib-0032] Maeder MT, Rickenbacher P, Rickli H, Abbu H, Gutmann M, Erne P, et al. N‐terminal pro brain natriuretic peptide‐guided management in patients with heart failure and preserved ejection fraction: findings from the Trial of Intensified versus standard Medical therapy in Elderly patients with Congestive Heart Failure (TIME‐CHF). European Journal of Heart Failure 2013;15:1148‐56. 10.1093/eurjhf/hft076 23657728

[CD008966-bib-0033] Maeder MT, Rickli H, Pfisterer ME, Muzzarelli S, Ammann P, Fehr T, et al. Incidence, clinical predictors, and prognostic impact of worsening renal function in elderly patients with chronic heart failure on intensive medical therapy. American Heart Journal 2012;163(3):407‐14. 10.1016/j.ahj.2011.12.003 22424011

[CD008966-bib-0034] Muzzarelli S, Maeder MT, Toggweiler S, Rickli H, Nietlispach F, Julius B, et al. Frequency and predictors of hyperkalemia in patients >60 years of age with heart failure undergoing intense medical therapy. American Journal of Cardiology 2012;109(5):693‐8. 10.1016/j.amjcard.2011.10.027 22152974

[CD008966-bib-0035] Peeters JM, Sanders‐van Wijk S, Bektas S, Knackstedt C, Rickenbacher P, Nietlispach F, et al. Biomarkers in outpatient heart failure management; are they correlated to and do they influence clinical judgment?. Netherlands Heart Journal 2014;22(3):115‐21. 10.1007/s12471-013-0503-y PMC3931853 24338787

[CD008966-bib-0036] Rickenbacher P, Pfisterer M, Burkard T, Kiowski W, Follath F, Burckhardt D, et al. Baseline characteristics, adverse events and hospitalizations indicate an increased risk of death in patients with heart failure. An analysis of the TIME‐CHF trial. European Heart Journal 2011;Conference: European Society of Cardiology, ESC Congress 2011 Paris France. Conference Start: 20110827 Conference End: 20110831. Conference Publication: (var.pagings). 32:125‐6.

[CD008966-bib-0037] Rickenbacher P, Pfisterer M, Burkard T, Kiowski W, Follath F, Burckhardt D, et al. Why and how do patients with heart failure die? Insights from the TIME‐CHF trial. European Heart Journal 2011;Conference: European Society of Cardiology, ESC Congress 2011 Paris France. Conference Start: 20110827 Conference End: 20110831. Conference Publication: (var.pagings). 32:665.

[CD008966-bib-0038] Sanders‐van Wijk S, Empel V van, Davarzani N, Maeder MT, Handschin R, Pfisterer ME, et al. TIME‐CHF investigators. Circulating biomarkers of distinct pathophysiological pathways in heart failure with preserved vs. reduced left ventricular ejection fraction. European Journal of Heart Failure 2015;17(10):1006‐14. 10.1002/ejhf.414 26472682

[CD008966-bib-0039] Wijk S Van, Asselt T Van, Maeder MT, Muzzarelli S, Erne P, Estlinbaum W, et al. Cost‐effectiveness of NT‐proBNP‐guided therapy in heart failure; results from the TIME‐CHF study. European Heart Journal 2011;Conference: European Society of Cardiology, ESC Congress 2011 Paris France. Conference Start: 20110827 Conference End: 20110831. Conference Publication: (var.pagings). 32:161.

[CD008966-bib-0040] Zurek M, Maeder MT, Rickli H, Muzzarelli S, Sanders‐van Wijk S, Abbuhl H, et al. Differential prognostic impact of resting heart rate in older compared with younger patients with chronic heart failure‐‐insights from TIME‐CHF. Journal of Cardiac Failure 2015;21(4):347‐54. 10.1016/j.cardfail.2014.12.014 25576682

[CD008966-bib-0041] Drexler B, Mueller C. Natriuretic peptide‐guided management by the general practitioner: how to interpret the SIGNAL. European Journal of Heart Failure 2010;12(12):1265‐7. 10.1093/eurjhf/hfq202 21098577

[CD008966-bib-0042] Erntell H, Swedberg K, Jorgensen L, Dahlstrom U, Persson H. Predictors of NT‐proBNP response in primary care patients with heart failure and NT‐proBNP guided therapy. European Journal of Heart Failure 2013;Conference: Heart Failure Congress 2013 Lisbon Portugal. Conference Start: 20130525 Conference End: 20130528. Conference Publication: (var.pagings). 12:S31.

[CD008966-bib-0043] NCT00391846. A single blind, multicentre, 9‐month, phase IV study, comparing treatment guided by clinical symptoms and signs and NT‐proBNP vs treatment guided by clinical symptoms and signs alone, in patients with heart failure (HF) and left ventricular systolic dysfunction. http://onlinelibrary.wiley.com/o/cochrane/clcentral/articles/961/CN‐00961961/frame.html 2006.

[CD008966-bib-0044] Persson H, Erntell H, Eriksson B, Johansson G, Swedberg K, Dahlstrom U. Improved pharmacological therapy of chronic heart failure in primary care: a randomized Study of NT‐proBNP Guided Management of Heart Failure‐‐SIGNAL‐HF (Swedish Intervention study‐‐Guidelines and NT‐proBNP AnaLysis in Heart Failure). European Journal of Heart Failure 2010;12(12):1300‐8. 10.1093/eurjhf/hfq169 20876734

[CD008966-bib-0045] Brunner‐La Rocca HP, Buser PT, Schindler R, Bernheim A, Rickenbacher P, Pfisterer M, et al. Management of elderly patients with congestive heart failure‐‐design of the Trial of Intensified versus standard Medical therapy in Elderly patients with Congestive Heart Failure (TIME‐CHF). American Heart Journal 2006;151(5):949‐55. 10.1016/j.ahj.2005.10.022 16644310

[CD008966-bib-0046] Brunner‐La Rocca HP, Knackstedt C, Eurlings L, Rolny V, Krause F, Pfisterer ME, et al. Impact of worsening renal function related to medication in heart failure. European Journal of Heart Failure 2015;17(2):159‐68. 10.1002/ejhf.210 25808849

[CD008966-bib-0047] Brunner‐La Rocca HP, Maeder MT, Muzzarelli S, Rickenbacher P, Gutmann M, Jeker U, et al. Does response to therapy differ between preserved and reduced LV systolic function in heart failure? Results from TIME‐CHF. European Journal of Heart Failure, Supplement 2010;Conference: Heart Failure 2010 Congress Berlin Germany. Conference Start: 20100529 Conference End: 20100601. Conference Publication: (var.pagings). 9:S116.

[CD008966-bib-0048] Kaufmann BA, Goetschalckx K, Min SY, Maeder MT, Bucher U, Nietlispach F, et al. TIME‐CHF investigators. Improvement in left ventricular ejection fraction and reverse remodeling in elderly heart failure patients on intense NT‐proBNP‐guided therapy. International Journal of Cardiology 2015;191:286‐93. 10.1016/j.ijcard.2015.04.282 25981371

[CD008966-bib-0049] Kaufmann BA, Min SY, Goetschalck K, Bernhei A, Pfistere M, Rocca HB. Evolution of left ventricular ejection fraction and left ventricular volumes in elderly heart failure patients under modern heart failure therapy: Influence of BNP‐guided therapy. American Heart Association. 2012; Vol. 126, issue 21 Suppl. 1:A17200.

[CD008966-bib-0050] Kaufmann BA, Min SY, Goetschalckx K, Bernheim AM, Buser PT, Pfisterer ME, et al. How reliable are left ventricular ejection fraction cut offs assessed by echocardiography for clinical decision making in patients with heart failure?. International Journal of Cardiovascular Imaging 2013;29(3):581‐8. 10.1007/s10554-012-0122-5 22965859

[CD008966-bib-0051] Maeder MT, Rickenbacher P, Rickli H, Abbuhl H, Gutmann M, Erne P, et al. N‐terminal pro brain natriuretic peptide‐guided management in patients with heart failure and preserved ejection fraction: findings from the Trial of Intensified versus standard medical therapy in elderly patients with congestive heart failure (TIME‐CHF). European Journal of Heart Failure 2013;15(10):1148‐56. 10.1093/eurjhf/hft076 23657728

[CD008966-bib-0052] Maeder MT, Rickli H, Pfisterer ME, Muzzarelli S, Ammann P, Fehr T, et al. Incidence, clinical predictors, and prognostic impact of worsening renal function in elderly patients with chronic heart failure on intensive medical therapy. American Heart Journal 2012;163(3):407‐14. 10.1016/j.ahj.2011.12.003 22424011

[CD008966-bib-0053] Muzzarelli S, Maeder MT, Toggweiler S, Rickli H, Nietlispach F, Julius B, et al. Frequency and predictors of hyperkalemia in patients >60 years of age with heart failure undergoing intense medical therapy. American Journal of Cardiology 2012;109(5):693‐8. 10.1016/j.amjcard.2011.10.027 22152974

[CD008966-bib-0054] Nasser Davarzani N, Wijk S Van, Maeder M, Burkart T, Rickenbacher P, Estlinbaum W, et al. NT‐ProBNP guided therapy reduces repeated hospitalizations‐results from TIME‐CHF. European Journal of Heart Failure 2014;Conference: Heart Failure Congress 2014 and the 1st World Congress on Acute Heart Failure Athens Greece. Conference Start: 20140517 Conference End: 20140520. Conference Publication: (var.pagings). 16:281.

[CD008966-bib-0055] Peeters JM, Sanders‐van Wijk S, Bektas S, Knackstedt C, Rickenbacher P, Nietlispach F, et al. Biomarkers in outpatient heart failure management; are they correlated to and do they influence clinical judgment?. Netherlands Heart Journal 2014;22(3):115‐21. 10.1007/s12471-013-0503-y PMC3931853 24338787

[CD008966-bib-0056] Pfisterer M, Buser P, Rickli H, Gutmann M, Erne P, Rickenbacher P, et al. BNP‐guided vs symptom‐guided heart failure therapy: the Trial of Intensified vs Standard Medical Therapy in Elderly Patients With Congestive Heart Failure (TIME‐CHF) randomized trial. JAMA 2009;301(4):383‐92. 10.1001/jama.2009.2 19176440

[CD008966-bib-0057] Rickenbacher P, Pfisterer M, Burkard T, Kiowski W, Follath F, Burckhardt D, et al. Baseline characteristics, adverse events and hospitalizations indicate an increased risk of death in patients with heart failure. An analysis of the TIME‐CHF trial. European Heart Journal 2011;Conference: European Society of Cardiology, ESC Congress 2011 Paris France. Conference Start: 20110827 Conference End: 20110831. Conference Publication: (var.pagings). 32:125‐6.

[CD008966-bib-0058] Rickenbacher P, Pfisterer M, Burkard T, Kiowski W, Follath F, Burckhardt D, et al. Why and how do patients with heart failure die? Insights from the TIME‐CHF trial. European Heart Journal 2011;Conference: European Society of Cardiology, ESC Congress 2011 Paris France. Conference Start: 20110827 Conference End: 20110831. Conference Publication: (var.pagings). 32:665.

[CD008966-bib-0059] Sanders‐van Wijk S, Maeder MT, Nietlispach F, Rickli H, Estlinbaum W, Erne P, et al. Long‐term results of intensified, N‐terminal‐pro‐B‐type natriuretic peptide‐guided versus symptom‐guided treatment in elderly patients with heart failure: five‐year follow‐up from TIME‐CHF. Circulation: Heart Failure 2014;7(1):131‐9. 10.1161/CIRCHEARTFAILURE.113.000527 24352403

[CD008966-bib-0060] Sanders‐van Wijk S, Asselt A van, Rickli H, Estlinbaum W, Erne P, Rickenbacher P, et al. Cost‐effectiveness of N‐terminal pro‐B‐type natriuretic‐guided therapy in elderly heart failure patients: results from TIME‐CHF (Trial of Intensified versus Standard Medical Therapy in Elderly Patients with Congestive Heart Failure). JACC Heart Failure 2013;1(1):64‐71. 10.1016/j.jchf.2012.08.002 24621800

[CD008966-bib-0061] Sanders‐van Wijk S, Empel V van, Davarzani N, Maeder MT, Handschin R, Pfisterer ME, et al. TIME‐CHF investigators. Circulating biomarkers of distinct pathophysiological pathways in heart failure with preserved vs. reduced left ventricular ejection fraction. European Journal of Heart Failure 2015;17(10):1006‐14. 10.1002/ejhf.414 26472682

[CD008966-bib-0062] Sanders‐van Wijk, S, Muzzarelli S, Neuhaus M, Kiencke S, Maeder M, Estlinbaum W, et al. Safety and tolerability of intensified, N‐terminal pro brain natriuretic peptide‐guided compared with standard medical therapy in elderly patients with congestive heart failure: results from TIME‐CHF. European Journal of Heart Failure 2013;15(8):910‐8. 10.1093/eurjhf/hft079 23666681

[CD008966-bib-0063] Wijk S Van, Bektas S, Muzzarelli S, Kiencke S, Maeder M, Estlinbaum W, et al. Impact of comorbidities on safety, tolerability and efficacy of intensified medical therapy in heart failure. European Heart Journal 2013;Conference: European Society of Cardiology, ESC Congress 2013 Amsterdam Netherlands. Conference Start: 20130831 Conference End: 20130904. Conference Publication: (var.pagings). 34:501.

[CD008966-bib-0064] Wijk S Van, Maeder MT, Nietlispach F, Rickli H, Estlinbaum W, Erne P, et al. Long‐term outcome of NT‐proBNP‐guided versus symptom‐guided therapy: Results from the TIME‐CHF study. European Journal of Heart Failure, Supplement 2011;Conference: Heart Failure Congress 2011 Gothenburg Sweden. Conference Start: 20110521 Conference End: 20110524. Conference Publication: (var.pagings). 10:S200.

[CD008966-bib-0065] Wijk S Van, Rickenbacher P, Tobler D, Nietlispach F, Buser P, Abbuehl H, et al. Galectin‐3 levels at baseline predict treatment response to drugs targeting the renin‐angiotensin‐aldosterone system and beta‐blockade in elderly patients with systolic heart failure. European Journal of Heart Failure 2014;Conference: Heart Failure Congress 2014 and the 1st World Congress on Acute Heart Failure Athens Greece. Conference Start: 20140517 Conference End: 20140520. Conference Publication: (var.pagings). 16:218‐9.

[CD008966-bib-0066] Wijk S Van, Asselt T Van, Maeder MT, Muzzarelli S, Erne P, Estlinbaum W, et al. Cost‐effectiveness of NT‐proBNP‐guided therapy in heart failure; results from the TIME‐CHF study. European Heart Journal 2011;Conference: European Society of Cardiology, ESC Congress 2011 Paris France. Conference Start: 20110827 Conference End: 20110831. Conference Publication: (var.pagings). 32:161.

[CD008966-bib-0067] Wijk S Van, Wijnen P, Bekers O, Tobler D, Rickli H, Erne P, et al. Genetic variation in the BNP‐gene: Effect on NT‐proBNP levels and results of NT‐proBNP‐guided therapy. European Journal of Heart Failure 2014;Conference: Heart Failure Congress 2014 and the 1st World Congress on Acute Heart Failure Athens Greece. Conference Start: 20140517 Conference End: 20140520. Conference Publication: (var.pagings). 16:125‐6.

[CD008966-bib-0068] Zurek M, Brunner‐La Rocca HB, Rickli HR, Gutmann MG, Handschin RH, Nietlispach FN, et al. Prognostic impact of systolic blood pressure and its changes during titration of medication in patients with chronic heart failure with reduced ejection fraction. European Heart Journal. 2014; Vol. Conference Publication: (var.pagings). 35:674.

[CD008966-bib-0069] Zurek M, Maeder MT, Rickli H, Muzzarelli S, Sanders‐van Wijk S, Abbuhl H, et al. Differential prognostic impact of resting heart rate in older compared with younger patients with chronic heart failure‐‐insights from TIME‐CHF. Journal of Cardiac Failure 2015;21(4):347‐54. 10.1016/j.cardfail.2014.12.014 25576682

[CD008966-bib-0070] Schou M, Gislason G, Videbaek L, Kober L, Tuxen C, Torp‐Pedersen C, et al. Effect of extended follow‐up in a specialized heart failure clinic on adherence to guideline recommended therapy: NorthStar Adherence Study. European Journal of Heart Failure 2014;16(11):1249‐55. 10.1002/ejhf.176 25311554

[CD008966-bib-0071] Schou M, Gustafsson F, Videbaek L, Andersen H, Toft J, Nyvad O, et al. Adding serial N‐terminal pro brain natriuretic peptide measurements to optimal clinical management in outpatients with systolic heart failure: a multicentre randomized clinical trial (NorthStar monitoring study). European Journal of Heart Failure 2013;15(7):818‐27. 10.1093/eurjhf/hft037 23507787

[CD008966-bib-0072] Schou M, Gustafsson F, Videbaek L, Markenvard J, Ulriksen H, Ryde H, et al. Design and methodology of the NorthStar Study: NT‐proBNP stratified follow‐up in outpatient heart failure clinics ‐‐ a randomized Danish multicenter study. American Heart Journal 2008;156(4):649‐55. 10.1016/j.ahj.2008.06.007 18946891

[CD008966-bib-0073] Shah MR, Califf RM, Nohria A, Bhapkar M, Bowers M, Mancini DM, et al. Erratum: The STARBRITE Trial: A Randomized, Pilot Study of B‐Type Natriuretic Peptide ‐ Guided Therapy in Patients With Advanced Heart Failure (Journal of Cardiac Failure (2011) 17 (613‐621)). Journal of Cardiac Failure 2011;17(9):788. 10.1016/j.cardfail.2011.04.012 21807321

[CD008966-bib-0074] Shah MR, Califf RM, Nohria A, Bhapkar M, Bowers M, Mancini DM, et al. The STARBRITE trial: a randomized, pilot study of B‐type natriuretic peptide‐guided therapy in patients with advanced heart failure.[Erratum appears in J Card Fail. 2011 Sep;17(9):788]. Journal of Cardiac Failure 2011;17(8):613‐21. 10.1016/j.cardfail.2011.04.012 21807321

[CD008966-bib-0075] Shah MR, Claise KA, Bowers MT, Bhapkar M, Little J, Nohria A, et al. Testing new targets of therapy in advanced heart failure: the design and rationale of the Strategies for Tailoring Advanced Heart Failure Regimens in the Outpatient Setting: BRain NatrIuretic Peptide Versus the Clinical CongesTion ScorE (STARBRITE) trial. American Heart Journal 2005;150(5):893‐8. 10.1016/j.ahj.2005.01.003 16290955

[CD008966-bib-0076] Shochat M, Shotan A, Dahan I, Shochat I, Levy Y, Asif A, et al. NT‐proBNP‐guided preemptive treatment of outpatients with chronic heart failure followed in a out hospital clinic. Journal of Cardiac Failure 2011;Conference: 15th Annual Scientific Meeting, Heart Failure Society of America Boston, MA United States. Conference Start: 20110918 Conference End: 20110921. Conference Publication:(var.pagings). 17 (8 SUPPL. 1):S56.

[CD008966-bib-0077] Shochat M, Shotan A, Kazatsker M, Asif A, Dahan I, Shochat, I, et al. NT‐proBNP‐guided preemptive treatment of outpatients with chronic heart failure followed in a out hospital clinic. Journal of Cardiac Failure 2012;Conference: 16th Annual Scientific Meeting of the Heart Failure Society of America, HFSA 2012 Seattle, WA United States. Conference Start: 20120909 Conference End: 20120912. Conference Publication::S58.

[CD008966-bib-0078] Koshkina D, Skvortsov A, Narusov O, Protasov V, Nasonova S, Masenko V, et al. NT‐proBNP‐guided treatment of high risk heart failure patients after acute decompensation. European Heart Journal. 2015; Vol. 36:153‐4.

[CD008966-bib-0079] Koshkina D, Skvortsov A, Protasov V, Narusov O, Masenko V, Tereschenko S. Biomarkers activity and the effect of NT‐proBNP guided therapy in high risk patients with chronic heart failure after acute decompensation. European Journal of Heart Failure. 2015; Vol. 17:142.

[CD008966-bib-0080] Skvortsov A, Koshkina D, Protasov V, Narusov O, Masenko V, Tereschenko S. Treatment optimisation of high risk heart failure patients after acute decompensation by NT‐proBNP monitoring. European Journal of Heart Failure. 2015; Vol. 17:421.

[CD008966-bib-0081] Skvortsov AA, Koshkina DE, Protasov VN, Narusov OY, Masenko VP, Tereschchenko SN. NT‐proBNP‐guided therapy reduces risk of death and hospitalisation in patients after decompensation of heart failure [Терапия под контролем NT‐proBNP снижает рисксмерти и частоту госпитализаций у больных последекомпенсации сердечной недостаточности]. Russian Heart Failure Journal 2015;16(4):204‐17.

[CD008966-bib-0082] Nicholls MG, Lainchbury JG, Richards AM, Troughton RW, Yandle TG. Brain natriuretic peptide‐guided therapy for heart failure. Annals of Medicine 2001;33(6):422‐7. 10.3109/07853890108995955 11585103

[CD008966-bib-0083] Troughton RW, Frampton CM, Yandle TG, Espiner EA, Nicholls MG, Richards AM. Treatment of heart failure guided by plasma aminoterminal brain natriuretic peptide (N‐BNP) concentrations. Lancet 2000;355(9210):1126‐30. 10.1016/s0140-6736(00)02060-2 10791374

[CD008966-bib-0084] Brunner‐La Rocca HP, Eurlings L, Richards AM, Januzzi JL, Pfisterer ME, Dahlstrom U, et al. Which heart failure patients profit from natriuretic peptide guided therapy? A meta‐analysis from individual patient data of randomised trials. European Journal of Heart Failure 2015;17(12):1252‐61. 10.1002/ejhf.401 26419999

[CD008966-bib-0085] ChiCTR‐TRC‐08000284. Randomized, double‐blind, placebo‐controlled study of recombinant B‐type natriuretic peptide in subjects with acute decompensated congestive heart failure. www.chictr.org/en/proj/show.aspx?proj=1111 (accessed 16 December 2014).

[CD008966-bib-0086] Cocco G, Jerie P. Assessing the benefits of natriuretic peptides‐guided therapy in chronic heart failure. Cardiology Journal 2015;22(1):5‐11. 10.5603/CJ.a2014.0041 24846509

[CD008966-bib-0087] Dandamudi S, Chen HH. The ASCEND‐HF trial: an acute study of clinical effectiveness of nesiritide and decompensated heart failure. Expert Review of Cardiovascular Therapy 2012;10(5):557‐63. 10.1586/erc.12.31 22651831

[CD008966-bib-0088] Vecchis R De, Esposito C, Biase G Di, Ariano C. B‐type natriuretic peptide. Guided vs conventional care in outpatients with chronic heart failure: a retrospective study. Minerva Cardioangiologica 2013;61(4):437‐49. 23846010

[CD008966-bib-0089] Somma S Di, Magrini L, Tabacco F, Marino R, Talucci V, Marrocco F, et al. Brain natriuretic peptide and N‐terminal pro‐B‐type natriuretic peptide show a different profile in response to acute decompensated heart failure treatment. Congestive Heart Failure 2008;14(5):245‐50. 10.1111/j.1751-7133.2008.00002.x 18983287

[CD008966-bib-0090] Dong SY, Dong M, Chen ZH, Sun J, Yang X, Zeng Q. Dynamic use of B‐Type natriuretic peptide‐guided acute coronary syndrome therapy. American Journal of the Medical Sciences 2014;348(4):283‐7. 10.1097/MAJ.0000000000000245 24762749

[CD008966-bib-0091] El‐Muayed M, Lavis VR, Safi HJ, Fuentes F. Use of glitazones in cardiac patients: a case for B‐type natriuretic peptide monitoring?. American Journal of Cardiology 2004;93(5):600‐2. 10.1016/j.amjcard.2003.11.025 14996586

[CD008966-bib-0092] Felker GM, Petersen JW, Mark DB. Natriuretic peptides in the diagnosis and management of heart failure. Canadian Medical Association Journal 2006;175(6):611‐7. 10.1503/cmaj.060236 PMC1559415 16966666

[CD008966-bib-0093] Gaggin HK, Truong QA, Rehman SU, Mohammed AA, Bhardwaj A, Parks KA, et al. Characterization and prediction of natriuretic peptide "nonresponse" during heart failure management: results from the ProBNP Outpatient Tailored Chronic Heart Failure (PROTECT) and the NT‐proBNP‐Assisted Treatment to Lessen Serial Cardiac Readmissions and Death (BATTLESCARRED) study. Congestive Heart Failure 2013;19(3):135‐42. 10.1111/chf.12016 23279139

[CD008966-bib-0094] Gonzalez S, Kilpatrick ES, Atkin SL. The biological variation of N‐terminal pro‐brain natriuretic peptide in postmenopausal women with type 2 diabetes: a case control study. PLoS ONE [Electronic Resource] 2012;7(11):e47191. 10.1371/journal.pone.0047191 PMC3494700 23152754

[CD008966-bib-0095] Green SM, Green JA, Januzzi JL. Natriuretic peptide testing for heart failure therapy guidance in the inpatient and outpatient setting. American Journal of Therapeutics 2009;16(2):171‐7. 10.1097/MJT.0b013e318172797f 19300043

[CD008966-bib-0096] JernbergT, Lindahl B, Siegbahn A, Andren B, Frostfeldt G, Lagerqvist B, et al. N‐terminal pro‐brain natriuretic peptide in relation to inflammation, myocardial necrosis, and the effect of an invasive strategy in unstable coronary artery disease. Journal of the American College of Cardiology 2003;42(11):1909‐16. 10.1016/j.jacc.2003.07.015 14662251

[CD008966-bib-0097] Kociol RD, McNulty SE, Hernandez AF, Lee K L, Redfield MM, Tracy RP, et al. Markers of congestion, symptom relief and clinical outcomes among patients hospitalized with acute heart failure: Data from the diuretic optimal strategy evaluation in acute heart failure study. Journal of the American College of Cardiology 2011;Conference: 60th Annual Scientific Session of the American College of Cardiology and i2 Summit: Innovation in Intervention, ACC.11 New Orleans, LA United States. Conference Start: 20110402 Conference End: 20110405. Conference Publication::E220.

[CD008966-bib-0098] Koitabashi T, Inomata T, Niwano S, Nishii M, Takeuchi I, Nakano H, et al. Distinguishable optimal levels of plasma B‐type natriuretic peptide in heart failure management based on complicated atrial fibrillation. International Heart Journal 2005;46(3):453‐64. 10.1536/ihj.46.453 16043941

[CD008966-bib-0099] Komajda M. REVIVE II (randomized multicenter evaluation of intravenous levosimendan efficacy). Clinical Cardiology 2006;29:43.

[CD008966-bib-0100] Krackhardt F, Duengen HD, Schlattmann P, Kehrt K, Hassfeldt S, Dietz R, et al. NT‐proBNP predicts long‐term risk of cardiac death in patients with dilative cardiomyopathy: A ten‐year follow‐up trial. Journal of the American College of Cardiology 2008;51(10):A249‐A.

[CD008966-bib-0101] Krackhardt F, Dungen HD, Trippel TD, Inkrot S, Tscholl V, Schlattmann P, et al. N‐terminal pro‐B‐type natriuretic peptide and long‐term mortality in non‐ischaemic cardiomyopathy. Wiener Klinische Wochenschrift 2011;123(23‐24):738‐42. 10.1007/s00508-011-0092-y 22105112

[CD008966-bib-0102] Ledwidge M, Gallagher J, Conlon C, Tallon E, O'Connell E, Dawkins I, et al. Natriuretic peptide‐based screening and collaborative care for heart failure: The STOP‐HF randomised trial. JAMA 2013;310(1):66‐74. 10.1001/jama.2013.7588 23821090

[CD008966-bib-0103] Leuchte HH, Holzapfel M, Neurohr C, Vogeser M, Behr J. Characterization of brain natriuretic peptide in long‐term follow‐up of pulmonary arterial hypertension. Chest 2005;128(4):2368‐74. 10.1378/chest.128.4.2368 16236896

[CD008966-bib-0104] Li N, Li Y, Wang F, Jiang W, Huang J, Xu Z, et al. Does NT‐proBNP remain a sensitive biomarker for chronic heart failure after administration of a beta‐blocker?. Clinical Cardiology 2007;30(9):469‐74. 10.1002/clc.20150 PMC6653750 17803204

[CD008966-bib-0105] Lindahl B, Lindback J, Jernberg T, Johnston N, Stridsberg M, Venge P, et al. Serial analyses of N‐terminal pro‐B‐type natriuretic peptide in patients with non‐ST‐segment elevation acute coronary syndromes ‐ A fragmin and fast revascularisation during instability in coronary artery disease (FRISC)‐II substudy. Journal of the American College of Cardiology 2005;45:533‐41. 10.1016/j.jacc.2004.10.057 15708700

[CD008966-bib-0106] Luchner A, Mockel M, Spanuth E, Mocks J, Peetz D, Baum H, et al. N‐terminal pro brain natriuretic peptide in the management of patients in the medical emergency department (PROMPT): correlation with disease severity, utilization of hospital resources, and prognosis in a large, prospective, randomized multicentre trial. European Journal of Heart Failure 2012;14:259‐67. 10.1093/eurjhf/hfr171 22265921

[CD008966-bib-0107] Maisel A, Barnard D, Jaski B, Frivold G, Marais J, Azer M, et al. Primary results of the HABIT Trial (heart failure assessment with BNP in the home). Journal of the American College of Cardiology 2013;61(16):1726‐35. 10.1016/j.jacc.2013.01.052 23500322

[CD008966-bib-0108] McNairy M, Gardetto N, Clopton P, Garcia A, Krishnaswamy P, Kazanegra R, et al. Stability of B‐type natriuretic peptide levels during exercise in patients with congestive heart failure: implications for outpatient monitoring with B‐type natriuretic peptide. American Heart Journal 2002;143(3):406‐11. 10.1067/mhj.2002.120148 11868044

[CD008966-bib-0109] Miller WL, Hartman KA, Hodge DO, Hartman S, Struck J, Morgenthaler NG, et al. Response of novel biomarkers to BNP infusion in patients with decompensated heart failure: a multimarker paradigm. Journal of Cardiovascular Translational Research 2009;2(4):526‐35. 10.1007/s12265-009-9121-x 20560012

[CD008966-bib-0110] Murdoch DR, McDonagh TA, Byrne J, Blue L, Farmer R, Morton JJ, et al. Titration of vasodilator therapy in chronic heart failure according to plasma brain natriuretic peptide concentration: randomised comparison of the haemodynamic and neuroendocrine effects of tailored versus empirical therapy. Amercian Heart Journal 1999;138(6):1126‐32. 10.1016/s0002-8703(99)70079-7 10577444

[CD008966-bib-0111] NCT00206856. Rapid Assessment of Bedside BNP In Treatment of Heart Failure (RABBIT). clinicaltrials.gov/ct2/show/NCT00206856 (accessed 16 December 2014).

[CD008966-bib-0112] NCT00622531. Serial BNP Testing for heart failure management (USE‐BNP). clinicaltrials.gov/ct2/show/NCT00622531?term=NCT00622531&rank=1 (accessed 16 December 2014).

[CD008966-bib-0113] NCT01299350. Nt‐proBNP versus clinical guided discharge in acute heart failure. clinicaltrials.gov/ct2/show/NCT01299350 (accessed 16 December 2014).

[CD008966-bib-0114] Pascual‐Figal DA, Domingo M, Casas T, Gich I, Ordonez‐Llanos J, Martinez P, et al. Usefulness of clinical and NT‐proBNP monitoring for prognostic guidance in destabilized heart failure outpatients. European Heart Journal 2008;29(8):1011‐8. 10.1093/eurheartj/ehn023 18263871

[CD008966-bib-0115] Tang WHW, Francis GS. The difficult task of evaluating how to monitor patients with heart failure. Journal of Cardiac Failure 2005;11(6):422‐4. 10.1016/j.cardfail.2005.05.001 16105632

[CD008966-bib-0116] Troughton RW, Richards AM, Yandle TG, Nicholls G. Routine measurement of natriuretic peptide to guide the diagnosis and management of chronic heart failure. Circulation 2004;109(25):e325‐6; author reply e‐6. 15232832

[CD008966-bib-0117] Valle R, Aspromonte N, Giovinazzo P, Carbonieri E, Chiatto M, Tano G di, et al. B‐type natriuretic Peptide‐guided treatment for predicting outcome in patients hospitalized in sub‐intensive care unit with acute heart failure. Journal of Cardiac Failure 2008;14(3):219‐24. 10.1016/j.cardfail.2007.10.009 18381185

[CD008966-bib-0118] Wasywich CA, Whalley GA, Walsh HA, Gamble GD, Doughty RN. Changes in tissue‐Doppler echocardiographic assessment of left ventricular filling during NT‐proBNP guided heart failure treatment titration: a pilot study. Heart, Lung & Circulation 2009;18(1):38‐44. 10.1016/j.hlc.2008.07.002 18818124

[CD008966-bib-0119] Felker GM, Ahmad T, Anstrom KJ, Adams KF, Cooper LS, Ezekowitz JA, et al. Rationale and design of the GUIDE‐IT study: Guiding Evidence Based Therapy Using Biomarker Intensified Treatment in Heart Failure. JACC Heart Failure 2014;2(5):457‐65. 10.1016/j.jchf.2014.05.007 PMC4194159 25194287

[CD008966-bib-0120] NCT02110433. Heart Failure Educational and Follow up Platform (HELP). https://clinicaltrials.gov/ct2/show/NCT02110433 (accessed 28 January 2016).

[CD008966-bib-0121] Metra M, Pagani F, Lazzarini V, Bettari L, Bonetti G, Bugatti S, et al. Acute heart failure (AHF) is associated with poor prognosis. Giornale Italiano di Cardiologia 2011;Conference: 72 Congresso Nazionale Della Societa Italiana di Cardiologia Rome Italy. Conference Start: 20111210 Conference End: 20111212. Conference Publication:(Conference: 72 Congresso Nazionale Della Societa Italiana di Cardiologia Rome Italy. Conference Start: 20111210 Conference End: 20111212. Conference Publication:):e37.

[CD008966-bib-0122] NCT00601679. Improvement in Clinical Outcomes of Patients With Chronic Heart Failure Using Serial NT‐proBNP Monitoring: The EX‐IMPROVE‐CHF Study. http://clinicaltrials.gov/ct2/results?term=eximprovechf (accessed 16 December 2014).

[CD008966-bib-0123] Saraya M, Kassem H, Salah Eldin H. Adding brain natriuretic peptide, ultrasound lung comets or tissue Doppler to clinical guidance in reducing heart failure hospitalisation. European Heart Journal. 2015; Vol. 36:504.

[CD008966-bib-0124] Stienen S, Salah K, Moons AH, Bakx AL, Pol PE van, Schroeder‐Tanka JM, et al. Rationale and design of PRIMA II: A multicenter, randomized clinical trial to study the impact of in‐hospital guidance for acute decompensated heart failure treatment by a predefined NT‐PRoBNP target on the reduction of readmIssion and Mortality rAtes. American Heart Journal 2014;168(1):30‐6. 10.1016/j.ahj.2014.04.008 24952857

[CD008966-bib-0125] Atisha D, Bhalla MA, Morrison LK, Felicio L, Clopton P, Gardetto N, et al. A prospective study in search of an optimal B‐natriuretic peptide level to screen patients for cardiac dysfunction. American Heart Journal 2004;148(3):518‐23. 10.1016/j.ahj.2004.03.014 15389242

[CD008966-bib-0126] Balion C, McKelvie R, Don‐Wauchope AC, Santaguida PL, Oremus M, Keshavarz H, et al. B‐type natriuretic peptide‐guided therapy: a systematic review. Heart Failure Review 2014;19:553‐64. 10.1007/s10741-014-9451-x 25074674

[CD008966-bib-0127] Chen WC, Tran KD, Maisel AS. Biomarkers in heart failure. Heart 2010;96(4):314‐20. 10.1136/hrt.2008.151639 20194212

[CD008966-bib-0128] Clerico A, Fontana M, Zyw L, Passino C, Emdin M. Comparison of the diagnostic accuracy of brain natriuretic peptide (BNP) and the N‐terminal part of the propeptide of BNP immunoassays in chronic and acute heart failure: a systematic review. Clinical Chemistry 2007;53(5):813‐22. [PUBMED: 17384013] 17384013 10.1373/clinchem.2006.075713

[CD008966-bib-0129] DeBeradinis B, Januzzi JL. Use of biomarkers to guide outpatient therapy of heart failure. Current Opinion Cardiology 2012;27:661‐8. 10.1097/HCO.0b013e3283587c4d 22941124

[CD008966-bib-0130] Vecchis R De, Esposito C, Cantatrione S. Natriuretic peptide‐guided therapy. Herz 2013;38:618‐28. 10.1007/s00059-013-3772-8 23588602

[CD008966-bib-0131] Vecchis R De, Esposito C, Biase G Di, Ariano C, Giasi A, Cioppa C. B‐type natriuretic peptide‐guided versus symptom‐guided therapy in outpatients with chronic heart failure: a systematic review with meta‐analysis. Journal of Cardiovascular Medicine 2014;15(2):122‐34. 10.2459/JCM.0b013e328364bde1 24522083

[CD008966-bib-0132] Doust JA, Pietrzak E, Dobson A, Glasziou P. How well does B‐type natriuretic peptide predict death and cardiac events in patients with heart failure: systematic review. BMJ (Clinical research ed.) 2005;330(7492):625. [PUBMED: 15774989] 15774989 10.1136/bmj.330.7492.625 PMC554905

[CD008966-bib-0133] Felker GM, Hasselblad V, Hernandez AF, O’Connor CM. Biomarker‐guided therapy in chronic heart failure:A meta‐analysis of randomised controlled trials. American Heart Journal 2009;158:422‐30. 10.1016/j.ahj.2009.06.018 19699866

[CD008966-bib-0134] Higgins JPT, Green S (editors). Cochrane Handbook for Systematic Reviews of Interventions Version 5.1.0 [updated March 2011]. The Cochrane Collaboration, 2011, Available from www.cochrane‐handbook.org.

[CD008966-bib-0135] Ichiki T, Huntley BK, Burnett JC. BNP molecular forms and processing by the cardiac serine protease corin. Advances in Clinical Chemistry 2013;61:1‐31. 10.1016/b978-0-12-407680-8.00001-4 PMC4522930 24015598

[CD008966-bib-0136] Lefebvre C, Manheimer E, Glanville J. Chapter 6: Searching for studies. Higgins JPT, Green S (editors). Cochrane Handbook for Systematic Reviews of Interventions Version 5.1.0 (updated March 2011). The Cochrane Collaboration, 2011, Available from www.cochrane‐handbook.org.

[CD008966-bib-0137] Li P, Luo Y, Chen Y. B‐type natriuretic peptide‐guided chronic heart failure therapy: a meta‐analysis of 11 randomised controlled trials. Heart, Lung and Circulation 2013;22(10):852‐60. 10.1016/j.hlc.2013.03.077 23602555

[CD008966-bib-0138] Li Y, Pei H, Zhou X, Wu Y. Efficacy, modifiable factors to efficacy, safety of B‐type natriuretic peptide‐guided heart failure therapy: A meta‐analysis. Cardiology (Switzerland). 2014:66.

[CD008966-bib-0139] McMurray JJ, Adamopoulos S, Anker SD, Auricchio A, Böhm M, Dickstein K, et al. ESC Guidelines for the diagnosis and treatment of acute and chronic heart failure 2012. European Heart Journal 2012;33(14):1797‐847. 10.1093/eurheartj/ehs104 22611136

[CD008966-bib-0140] National Clincial Guideline Collaborating Centre. Chronic heart failure: management of chronic heart failure in adults in primary and secondary care. http://www.nice.org.uk/CG108 (accessed 25 June 2010).

[CD008966-bib-0141] National Clincial Guideline Collaborating Centre. Acute heart failure: diagnosis and management. https://www.nice.org.uk/guidance/cg187 (accessed 17 Feb 2016).

[CD008966-bib-0142] Owan TE, Hodge DO, Herges RM, Jacobsen SJ, Roger VL, Redfield MM. Trends in prevalence and outcome of heart failure with preserved ejection fraction. New England Journal of Medicine 2006;355(3):251‐9. 10.1056/NEJMoa052256 16855265

[CD008966-bib-0143] Porapakkham P, Porapakkham P, Zimmet H, Billah B, Krum H. B‐Type natriuretic peptide‐guided heart failure therapy: A meta‐analysis. Archives of Internal Medicine 2010;170(6):507‐14. 10.1001/archinternmed.2010.35 20308637

[CD008966-bib-0144] Richards AM, Troughton RW. Use of natriuretic peptides to guide and monitor heart failure therapy. Clinical Chemistry 2012;58(1):62‐71. 10.1373/clinchem.2011.165704 22086970

[CD008966-bib-0145] Savarese G, Trimarco B, Dellegrottaglie S, Prastaro M, Gambardella F, Rengo G, et al. Natriuretic peptide‐guided therapy in chronic heart failure: a meta‐analysis of 2,686 patients in 12randomized trials. PLoS One 2013;8(3):e96706. 10.1371/journal.pone.0058287 PMC3589263 23472172

[CD008966-bib-0146] Troughton RW, Frampton CM, Brunner‐La Rocca HP, Pfisterer M, Eurlings LWM, Erntell H, et al. Effect of B‐type natriuretic peptide‐guided treatment of chronic heart failure on total mortality and hospitalisation: an individual patient meta‐analysis. European Heart Journal 2014;35:1559‐67. 10.1093/eurheartj/ehu090 PMC4057643 24603309

[CD008966-bib-0147] Xin W, Lin Z, Mi S. Does B‐type natriuretic peptide‐guided therapy improve outcomes in patients with chronic heart failure? A systematic review and meta‐analysis of randomised controlled trials. Heart Failure Review 2015;20:69‐80. 10.1007/s10741-014-9437-8 24888383

[CD008966-bib-0148] Kearley KE, Wright FL, Tyndel S, Roberts NW, Perera R, Glasziou PP, et al. B‐type natriuretic peptide‐guided treatment for heart failure. Cochrane Database of Systematic Reviews 2011, Issue 1. [DOI: 10.1002/14651858.CD008966] PMC5449577 28102899

